# Quantifying the contributions of cardiovascular risk factors to cardiovascular disease trends in 21st century Japan: a microsimulation study

**DOI:** 10.1016/j.lanwpc.2025.101623

**Published:** 2025-07-08

**Authors:** Soshiro Ogata, Eri Kiyoshige, Yusuke Yoshikawa, Koji Iihara, Hitoshi Fukuda, Masanobu Ishii, Kenichi Tsujita, Anna Head, Brendan Collins, Martin O'Flaherty, Kunihiro Nishimura, Chris Kypridemos

**Affiliations:** a Department of Preventive Medicine and Epidemiology, National Cerebral and Cardiovascular Center, Suita, Osaka, 564-8565, Japan; b Department of Biostatistics, National Cerebral and Cardiovascular Center, Suita, Osaka, 564-8565, Japan; c National Cerebral and Cardiovascular Center Hospital, Suita, Osaka, 564-8565, Japan; d Department of Neurosurgery, Kochi Medical School, Nankoku, Kochi, 783-8505, Japan; e Department of Cardiovascular Medicine, Graduate School of Medical Sciences, Kumamoto University, Kumamoto, Kumamoto, 860-8556, Japan; f Department of Medical Information Science, Graduate School of Medical Sciences, Kumamoto University, Kumamoto, Kumamoto, 860-8556, Japan; g Department of Public Health, Policy and Systems, University of Liverpool, Liverpool, L69 3GF, United Kingdom

**Keywords:** Public health, Cardiovascular risk factors, The national burdens of cardiovascular disease, Coronary heart disease, Stroke, Microsimulation study, All-cause deaths, Quality-adjusted life years, Medical costs, Indirect costs, Japan

## Abstract

**Background:**

Recent stagnation or worsening trends in cardiovascular disease (CVD) risk factors, including low-density lipoprotein cholesterol (LDL-c) and obesity, might slow the decline in Japan's CVD burden. We aimed to quantify the impact of national changes in CVD risk factor distributions on Japan's CVD burden from 2001 to 2019.

**Methods:**

We conducted a microsimulation study with counterfactual analysis using IMPACT_NCD-JPN_, a validated model based on real-world data. It simulated a synthetic Japanese population (ages 30–99) from 2001 to 2019 using life-course data on seven CVD risk factors, estimating CVD incidence, mortality, and healthcare economics for synthetic individuals. The base-case reflected observed trends; counterfactual scenarios assumed 2001 levels persisted. Primary outcome was national CVD incidence (stroke and coronary heart disease).

**Findings:**

From 2001 to 2019, systolic blood pressure (SBP) and smoking declined markedly (men/women) by 6·8/7·2 mmHg and 18·4/6·8%, respectively, while LDL-c, HbA1c, body mass index (BMI), physical activity (PA), and fruit/vegetable (FV) consumption showed smaller or adverse trends. Under the base-case and counterfactual scenarios, IMPACT_NCD-JPN_ estimated CVD incidence and quantified the differences between the scenarios. The changes in the CVD risk factors prevented or postponed 840,000 (95% uncertainty interval: 540,000–1,300,000) national CVD cases, cumulative from 2001 to 2019. Individual contributions were: SBP 540,000; smoking 280,000; LDL-c 27,000; HbA1c 7900; BMI −15,000; PA −16,000; and FV consumption −11,000.

**Interpretation:**

SBP and smoking reductions drove most CVD burden declines in Japan (2001–2019). Modest benefits came from LDL-c and HbA1c, while rising BMI, and low PA and FV intake partly offset these benefits.

**Funding:**

10.13039/501100001691JSPS KAKENHIJP22K17821, JP25K02863; the 10.13039/501100003478Ministry of Health, Labour and Welfare Comprehensive Research on Life-Style Related 22FA1015, 24FA1015.

**Research in context**

**Evidence before this study**

Previous research has shown that Japan achieved substantial reductions in coronary heart disease (CHD) and stroke mortality during the latter half of the 20th century, primarily due to declining systolic blood pressure (SBP) and reduced smoking prevalence. However, since the early 21st century, unfavourable trends have emerged—most notably, increasing body mass index (BMI), decreasing physical activity, and reduced fruit and vegetable (FV) consumption, along with stagnating levels of low-density lipoprotein cholesterol (LDL-c) and glycated haemoglobin (HbA1c). We systematically searched PubMed using Medical Subject Headings (MeSH) to identify previous studies published up to February 2025. Our search combined terms related to: 1) health policy, health promotion, and programme evaluation (“Health Policy”[MeSH] OR “Policy Making”[MeSH] OR “Program Evaluation”[MeSH] OR “Primary Prevention”[MeSH] OR “Health Education”[MeSH] OR “Risk Reduction Behavior”[MeSH]); 2) cardiovascular risk factors and preventive interventions (“Risk Factors”[MeSH] OR “Obesity”[MeSH] OR “Body Mass Index”[MeSH] OR “Exercise”[MeSH] OR “Vegetables”[MeSH] OR “Fruit”[MeSH] OR “Smoking”[MeSH] OR “Blood Pressure”[MeSH] OR “Hypertension”[MeSH] OR “Blood Glucose”[MeSH] OR “Glycated Hemoglobin”[MeSH] OR “Diabetes Mellitus, Type 2”[MeSH] OR “Cholesterol”[MeSH] OR “Dyslipidemias”[MeSH] OR “Health Planning”[MeSH] OR “Early Medical Intervention”[MeSH] OR “Mass Screening”[MeSH]); and 3) cardiovascular diseases (“Cardiovascular Diseases”[MeSH]). The search was further refined to include studies using simulation methods (“simulation” OR “microsimulation”) or epidemiological studies specifically from Japan (“Japan/epidemiology”[MeSH]).

Very few studies have estimated how changes in the national distribution of multiple modifiable CVD risk factors have collectively contributed to Japan's overall CVD burden—including CHD and stroke incidence, case-years, all-cause mortality, direct and indirect CVD-related healthcare costs, and quality-adjusted life-years (QALYs). Most previous modelling studies in Japan have evaluated the contribution of individual risk factors in isolation, rarely assessing their combined effects. For example, a recent simulation projected that if mean salt intake remained at 2019 levels over the next 10 years, cumulative incident cases would reach approximately 2·0 million for CHD and 2·6 million for stroke, with associated costs of USD 61·6 billion for CHD and USD 104·6 billion for stroke. Our earlier model, IMPACT_FIRST-JPN_, evaluated the combined impact of changes in multiple CVD risk factors on national CHD mortality, but with key limitations. Firstly, it considered only two time points (1980 and 2012), ignoring cumulative or continuous trends over time. Secondly, the model focused exclusively on CHD mortality as its outcome, without incorporating CHD and stroke incidence, case-years lived with disease, all-cause mortality, QALYs, or direct and indirect CVD costs.

**Added value of this study**

To our knowledge, the present study is the first to comprehensively integrate multiple modifiable CVD risk factors—SBP, smoking, LDL-c, HbA1c, BMI, physical activity, and FV consumption—using our validated microsimulation model (IMPACT_NCD-JPN_) and counterfactual analysis. We quantified how changes in the national distribution of these risk factors contributed to trends in the national burden of CVD among Japanese adults from 2001 to 2019, including CHD and stroke incidence, case-years, all-cause mortality, direct and indirect CVD costs, and QALYs.

The present IMPACT_NCD-JPN_ quantified that the observed changes in the national-level distribution of the seven key CVD risk factors prevented or postponed cumulative CHD cases of 290,000 (140,000–510,000) for men and 210,000 (99,000–400,000) for women, and cumulative stroke cases of 280,000 (150,000–460,000) for men and 190,000 (110,000–310,000) for women at the national level in Japan between 2001 and 2019. These CPPs would result in cumulatively preventing or postponing all-cause deaths of 550,000 (460,000–680,000) for men and 290,000 (220,000–390,000) for women, cumulatively gaining net QALYs of 1,600,000 (960,000–2,400,000) for men and 1,300,000 (950,000–1,900,000) for women. These would cumulatively save net direct and indirect costs of CVD, respectively, by 4·9B (3·5B–6·5B) and 130B (98B–180B) USD in men, and 3·7B (2·7B–5·1B) and 25B (16B–34B) USD in women. Decreases during the period in SBP adjusted for antihypertensive medication use and smoking prevalence mainly explained this reduction in CHD and stroke burden; in contrast, increases in BMI and decreases in physical activity and FV consumption partially offset the reduction.

Compared to our previous IMPACT_FIRST-JPN_, which assessed non-cumulative changes solely in national CHD mortality between only two time points (1980 and 2012), the current IMPACT_NCD-JPN_ model incorporates more data points, covers a broader range of outcomes including costs and QALYs, and offers improved quantification of uncertainty.

**Implications of all the available evidence**

The present results show that changes in the national distribution of seven CVD risk factors between 2001 and 2019 prevented or postponed CHD and stroke cases, all-cause deaths, and associated costs and increased QALYs in Japan. Reduced SBP and smoking cessation mainly drove these benefits, while stagnant LDL-c and HbA1c contributed modestly. However, rising BMI and declines in physical activity and FV consumption partially offset them. Previous studies also showed that decreases in CVD deaths and all-cause deaths attributable to CVD risk factors, including SBP and smoking, have stagnated worldwide, including in Japan, especially from 2010 to 2019. Those previous and present findings would suggest the importance of continued CVD risk factor control in sustaining the improvements observed during the early 21st century, which would be helpful to address the anticipated increase in CVD burden due to its ageing population, with projections showing that 35% of the population will be over 65 years old by 2040. Note that we do not assume that the same magnitude of effects which IMPACT_NCD-JPN_ estimated will occur in the future, especially as further reductions in SBP may yield diminishing returns. Therefore, we caution against directly extrapolating these results beyond the study period. Future studies could build on our findings by modelling alternative scenarios—such as maintaining current trends or implementing specific policy interventions—projected through to 2040, to estimate their potential impact on the CVD burden.

The present quantifications provide policy decision-makers insights for evidence-based CVD policymaking, especially related to the Japanese National Plan for Promotion of Measures Against Cerebrovascular and Cardiovascular Disease. Additionally, regular use of quantitative microsimulation models like IMPACT_NCD-JPN_ can guide policy decisions, optimise resource allocation, and improve population cardiovascular health in ageing societies globally.

## Introduction

To what extent have the observed national changes in key cardiovascular disease (CVD) risk factors contributed to the trends in CVD burden in Japan since the early 21st century? In the latter half of the 20th century, Japan had one of the lowest CVD age-standardised mortality rates globally,^1^ with a declining trend outpacing Western countries.^2^ This historic decline was primarily due to improvements in public health and the healthcare system. Entering the 21st century, Japan has implemented successful CVD prevention, particularly targeting hypertension and smoking, through medical guideline updates,^3^ increased awareness among healthcare providers,^4^ and public health initiatives promoting healthier diets,^5^ four tobacco tax increases since 2001,^6^ and stricter tobacco control policies for adolescents.^7^^,^^8^

However, progress in other CVD risk factors has slowed since the early 21st century, with little improvement in low-density lipoprotein cholesterol (LDL-c) and rising obesity prevalence.^9^ Meanwhile, crude mortality from heart and vascular diseases (including coronary heart disease [CHD], stroke, and hypertensive heart disease) has increased,^10^ partly due to population ageing. However, ageing alone does not fully explain this trend.^11^ Quantifying the impact of modifiable risk factors remains essential for developing effective public health strategies to address these challenges.

Despite these complexities, few studies have quantified how changes in the national distribution of CVD risk factors since the early 21st century have impacted trends in CVD incidence, mortality, costs, and quality-adjusted life years (QALYS). This gap may result in an insufficient evaluation of public health policies and an oversight of key CVD drivers. Thus, the present study aimed to quantify how changes in the national distribution of key CVD risk factors contributed to national trends in CVD burden (CHD and stroke incidence, mortality, costs, and QALYs) from 2001 to 2019 in the adult population of Japan using microsimulation and counterfactual analyses. Observed trends were systematically compared with counterfactual scenarios in which population exposure to key CVD risk factors (both individually and in combination) was held constant at 2001 levels by age and sex. Key CVD risk factors included systolic blood pressure (SBP), smoking, LDL-c, glycated haemoglobin (HbA1c), body mass index (BMI), physical activity, and fruit and vegetable (FV) intake.

## Methods

### Overview of study design and model structure

**Fig. 1:**
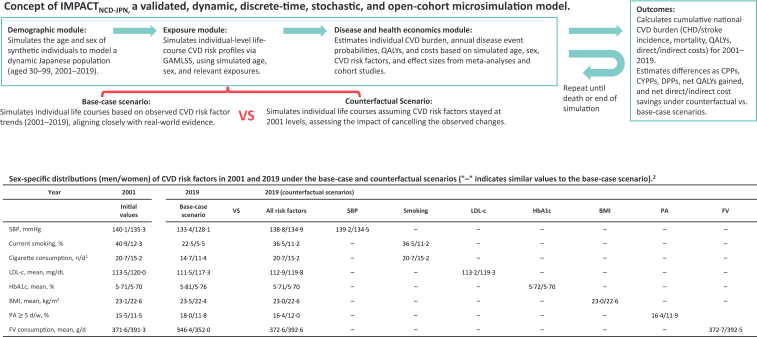
**Overview of study design, including counterfactual analysis and IMPACT_NCD-JPN_ structure.** Abbreviations: CVD, cardiovascular diseases; GAMLSS, Generalised Additive Models for Location, Scale, and Shape; CHD, coronary heart disease; QALYs, quality-adjusted life years; CPPs, cases prevented or postponed; CYPPs, case-years prevented or postponed; DPPs, deaths prevented or postponed; SBP, systolic blood pressure; LDL-c, low density lipoprotein cholesterol; BMI, body mass index; PA, physical activity; FV, fruits and vegetables. ^1^Cigarette consumption for current smokers. ^2^Detailed age- and sex-stratified distributions are presented in Supplementary Figure S1 in Supplementary Results. IMPACT_NCD-JPN_ simulated synthetic individuals with specific age (1-year), sex, year (1-year), and values for seven CVD risk factors: SBP, smoking status, LDL-c, HbA1c, BMI, PA, and FV consumption. Each CVD risk factor was modelled based on age, sex, year, and, where applicable, selected relevant CVD risk factors among the seven. A brief structure of IMPACT_NCD-JPN_ is also presented in the Technical Appendix “Introduction” in Supplementary Methods.

Fig. 1 shows an overview of the study design, including counterfactual analysis and the IMPACT_NCD-JPN_ structure. The present study conducted a microsimulation analysis, modelling a dynamic synthetic population of Japanese aged 30–99 from 2001 to 2019. To accomplish this, we developed IMPACT_NCD-JPN_, a validated dynamic, discrete-time, stochastic, and open-cohort microsimulation based on the IMPACT_NCD_ microsimulation framework, which we had previously developed in the UK.^12^

IMPACT_NCD-JPN_ consists of three interconnected modules, namely, a ‘demographic’ module, an ‘exposure’ module, and a ‘disease and health economics’ module. A brief summary essential to interpreting the present results is provided in the following subsections of the Methods section in the main text and Technical Appendix “Introduction” Figure 1-1 in Supplementary Methods. Technical details, including the validation and key assumptions, are reported in the Technical Appendix and our previous papers.^12^ For instance, the “Demographic module”, “Exposure module”, and “Clustering of risk factors” sections provide further information on how age, sex, years, and CVD risk factors are simulated over time and how correlations between them. The “Disease module”, including the “Disease incidence” and “Mortality” sections, outlines how CVD outcomes are generated and linked to the simulated age, sex, and CVD risk profiles.

### Model inputs and data sources for individual-level simulation of demographics, CVD risk factors, and outcomes

IMPACT_NCD-JPN_ utilises Japanese datasets. Below, we present a brief description of the model inputs, while the Technical Appendix outlines the details of each dataset. For the demographic module (Technical Appendix “Demographic module” in Supplementary Methods), we sourced data on population size between 2001 and 2019 by sex (as a biological variable) and age (in one-year increments from 30 to 99 years old) for Japan from the Population Census and Population Estimates provided by the Statistics Bureau, Ministry of Internal Affairs and Communications.^13^^,^^14^

For the exposures module (Technical Appendix “Exposure module” in Supplementary Methods), we sourced the individual-level data on the aforementioned established CVD risk factor exposures from the National Health and Nutrition Survey (NHNS) in participants aged between 20 and 99 from 1995 to 2019 or any available years the risk factor was measured if not available throughout the period.^15^ IMPACT_NCD-JPN_ simulates lag times of up to 10 years between exposure and disease; hence, we also incorporated data from participants younger than 30 and from years prior to 2001. We modelled the individual-level CVD risk factors from the NHNS data using Generalised Additive Models for Location, Scale, and Shape (GAMLSS) to simulate individual-level life course profiles of multiple CVD risk factors while conditioning on age, sex, and relevant behavioural and biological CVD risk factors (Technical Appendix “Exposure module” and Supplementary Material B in Supplementary Methods).

For the disease module (Technical Appendix “Disease module” and “population-attributable risk fraction [PARF]”, and Supplementary Material A in Supplementary Methods), we obtained relative risks (RRs) from reported meta-analyses or high-quality cohort studies. Additionally, we gathered incidence and prevalence proportions for CHD and stroke from estimates of “ischaemic heart disease” (ICD-10 cods I20–I25) and “stroke” (ICD-10 codes I60–I69), respectively, as reported in the Global Burden of Diseases, Injuries, and Risk Factors Study (GBD) for the initial year, 2001.^16^ We collected mortality proportions for CHD, stroke, and non-modelled deaths (i.e., all deaths excluding CHD and stroke deaths) from observed reports of vital statistics in Japan provided by the Ministry of Health, Labour and Welfare for the initial year, 2001.^17^ CHD mortality included deaths coded under “acute myocardial infarction” and “other ischaemic heart diseases” (ICD-10 codes I20–I22, I24, and I25). Although ICD-10 code I23 (certain current complications following acute myocardial infarction) is excluded from Japan's official mortality tabulations, deaths originally certified as I23 are routinely reclassified to I21 or I22 (acute myocardial infarction or subsequent myocardial infarction) as the underlying cause of death, in accordance with coding guidelines. This classification practice, based on the ICD-10 (2013 revision), ensures no substantial loss of mortality data due to this exclusion. Stroke mortality was derived from deaths coded under “stroke” (ICD-10 codes I60–I69). The module uses these inputs to estimate annual individualised CHD and stroke incident risk for healthy synthetic individuals, annual individualised CHD and stroke case fatality risk for prevalent cases, and annual individualised non-CVD mortality risk for all.

For the health economics module, we used estimates for QALYs and the costs of CHD and stroke (Technical Appendix “Direct and indirect costs and productivity losses” in Supplementary Methods). Specifically, we calculated QALYs using disutility values based on the Japanese population norms of the EQ-5D-5L established by a previous study and recommended by Japanese guidelines for health technology assessment.^18^ The disutility values were reported by demographic factors and diseases, of which we used age groups, sex, diabetes, stroke, and CHD.^18^ For direct costs for CHD and stroke, we sourced Estimates of National Medical Care Expenditure for 2019.^19^ Indirect costs for productivity and informal care were derived from a 2017 study that reported the costs of illness for cancer, heart disease, and stroke in Japan.^20^

### Simulation scenarios for CVD risk factors

The scenarios were designed to quantify the contribution of changes in CVD risk factors since 2001, both singly and in combination, to changes in CHD and stroke burden over the same period. This was achieved by comparing the base-case scenario with counterfactual scenarios.

1) Base-case scenario—simulates individual-level life courses approximating the observed trends in CVD risk factors, CHD and stroke incidence and mortality, and all-cause mortality between 2001 and 2019. Note that SBP, LDL-c, and HbA1c were adjusted, respectively, for use of antihypertensives, cholesterol-lowering, and diabetes medications.
2) Combined scenario—simulates counterfactual individual-level life courses assuming that the exposure of the population to all the following risk factors by age and sex remained fixed at 2001 levels. Essentially, the combination of the following seven scenarios. Hence, this combined scenario cancels the observed changes of all modelled CVD risk factors.
3) Seven separate scenarios—Each scenario simulates counterfactual individual-level life courses assuming that the population's exposure to one of the modelled CVD risk factors (i.e., smoking status [categorical: current, past, never smokers; number of cigarettes for current smokers as a continuous variable], and the continuous variables: SBP [adjusted for antihypertensive medication use], LDL-c [adjusted for cholesterol-lowering medication use], HbA1c [adjusted for diabetes medication use], BMI, physical activity [times/day], and FV consumption [g/day]) by age and sex remains fixed at the 2001 level throughout the simulation period, while all other risk factors are similar to the base-case scenario. Hence, these separate scenarios cancel the observed changes of the selected risk factor. For example, the SBP scenario cancels the observed decline in SBP since 2001.

### Outcome measures as model outputs

The primary outcome was national CVD incidence (combined and separate CHD and stroke). Secondary outcomes included national CVD case-years, all-cause mortality, direct and indirect CVD costs, and QALYs, stratified by sex. These outcomes were presented as:

1) Disease outputs: CVD incidence and all-cause mortality.
2) Differences in the disease outputs between the base-case and counterfactual scenarios: cases prevented or postponed (CPPs), case-years prevented or postponed (CYPPs), and deaths prevented or postponed (DPPs). CPPs do not account for the duration of event postponement, whereas CYPPs do. For example, postponing a CHD event by 10 years counts as 1 CPP and 10 CYPPs. Negative CPPs and CYPPs indicate cases caused or accelerated by the modelled scenarios. Cumulative CPPs and CYPPs from 2001 to 2019 are reported unless otherwise stated.
3) Health economic outputs: QALYs and direct/indirect CVD costs.
4) Differences in QALYs and direct/indirect CVD costs between the base-case and counterfactual scenarios, summarised as net gained QALYs, and net saved direct and indirect CVD costs.

We did not discount costs or QALYs, as this study focuses on the past. All costs were converted to the 2021 Japanese yen, then to USD using the 2021 IMF rate (1000 JPY = 10·56 USD) via CCEMG–EPPI Centre Cost Converter (v. 1·7, updated January 2024).

### Uncertainty and sensitivity analysis

IMPACT_NCD-JPN_ uses a second-order Monte Carlo simulation (200 outer, 400,000 inner iterations) to propagate input uncertainty to outputs. Results are summarised using medians and 95% uncertainty intervals (UIs). Scenarios share common parameters, causing covariance among results; thus, overlapping UIs do not imply a lack of statistical significance (Technical Appendix “Uncertainty and probabilistic sensitivity analysis” in Supplementary Methods).

### Model calibration and validation

Annual incidence and mortality proportions for CHD, stroke, and other causes were calibrated to input trends (Technical Appendix “Validation and calibration” in Supplementary Methods). IMPACT_NCD-JPN_ was validated following existing guidelines,^21^ including: 1) face validity through author discussions and peer review of IMPACT_NCD_ framework of implementation, structure, and outputs; and 2) extensive internal validation comparing base-case outputs with input distributions for CVD risk factors, incidence, and mortality proportions (Supplementary Materials A & B in Supplementary Methods).

### Ethics approval

Ethical review was deemed unnecessary by the Research Ethics Committee of the National Cerebral and Cardiovascular Centre, in accordance with the Japanese Ethical Guidelines for Medical and Biological Research Involving Human Subjects. This was based on the use of publicly available online data and anonymised data from the Ministry of Health, Labour and Welfare, which contained no identifiable personal information.

### Role of the funding source

The funding sources of the present study had no role in the study design or conduct, data collection, data analysis, manuscript preparation, or review.

## Results

### Characteristics and validation results

Characteristics estimated in the disease and health economics module of IMPACT_NCD-JPN_ in the base-case scenario, aligned closely with real-world evidence, are shown in Table 1 (for CVD [CHD plus stroke] in Supplementary Table S1 in Supplementary Results). Detailed validation results showed that the simulation results of the base-case scenario successfully approximated the observed national trends in distributions of the seven CVD risk factors, the probability of developing CHD and stroke, and their probability of dying of these diseases or any other causes (Supplementary Materials A & B in Supplementary Methods).

**Table 1:** Characteristics (with 95% UI) in 2001 and 2019 in Japan, estimated in the disease and health economics module of IMPACT_NCD-JPN_ in the base-case scenario, aligned closely with real-world evidence.

Variables	Men		Women	
	2001	2019	2001	2019
**Population size (in thousand)**				
30–64 years old	30,371	28,735	30,513	28,268
65 and over years old	9618	15,521	13,253	20,232
Total	39,989	44,256	43,766	48,500
**CHD**				
Crude incidence (per 100,000)	340 (200–660)	420 (270–680)	190 (110–370)	300 (190–500)
Crude prevalence (per 100,000)	3100 (2600–3700)	4800 (3300–7300)	1300 (1100–1700)	2700 (1800–4400)
Crude mortality (per 100,000)	90 (55–140)	83 (47–170)	65 (41–100)	45 (24–92)
Direct costs (in USD)	3·3B (2·1B–5·2B)	5·2B (not applicable)	1·1B (0·6B–1·8B)	2·2B (not applicable)
Indirect costs (in USD)	29B (16B–43B)	22B (15B–29B)	3·8B (1·9B–7·1B)	2·6B (1·3B–4·2B)
Direct costs (in JPY)	310B (200B–490B)	490B (not applicable)	100B (61B–170B)	210B (not applicable)
Indirect costs (in JPY)	2700B (1500B–4100B)	2100B (1400B–2800B)	360B (180B–680B)	250B (130B–400B)
**Stroke**				
Crude incidence (per 100,000)	390 (240–630)	330 (230–500)	280 (200–420)	250 (180–360)
Crude prevalence (per 100,000)	4100 (3600–4500)	5200 (4100–7300)	2700 (2500–3000)	3600 (2900–4800)
Crude mortality (per 100,000)	150 (110–220)	100 (70–190)	140 (110–210)	85 (65–130)
Direct costs (in USD)	6·1B (4·7B–7·8B)	9·8B (not applicable)	5·6B (4·5B–7B)	9·4B (not applicable)
Indirect costs (in USD)	28B (20B–38B)	24B (20B–29B)	10B (7·8B–15B)	11B (10B–13B)
Direct costs (in JPY)	580B (450B–740B)	930B (not applicable)	530B (430B–670B)	890B (not applicable)
Indirect costs (in JPY)	2600B (1900B–3600B)	2300B (1900B–2700B)	960B (740B–1400B)	1100B (960B–1200B)

Abbreviations: UI, uncertainty interval; CHD, coronary heart disease; JPY, Japanese yen; USD, US dollars; QALYs, quality-adjusted life years.

Estimates of direct costs for 2019 were used as a reference and do not have any uncertainty.

### Trends in the modelled CVD risk factors

Supplementary Figure S1 in Supplementary Results illustrates the annual trends in CVD risk factors in the base-case scenario, reflecting the observed national trends and the seven separate counterfactual scenarios, where each of the CVD risk factors is assumed to remain at 2001 levels, as modelled by IMPACT_NCD-JPN_. For example, from 2001 to 2019, SBP (men/women) decreased from 140·1/135·3 to 133·4/128·1 mmHg in the base-case scenario, whereas in the SBP counterfactual scenario, it remained nearly unchanged, changing only from 140·1/135·3 to 139·2/134·5 mmHg. Each CVD risk factor was modelled based on age, sex, year, and, where applicable, selected relevant CVD risk factors among those included in the model (see the ‘Demographic module’, ‘Exposure module’, and ‘Clustering of risk factors’ subsections in ‘Epidemiological engine’ of the Technical Appendix in Supplementary Methods for details). Due to interdependencies and changes in population structure between 2001 and 2019, the descriptive statistics of modelled CVD risk factors in the counterfactual scenarios differed slightly from their 2001 values.

### Primary outcome

**Fig. 2:**
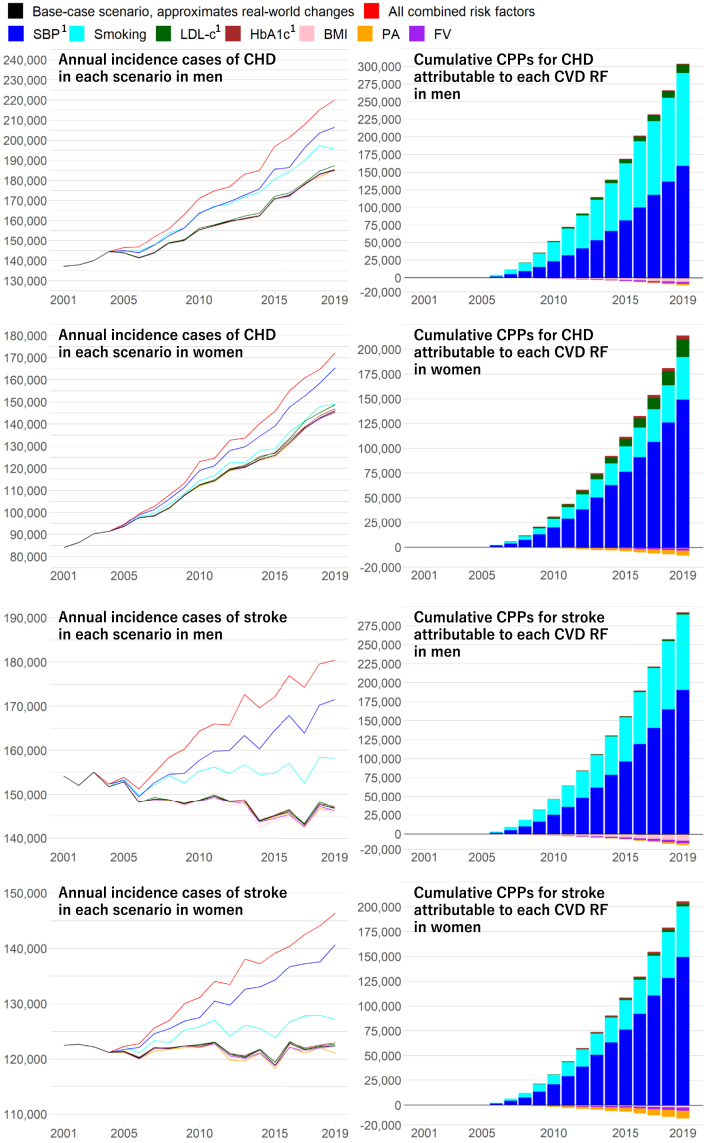
**Annual incidence of CHD and stroke, and their cumulative CPPs from 2001 to 2019 in Japan, under the base-case scenario and counterfactual scenarios where each modelled CVD risk factor, and all combined, are fixed at 2001 levels.** Abbreviations: CHD, coronary heart disease; CVD, cardiovascular disease; PA, physical activity; FV, fruit and vegetable consumption; BMI, body mass index; LDL-c, low-density lipoprotein cholesterol; SBP, systolic blood pressure; CPPs, cases prevented or postponed. ^1^SBP, LDL-c, and HbA1c were adjusted for medication use of antihypertensive, cholesterol-lowering, and diabetes treatments, respectively.

Fig. 2 illustrates the annual incidence of CHD and stroke, along with their cumulative CPPs, from 2001 to 2019 in Japan, for both the base-case and all counterfactual scenarios, based on the distributions of CVD risk factors presented in Fig. 1 and Supplementary Figure S1 in Supplementary Results. In the base-case scenario, closely aligned with real-world evidence, annual incidence cases of CHD and stroke are represented by black lines in Fig. 2. In 2001, IMAPCT_NCD-JPN_ estimated 137,000 (95% UI: 79,100–265,000) incident cases of CHD in men and 84,100 (48,500–162,000) in women, and 154,000 (95,800–252,000) incident stroke cases in men and 122,000 (85,600–185,000) in women (Fig. 2).

In the combined counterfactual scenario, with all seven CVD risk factors fixed at 2001 levels, the annual incidence of CHD and stroke cases is represented by the red lines in Fig. 2. Between the base-case scenario and the combined counterfactual scenario in 2019, respectively, CHD incidence was 185,000 (95% UI: 118,000–300,000) vs 220,000 (137,000–352,000) in men and 146,000 (90,000–243,000) vs 172,000 (106,000–292,000) in women; stroke incidence was 147,000 (102,000–221,000) vs 180,000 (124,000–270,000) in men and 122,000 (87,300–174,000) vs 146,000 (102,000–213,000) in women. The observed changes in all modelled CVD risk factors prevented or postponed CHD cases of 290,000 (140,000–510,000) in men and 210,000 (99,000–400,000) in women, stroke cases of 280,000 (150,000–460,000) in men and 190,000 (110,000–310,000) in women, which were cumulative CPPs between 2001 and 2019 (Table 2). The results for CVD (CHD plus stroke) are presented in Supplementary Table S2 and Figure S2 in Supplementary Results.

**Table 2:** Contribution (95% UI) of cumulative changes in all modelled CVD risk factors^a^ on national burden of CVD from 2001 to 2019 in Japan estimated by IMPACT_NCD-JPN_.

Cumulative values from 2001 to 2019	Men	Women
**CPPs**		
CHD	290,000 (140,000–510,000)	210,000 (99,000–400,000)
Stroke	280,000 (150,000–460,000)	190,000 (110,000–310,000)
**CYPPs**		
CHD	880,000 (290,000–1,600,000)	620,000 (280,000–1,200,000)
Stroke	790,000 (340,000–1,500,000)	570,000 (300,000–980,000)
**DPPs**	550,000 (460,000–680,000)	290,000 (220,000–390,000)
**Net gained QALYs**	1,600,000 (960,000–2,400,000)	1,300,000 (950,000–1,900,000)
**Net saved direct costs**		
CHD (in USD)	2·3B (1·3B–3·6B)	1·1B (0·8B–1·7B)
Stroke (in USD)	2·5B (1·1B–4·2B)	2·5B (1·4B–4·1B)
CHD (in JPY)	210B (120B–340B)	110B (72B–160B)
Stroke (in JPY)	230B (100B–400B)	230B (130B–380B)
**Net saved indirect costs**		
CHD (in USD)	86B (55B–130B)	11B (6·5B–21B)
Stroke (in USD)	47B (34B–76B)	13B (8B–21B)
CHD (in JPY)	8100B (5200B–13000B)	1100B (620B–1900B)
Stroke (in JPY)	4400B (3200B–7200B)	1300B (760B–2000B)

Abbreviations: UI, uncertainty interval; JPY, Japanese yen; USD, US dollars; CVD, cardiovascular disease; CHD, coronary heart disease; CPPs, cases prevented or postponed; CYPPs, case-years prevented or postponed; DPPs, deaths prevented or postponed; QALYs; quality-adjusted life years.

a All modelled CVD risk factors consisted of systolic blood pressure, smoking, physical activity, low-density lipoprotein cholesterol, HbA1c, fruit and vegetable consumption, and body mass index. Additionally, SBP, LDL-c, and HbA1c were adjusted for medication use of antihypertensive, cholesterol-lowering, and diabetes, respectively.

Fig. 2 also shows annual CHD and stroke incidence for each of the counterfactual scenarios of the seven CVD risk factors, with each of them fixed at 2001 levels. Furthermore, we calculated the cumulative CPPs for CHD and stroke attributed to each CVD risk factor (in Fig. 2) based on the results of the counterfactual scenarios for the combined and each of the seven CVD risk factors. These show that the cumulative CPPs for CHD and stroke were attributed mainly to SBP and smoking status trends and modestly to LDL-c and HbA1c trends; in contrast, the cumulative CPPs were partially offset by the BMI, physical activity, and FV consumption trends. Again, the distributions of CVD risk factors in 2001 and 2019 for the base-case and all counterfactual scenarios are shown in Fig. 1 and Supplementary Figure S1 in Supplementary Results. Note that trends in SBP, LDL-c, and HbA1c were adjusted for the use of antihypertensives, cholesterol-lowering, and diabetes medications, respectively. Their detailed results, including CVD (combined and separate CHD and stroke), are summarised in Supplementary Tables S3 and S4 in Supplementary Results.

### Secondary outcomes

**Fig. 3:**
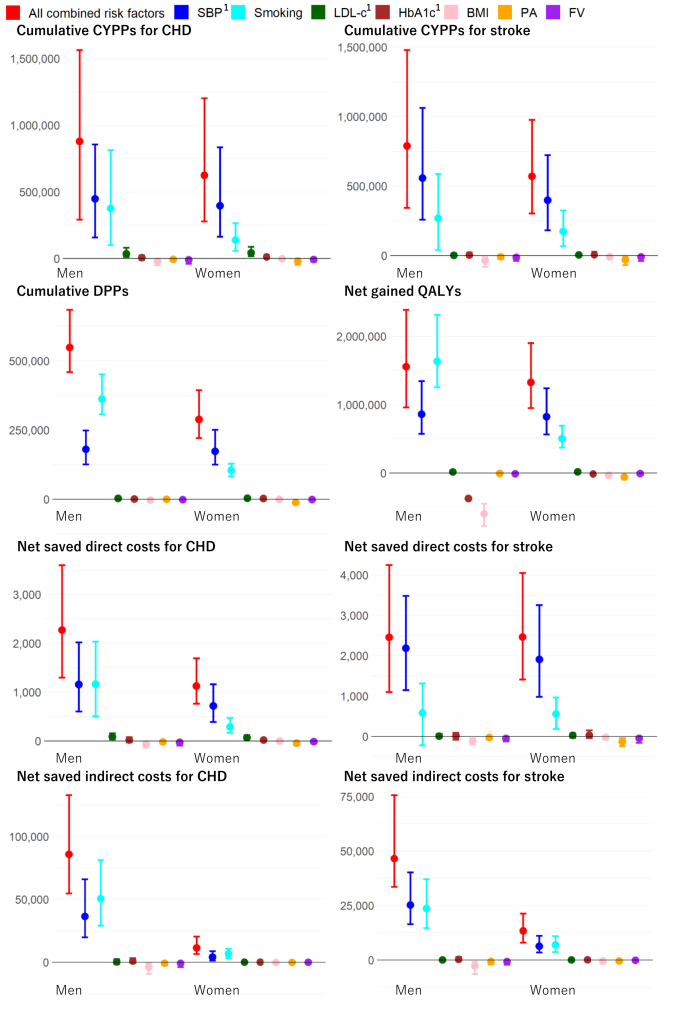
**Cumulative CYPPs for CHD and stroke, DPPs, net gained QALYs, net saved direct and indirect costs for CHD and stroke from 2001 to 2019 in Japan, assessed by comparing counterfactual scenarios where each modelled CVD risk factor, and all combined, are fixed at 2001 levels with the base-case scenario.** Abbreviations: CHD, coronary heart disease; CVD, cardiovascular disease; PA, physical activity; FV, fruit and vegetable consumption; BMI, body mass index; LDL-c, low-density lipoprotein cholesterol; SBP, systolic blood pressure; CYPPs, case-years prevented or postponed; DPPs, deaths prevented or postponed. Results are summarised using medians (points) and 95% uncertainty intervals (error bars). Since the scenarios share common parameters, the results are correlated; therefore, overlapping UIs do not necessarily indicate a lack of statistical significance. For details, please refer to Technical Appendix “Model outputs” in Supplementary Methods. ^1^SBP, LDL-c, and HbA1c were adjusted for medication use of antihypertensive, cholesterol-lowering, and diabetes treatments, respectively.

Fig. 3 illustrates the cumulative CYPPs for CHD and stroke, DPPs, net gained QALYs, and net saved direct and indirect costs for CHD and stroke from 2001 to 2019 in Japan, as assessed by comparing the counterfactual scenarios with the base-case scenario. In the combined counterfactual scenario, these results are represented by red dots and the 95% UI by red bars in Fig. 3 and Table 2. For example, the observed changes in all modelled CVD risk factors prevented or postponed all-cause deaths of 550,000 (460,000–680,000) in men and 290,000 (220,000–390,000) in women, which were cumulative DPP between 2001 and 2019 (Table 2). Those results for CVD (CHD plus stroke) are presented in Supplementary Table S2 in Supplementary Results.

Fig. 3 also presents the results for each of the counterfactual scenarios involving the seven CVD risk factors. The cumulative CYPPs for CHD and stroke, DPPs, net gained QALYs, and net saved direct and indirect costs for CHD and stroke from 2001 to 2019 in Japan were primarily attributed to trends in SBP and smoking status, with modest contributions from trends in LDL-c and HbA1c. However, HbA1c trends negatively contributed to QALYs, differing from other outcomes. In contrast, these cumulative benefits were partially offset by trends in BMI, physical activity, and FV consumption. Their detailed results, including CVD (combined and separate CHD and stroke), are summarised in Supplementary Tables S3–S6 in Supplementary Results. For example, increases in BMI, and decreases in physical activity and FV consumption, respectively, were estimated to produce increased net cumulative direct costs of CVD by 180M, 48M, and 73M USD for men, and 29M, 170M, and 63M USD for women (Supplementary Table S6 in Supplementary Results).

## Discussion

As the principal findings, the present IMPACT_NCD-JPN_ quantified that changes in the national distribution of the key CVD risk factors cumulatively prevented or postponed CHD and stroke cases at the national level in Japan between 2001 and 2019. These CPPs would result in preventing or postponing cumulative all-cause deaths, gaining net QALYs, and saving net direct/indirect costs of CHD and stroke. Decreases during the period in SBP and smoking prevalence mainly explained this reduction in the national burden of CVD; in contrast, increases in BMI and decreases in physical activity and FV consumption partially offset the reduction. Furthermore, similar patterns were observed in both CHD and stroke for men and women. However, changes in LDL-c distribution would be considered to influence the CHD burden more explicitly than the stroke burden. The contributions of changes in BMI and HbA1c distributions to CHD and stroke burden were more favourable in women than men.

In the present results, reduced SBP and improved smoking status significantly contributed to lowering Japan's CVD burden between 2001 and 2019. Between 1961 and 2016, mean SBP in Japan decreased by 10–20 mmHg across all age groups.^22^ likely due to early hypertension detection via health checkups, reduced salt intake, and better treatment.^22^ Smoking prevalence among those aged 25–94 declined from 49·3% to 33·1% (men) and 14·1% to 10·7% (women) between 2001 and 2016,^23^ partially driven by rising tobacco prices and socioeconomic factors.^23^^,^^24^ Tobacco price increases raised cessation prevalences by 7·0% (men) and 6·5% (women) from 2007–2010.^24^ Higher-educated and non-manual workers showed larger smoking declines from 2001–2016.^23^

However, further improvements in hypertension and smoking management are needed, especially among men and lower socioeconomic groups. Despite better hypertension treatment and control from 1980 to 2016, Japan's hypertension prevalence remains higher (22·5%, 23·0%, and 29·0% for women; 40·3%, 29·9%, and 34·1% for men in Japan, UK, and US in 2019), with lower treatment (51·2%, 47·9%, and 73·3% for women; 45·9%, 47·4%, and 66·3% for men) and lower control rates (30·3%, 29·2%, and 51·0% for women; 24·1%, 30·6%, and 44·8% for men).^22^^,^^25^ Japan's smoking prevalence is higher among men (33·4% vs 21·7% UK, 19·9% US), though lower among women (10·2% vs 18·1% UK, 15·3% US).^26^ Equity-focused tobacco control and better hypertension management can further reduce Japan's future CVD burden.

In relation to previous simulation studies in Japan, the primary contribution of national-level changes in SBP and smoking to national CHD deaths was similar between our present IMPACT_NCD-JPN_ model and the previous IMPACT_FIRST-JPN_ model. The previous model showed that the two-time point (i.e., non-cumulative) declines in the number of CHD deaths between 1980 and 2012 in Japan were attributed to decreases in SBP by 8·87 mmHg and in current smoking prevalence by 14·0%, contributing 24% (95% UI: 20–29%) and 11% (95% UI: 8–14%) to the overall CHD death reduction at the national level, respectively.^1^ IMPACT_NCD-JPN_ uses more data points, reports more outcomes, including costs and QALYs, and better quantifies uncertainty compared to IMPACT_FIRST-JPN_. Finally, a recent microsimulation model in Japan projected the future prevalence of dementia and frailty but not the CVD burden.^27^

Our analysis of costs and QALYs showed that, despite the estimated net cost savings and QALY gains overall, unfavourable trends in BMI, physical activity, and FV consumption incurred costs and loss of QALYs that can be considered the cost of failing to implement effective public health policies targeting these risk factors. Supplementary Tables S7 and S8 in Supplementary Results further stratify our estimates by age. The direct cost savings in the elderly aged ≥65 were more than in the younger aged between 30 and 64; in contrast, the indirect cost savings in the younger were more than in the elderly, highlighting the impact of informal care on the workforce.

The present results can have public health implications for Japan. Japan should maintain SBP, smoking, and LDL-c control policies and urgently address HbA1c, BMI, physical activity, and FV consumption to mitigate the anticipated increase in CVD burden due to its ageing population, with projections showing that 35% of the population will be over 65 years old by 2040.^11^^,^^28^ This can also be supported by previous studies showing that decreases in CVD deaths and all-cause deaths attributable to CVD risk factors, including SBP and smoking, have stagnated worldwide, including in Japan, especially from 2010 to 2019.^29^^,^^30^ Note that we do not assume that the same magnitude of effects which IMPACT_NCD-JPN_ estimated will occur in the future, especially as further reductions in SBP may yield diminishing returns. Therefore, we caution against directly extrapolating these results beyond the study period. Future studies could build on our findings by modelling alternative scenarios—such as maintaining current trends or implementing specific policy interventions—projected through to 2040, to estimate their potential impact on the CVD burden.

The present study has the following strengths. To our knowledge, IMPACT_NCD-JPN_ is the first validated microsimulation model to quantify changes in the national distribution of multiple CVD risk factors to the national burden of CVD, including CVD cases and case-years, all-cause mortality, CVD costs, and QALYs, in Japan. Additionally, our results in Japan provide useful suggestions to other countries which are becoming ‘super-aged’. As the most rapidly ageing country in the world, Japan would be most affected by population ageing and its impact on CVD.^11^^,^^28^

The present study has the following limitations and, like all modelling analyses, simplifies reality. First, our results depend on the accuracy of input data and parameters even though IMPACT_NCD-JPN_ used high-quality risk estimates from existing meta-analyses. Due to the absence of a national CHD and stroke registry or access to national electronic health records in Japan, we used GBD estimates, assuming they are a reliable source of age-, sex-, and year-specific incidence and prevalence data for CHD and stroke among the accessible data sources. The GBD incorporates multiple empirical studies, including Ministry surveys and regional population-based data. To address remaining uncertainty, we conducted probabilistic sensitivity analysis using GBD's credible intervals. Although national CVD morbidity data were unavailable, GBD estimates aligned with available local registries (Supplementary Material C in Supplementary Methods).^31^^,^^32^ As established national data on risk-factor coefficients or incidence for CHD and stroke subtypes are lacking, IMPACT_NCD-JPN_ models total CHD and total stroke only. Subtype incidence was estimated externally using published population-based rates applied to the 2019 population (Supplementary Table S9)^31^, ^32^, ^33^ and served as contextual description, not input for simulation. Second, the model included only seven key CVD risk factors, CHD, and stroke, excluding others (e.g., sodium intake, socioeconomic status, new medical treatments, race/ethnicity) and diseases like heart failure. These omissions are unlikely to introduce substantial bias. Race/ethnicity data were unavailable, reflecting Japan's norm of not collecting such data due to demographic homogeneity, limiting assessment of race/ethnicity group disparities. Further research will expand the model's scope. Third, pre-2000 exposure data for HbA1c, LDL-c, physical activity, and smoking required back-projection, but these projections aligned with observed trends (Supplementary Material B in Supplementary Methods). Fourth, the absence of a micro-costing approach may have underestimated the heterogeneity of CVD costs. Finally, in 2019, the NHNS switched from mercury sphygmomanometers to validated mercury-free devices. Although introduced under strict comparability standards, the protocol did not state whether the device's “mark” button—affecting accuracy—was used. A slight drop in mean SBP was seen in 2019, but similar fluctuations occurred before. As this change occurred only in the final year of a 19-year period and our regression-based approach (GAMLSS model) is robust to such variation, the impact on findings is likely minimal. See Supplementary Material D for details.

### Conclusions

The validated IMPACT_NCD-JPN_ microsimulation model showed that changes in the national distribution of seven CVD risk factors between 2001 and 2019 prevented or postponed CHD and stroke cases, all-cause deaths, and associated costs and increased QALYs in Japan. Reduced SBP and smoking cessation mainly drove these benefits, while stagnant LDL-c and HbA1c contributed modestly. However, rising BMI and declines in physical activity and FV consumption partially offset them.

## Contributors

SO, EK, YY, CK: study design; SO, KI: funding; SO, EK, YY, KI, HF, MI, KT, KN: data collection; SO, EK, YY, CK: data analysis; SO, EK, YY, AH, MOF, BC, KN, CK: data interpretation and writing; SO, EK, YY, KN, CK: had full access to all the data and attest to the completeness and accuracy of the data and data analyses; All authors: critical review of the manuscript, read and approved the final manuscript.

SO, EK, YY, CK, KN have accessed and verified the data.

SO, CK, KN were responsible for the decision to submit the manuscript.

## Data sharing statement

The dataset(s) will be anonymised before being shared. Access to the data and related documentation will be provided exclusively through a data-sharing agreement. Codes for developing IMPACT_NCD-JPN_ are available at https://github.com/ChristK/IMPACTncd_Japan.

## Declaration of interests

KI receives a grant from Nxera Pharma Japan and Idorsia Pharmaceuticals Japan. However, this grant was not used for the present study. Thus, this funding source had no role in the study design or conduct, data collection, data analysis, manuscript preparation, or review. The other authors report no conflicts of interest related to this paper.

## Appendix A Supplementary data

Supplementary File

Translated Abstract–Japanese
