# Supplementary material for: Quantifying the contributions of cardiovascular risk factors to cardiovascular disease trends in 21st century Japan: a microsimulation study
Source: Lancet Reg Health West Pac. 2025 Jul 8;60:101623. doi: 10.1016/j.lanwpc.2025.101623 (PMC12274934; doi:10.1016/j.lanwpc.2025.101623)
Supplement: Supplementary File [file mmc1.pdf]

Supplementary File

Contents

Supplementary Methods **3**
Technical Appendix **3**
1. Introduction 3
2. Epidemiological engine 6
   High-level description 6
   Demographic module 9
   Exposure module 10
      Clustering of risk factors 12
   Disease module 12
      Disease incidence 12
      Mortality 16
3. Population-attributable risk fraction (PARF) 16
4. Model outputs 16
   Uncertainty and probabilistic sensitivity analysis 16
5. Validation and calibration 17
6. Direct and indirect costs and productivity losses 18
7. Strengths and limitations of this modelling approach 18
8. References 20
A) Supplementary Material A: Disease Module Details and Validation **23**
   Model structure 23
   Coronary Heart Disease (CHD) 24
      Validation plots for CHD incidence and mortality 27
   Stroke (ischemic stroke and hemorrhagic stroke, including subarachnoid hemorrhage) 29
      Validation plots for stroke incidence and mortality 31
   Non-modelled mortality 33
      Validation plots for non-modelled mortality 35
B) Supplementary Material B: Exposure Modelling Details and Validation **36**
   Exposure modelling details 36
   Exposure validation plots 38

Body mass index 38
Smoking status 40
Number of cigarettes smoked (current smokers) 42
Physical Active days 44
Fruit and Vegetable intake 46
Medication use for diabetes 48
Medication use for hyperlipidemia 50
Medication use for hypertension 52
Systolic blood pressure 54
HbA1c 56
LDL-c 58
C) Supplementary Material C: Incidence rate comparisons between GBD and Japanese local registries 60
D) Supplementary Material D: Consideration of the transition from standard mercury sphygmomanometers to mercury-free devices in the 2019 National Health and Nutrition Survey 63

Supplementary Results 65
Supplementary Figures 65
Supplementary Tables 68

Supplementary Methods

Technical Appendix

NOTE: The model architecture description of the $\text{IMPACT}_{\text{NCD-JPN}}$ in this document is based on an earlier technical appendix of $\text{IMPACT}_{\text{NCD}}$ England published by the University of Liverpool. Thus, some of the text is similar to that in the earlier document.

1. Introduction

$\text{IMPACT}_{\text{NCD}}$ is an open-source microsimulation modelling framework for public health policy evaluation, planning, and decision-making focused on the prevention of non-communicable diseases (NCD). The present paper aimed to evaluate the effect of observed changes in cardiovascular disease (CVD) risk factors on CVD mortality, prevalence, incidence, medical costs, and Quality Adjusted Life Years (QALYs) between 2001 and 2019. The epidemiological engine of the modelling framework translates changes in the trends of disease risk factors into changes in disease incidence, case fatality, and subsequent disease prevalence. The policy layer of the framework complements the epidemiological engine and translates policy changes (hypothetical or real) into changes in the trends of disease risk factors.

$\text{IMPACT}_{\text{NCD}}$ Japan ($\text{IMPACT}_{\text{NCD-JPN}}$) is presented in the present paper for the first time, while $\text{IMPACT}_{\text{NCD}}$ has been used extensively to model primary prevention policies nationally in England, Brazil, Germany, and the United States (US) and locally in Liverpool, a city in the northwest of England.[1–10] At the core of the $\text{IMPACT}_{\text{NCD-JPN}}$ model is an epidemiological engine that includes age, sex, smoking, fruit and vegetable consumption, physical activity, body mass index (BMI), systolic blood pressure (SBP), haemoglobin A1c (HbA1c), and low-density lipoprotein cholesterol (LDL-c), as risk factors. The diseases modelled from risk factor trends include coronary heart disease (CHD) and stroke. The policy layer is dedicated to modelling either a specific public health policy formulation (e.g., freezing the trends of risk factors at a time point, like in the present paper) or a hypothetical scenario and consists of a mathematical/statistical model of the policy.

This iteration of $\text{IMPACT}_{\text{NCD-JPN}}$ uses data as follows. To inform trends in risk factors, the model used risk factor levels observed in the National Health and Nutrition Survey (NHNS) in Japan.[11,12] To inform trends in disease-specific mortality, the model used observed mortality of CHD and stroke from the Vital Statistics in Japan provided by the Ministry of Health, Labour and Welfare,[13] and projected mortality of CHD and stroke based on our Bayesian age-period-cohort models that we previously reported elsewhere.[14] To inform disease incidence and prevalence, the model used the prevalence and incidence of CHD and stroke estimated by the Global Burden of Diseases, Injuries, and Risk Factors Study (GBD) 2021 results version.[15,16] To inform estimates of the population size and structure and to calibrate mortality, the model used observed population size from the Population Census and Population Estimates provided by the Statistics Bureau, Ministry of Internal Affairs and Communications,[17] and projected population size estimated by the National Institute of Population and Social

Security Research in 2023.[18]

The $IMPACT_{NCD}$ framework is modular and can grow in several directions, with contributions from us and others. For example, it allows third parties to develop policy layers independently and hook them to the epidemiological engine. The open-source license (GPLv3) ensures transparency and accountability while promoting collaborative work throughout development and deployment. The source code of the current implementation is available at https://github.com/ChristK/IMPACTncd_Japan.

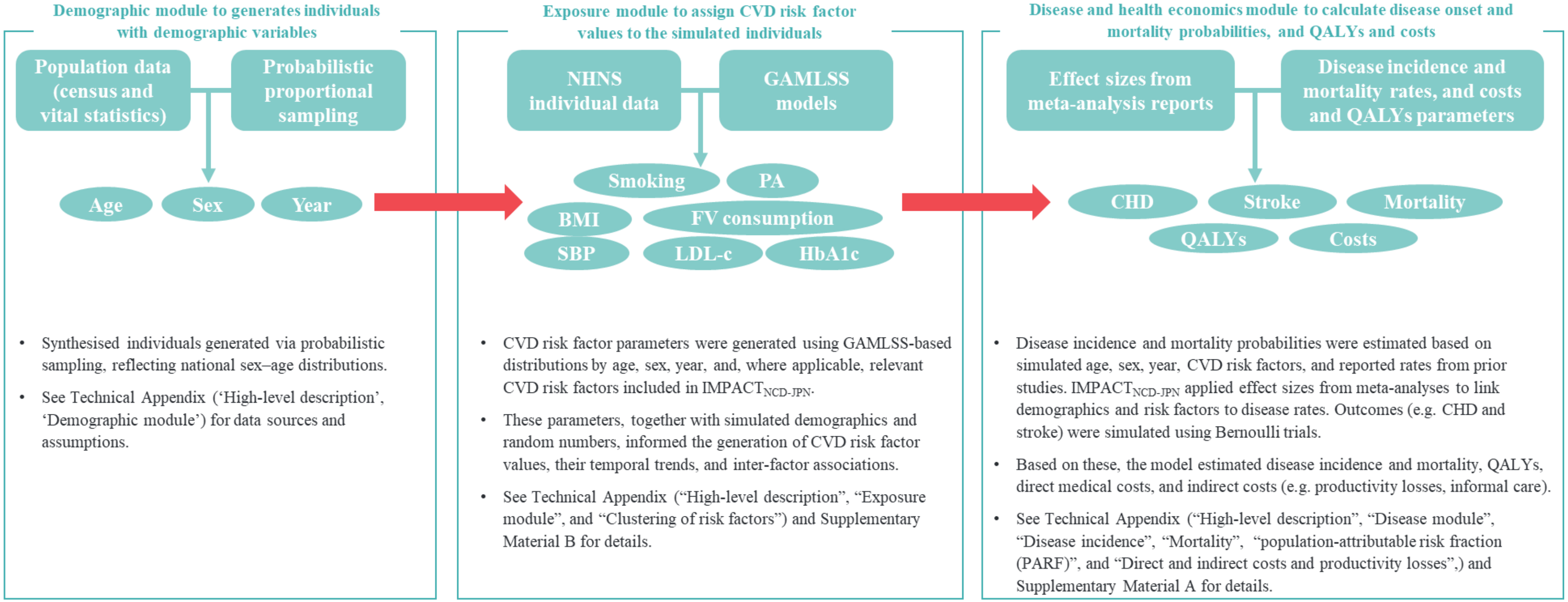

Uncertainty and Model Validation

- To address input uncertainty, IMPACT$_{NCD-JPN}$ used second-order Monte Carlo simulation; results are presented as medians with 95% uncertainty intervals.
- Validation involved expert review of the model framework and outputs (face validity), and internal comparisons of modelled outputs with input distributions for CVD risk factors, incidence, and mortality..
- For details, see the Technical Appendix ("Uncertainty and probabilistic sensitivity analysis", "Validation and calibration") and validation plots in Supplementary Materials A & B.

Figure-1-1. A brief summary of IMPACT$_{NCD-JPN}$ model and references to the Technical Appendix and Supplementary Materials in Supplementary Methods.

Abbreviations: CVD, cardiovascular disease; PA, physical activity; FV, fruit and vegetable; BMI, body mass index; SBP, systolic blood pressure; LDL-c, low-density lipoprotein cholesterol; HbA1c; GAMLSS, Generalized Additive Models for Location, Scale and Shape; QALYs, Quality-adjusted life years; CHD, coronary heart disease; NHNS, National Health and Nutrition Survery.

2. Epidemiological engine

High-level description

The epidemiological engine of $\text{IMPACT}_{\text{NCD-JPN}}$ is a discrete-time, dynamic, stochastic microsimulation consisting of three modules: the Demographic, Exposure, and Disease modules.

Within the $\text{IMPACT}_{\text{NCD-JPN}}$ epidemiological engine, each unit is a synthetic individual (simulant) represented by a record containing a unique identifier and a set of associated attributes. The microsimulation then projects the life course of each synthetic individual.

Each synthetic individual's attributes include demographic characteristics, exposures to risk factors, acquired diseases, and, if relevant, the cause of death.

Specific attributes include:

1. Age and sex as demographic exposures.
2. Smoking status (current smoker/ ex-smoker/ never-smoker), smoking intensity, fruit and vegetable consumption, and physical activity as behavioural risk exposure variables.
3. BMI, SBP, LDL-c, and HbA1c as biological risk exposures.
4. The risk for the two diseases is modelled explicitly: CHD and stroke. The risk for these conditions is modelled from relevant exposures to demographic, behavioural, and biological risk factor attributes, as well as certain prevalent conditions. For instance, the risk of CHD depends on age, sex, smoking, fruit and vegetable consumption, physical activity, BMI, SBP, LDL-c, and HbA1c. The Disease module section summarises the modelling approach, and Supplementary Material A: Disease Module Details give more detail on the modelling and data sources for each condition. Based on their risk of developing any of these conditions, simulants may develop some conditions during their life course.
5. Mortality from CHD, stroke, or any other cause is recorded if it occurs.

All these attributes are updated in discrete annual steps according to a set of stochastic rules. We structured these rules based on well-established epidemiological principles. Specifically, behavioural risk exposures are conditional on demographic exposures; biological risk exposures are conditional on behavioural and demographic exposures, and diseases are conditional on biological, behavioural, and demographic exposures. Finally, mortality is conditional based on demographic, behavioural, biological, and disease exposures.

The life course of synthetic individuals is simulated as many times as the number of scenarios to be modelled, using the same random numbers for all policy scenarios to reduce stochastic noise. One of the scenarios is always the 'base-case' scenario with which all remaining counterfactual or policy scenarios are compared. In this report, the base-case scenario is that of continuing trends in risk factors. Comparing the disease outcomes from the life courses under the base-case scenario versus the counterfactual or policy scenarios generates the health impact of the counterfactual or the policy scenarios. The output of the epidemiological engine is a dataset

containing the adult life course of the simulated synthetic individuals, with all the attributes mentioned above recorded annually for every scenario. From this dataset of life course trajectories, mortality, disease incidence and prevalence can be calculated annually.

As mentioned above, the epidemiological engine of $IMPACT_{NCD-JPN}$ consists of three modules: the demographic module, the exposure module, and the disease module. We will describe these three modules in the following paragraphs. Table 2-1 summarizes the key assumptions and limitations of the $IMPACT_{NCD-JPN}$ microsimulation model.

Table 2-1 IMPACT$_{NCD-JPN}$ key assumptions and limitations

**Model component**	**Key assumptions**
Demographic module	Migration is not modelled explicitly in the model. However, the model outputs are calibrated to official population reports and the official population projections in Japan provided by the Statistics Bureau, Ministry of Internal Affairs and Communications, and estimated by the National Institute of Population and Social Security Research, which consider migration.
	Social mobility is not considered.
Exposure module	We assume that the surveys used are truly representative of the population. IMPACT NCD Japan used the National Health Nutrition Survey (NHNS), an annual survey conducted by the Ministry of Health, Labour and Welfare of Japan. The NHNS uses a random sampling method to collect nationally representative data on the health, nutrition, and lifestyles of people in Japan. Results of the NHNS are widely used when developing and evaluating health promotions.
	On average, simulants remain in the same exposure quantile throughout their life (see Exposure module section).
	The linear correlations in exposure quantiles remain constant over time (i.e. the clustering of exposures in some subpopulations) (see Exposure module section).
	We assume that trends in risk factor exposures continue and follow log-linear trends.
Disease module	The incidence and prevalence of CHD and stroke were obtained from estimates of "ischemic heart disease" (ICD-10 codes I20–I25) and "stroke" (ICD-10 codes I60–I69), respectively, as reported in the Global Burden of Diseases, Injuries, and Risk Factors Study (GBD) 2021 results version.[15,16]<br><br>Mortality data for CHD and stroke were derived from the Japanese Vital Statistics published by the Ministry of Health, Labour and Welfare.[13] CHD mortality included deaths coded under "acute myocardial infarction" and "other ischemic heart diseases" (ICD-10 codes I20–I22, I24, and I25). Although ICD-10 code I23 (certain current complications following acute myocardial infarction) is excluded from Japan's official mortality tabulations, deaths

	originally certified as I23 are routinely reclassified to I21 or I22 (acute myocardial infarction or subsequent myocardial infarction) as the underlying cause of death, in accordance with coding guidelines. This classification practice, based on the ICD-10 (2013 revision), ensures no substantial loss of mortality data due to this exclusion. Stroke mortality was derived from deaths coded under "stroke" (ICD-10 codes I60–I69).
	We assume multiplicative risk effects (see Disease incidence section).
	We assume log-linear exposure-response for the continuous risk factors.
	We assume that the effects of the risk factors on incidence and case fatality are equal (see **Mortality** section).
	We assume a mean lag time between exposure and outcome of about 4-5 years for most exposure/outcome pairs (see Table 2-3 and Disease incidence section).
	We assume 100% risk reversibility for all exposures except smoking, for which the excess risk of ex-smokers is considered.
	We assume that the Vital Statistics data for Japan used to model disease trends over time represents Japan's adult population.
Continuing risk factor trends scenario (baseline)	We assume that trends in disease incidence continue to follow log-linear trends (see below).

Demographic module

The first year of every simulation in $IMPACT_{NCD\text{-}JPN}$ is 2001 in the present paper, so we could use the overlapping period 2001-19 (i.e., both observed values and the model estimates were available) to calibrate and internally validate the model.

For each simulation, the algorithm in the module:

1. Draw 400,000 synthetic individuals, aged 30 to 99, from the joint age-sex distribution. This is a default value that can be modified by the user. The joint age-sex distribution for 2001 is informed by the Statistics Bureau, Ministry of Internal Affairs and Communications Statistics Bureau, Ministry of Internal Affairs and Communications
2. We use this in the exposure module, as we describe in the next section.
3.

So far, the algorithm has created a synthetic population that is a snapshot of the population of Japan in 2001.

The following steps of the algorithm create backward and forward projections of the synthetic population that are essential to model exposure time trends and time lags between exposures and diseases.
The synthetic population's backward projection goes back to 1991, as the maximum time lag we allow in the model is ten years. As everyone alive and older than 30 in 2001 was alive in 1991, the algorithm simply creates the back projections by appropriately reducing the age of the synthetic individuals while keeping all other variables constant.

Similarly, for the forward projections, we project until 2019, and the algorithm increases the age of the synthetic individuals while keeping all other variables constant. For forward projections, mortality needs to be considered. We describe mortality with the disease module as disease-specific mortality is closely related to disease prevalence. $\text{IMPACT}_{\text{NCD-JPN}}$ follows an open cohort approach. Every simulated year from 2001 onwards, a new cohort of 30-year-old synthetic individuals enters the model. The same sources inform the cohort size and the joint age-sex distribution we described above. For example, in 2002, the new 30-year-old cohort will be informed by the population size and the joint age-sex distribution of those who were 29 years old in 2001. The approach may be crude; however, the final model outputs are directly standardised to population size reported by the Statistics Bureau, Ministry of Internal Affairs and Communications, and population projection estimated by the National Institute of Population and Social Security Research.

Exposure module

This module simulates the adult life course exposures of synthetic individuals based on the NHNS series between 2001 and 2019.[11] We followed the same general principles for all simulated exposures. First, we fit an appropriate statistical model to the NHNS data with the exposure of interest as the dependent variable and some functions of the year, age, sex, and other exposures of interest as independent variables. Then, we use the statistical model to predict the exposure level of every synthetic individual in the simulation based on their demographic characteristics estimated from the demographic module.

Including year as an independent variable in our exposure model allows us to extract the trends from the NHNS series and project them into the future. Furthermore, it allows us to make backward projections of exposures when we simulate time lags. For example, for a synthetic male aged 40 in 2001, we can estimate his SBP in 1991 when he was 30 and in 2011 when he would be 50 (Figure-2-1). To avoid excessively fast changes in exposure trends and to reflect our belief that decay and growth in natural phenomena are rarely linear, we included the natural logarithm of years in the statistical models assuming logarithmic trends.

We used logit ordinal regression to model exposures that were recorded as ordinal categorical variables. We used generalized additive models for location, scale, and shape (GAMLSS) for all other exposures.[19,20] These are flexible statistical models that can make all parameters of an assumed distribution for the dependent variable conditional to some function of the independent variables. For example, GAMLSS can model the mean and the standard deviation of a normally distributed dependent variable conditional on the predictors, while a linear

regression only models the mean.

Table **B-1** summarizes our modelling approach for all the exposures in the model.

The approach described above provides us with equations to estimate the distribution of the exposure to a risk factor for a given time and the demographic characteristics of a synthetic individual. When the synthetic individual enters the simulation, a vector of random numbers between 0 and 1 and of size equal to the number of the modelled exposures is allocated to the synthetic individual. Each one of the numbers represents the quantile of the relevant exposure distribution. The principle is that synthetic individuals retain their quantiles throughout their life course (this is known as the rank stability assumption).[21] For example, in 2001, a 40-year-old male synthetic individual with an SBP of 120 mmHg had an SBP quantile of 0.52. Ten years later, the same synthetic individual retained their quantile score for SBP. However, their SBP is now estimated to be 137.6 mmHg because the SBP distribution has changed to reflect the SBP of 50-year-old men in 2011 (Figure-2-1). In $\text{IMPACT}_{\text{NCD-JPN}}$, we allow the quantile of the synthetic individuals to fluctuate every year using a random walk to relax the rank stability assumption. However, on average, the large majority of synthetic individuals will have minimal changes to their exposure quantile throughout their lives.

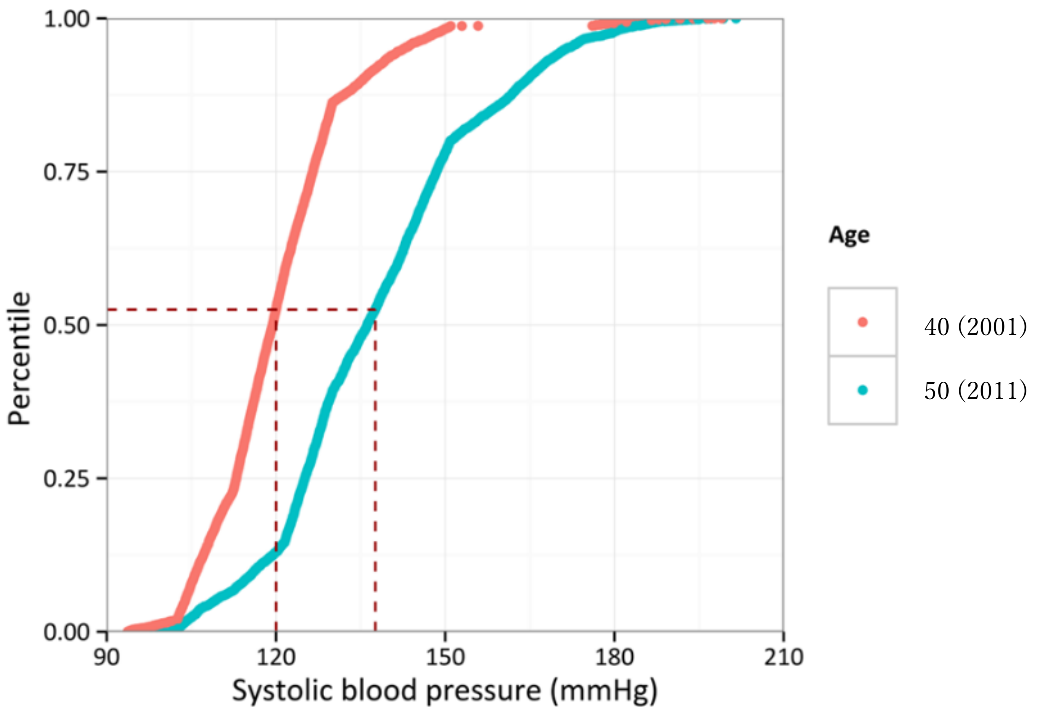

Figure-2-1 – Example of risk factor assignment for the synthetic population.
Plot of the systolic blood pressure quantiles of a male synthetic individual living for ages 40 in 2001; we can estimate his SBP in 1991 when he was 30, and in 2011 when he would be 50.

Clustering of risk factors

Finally, exposures in individuals are correlated. For example, people with a high BMI may also have high LDL cholesterol and hypertension. Some of these correlations reflect strong and well-established causal mechanisms, but the cross-sectional design of NHNS may bias these correlations. The method we described above captures some of these correlations by including exposures as independent variables in the statistical models for estimating exposures. For example, we included BMI as a predictor for SBP. Going a step further, we model the full correlation structure in NHNS using the following approach:

1. We used the exposure models to impute missing variables in NHNS.
2. We used the quantile function of the distribution estimated by the exposure models to convert exposures in NHNS to quantiles. Because the distributions were conditional on the independent variables used in each model, the quantiles are adjusted for these variables (i.e., age, sex, BMI, etc.).
3. We estimated the linear correlation matrix of the quantiles of the exposures of interest in NHNS using Pearson's correlation.
4. We used the linear correlation matrix from #3 to generate streams of uniform random numbers between 0 and 1 with a correlation structure similar to the one observed in NHNS.[22]
5. We used the correlated streams of random numbers from #4 as the exposure quantiles for the synthetic individuals.

For simplicity, we assumed that the correlation structure of the exposure quantiles remains constant over time.

Disease module

The previous two modules, demographics and exposure, generate a dynamic, close-to-reality synthetic population composed of each synthetic individual's adult life course exposures. The disease module then translates these exposures to disease incidence using a population-attributable risk fraction approach (PARF).[23] We will first describe how the model simulates disease incidence and then mortality.

We have modelled conditions in several different ways depending on the disease and the currently accepted causal associations between risk factors and diseases. The included risk factors and the modelled relationships between risk factors and disease incidence are those where sufficient, good-quality data on relative risks were available and where there is sufficient evidence of a causal relationship between a risk factor and the incidence of disease. Relative risks were obtained from published systematic reviews and meta-analyses supplemented with systematic literature searches. Supplementary Material A: Disease Module Details include a summary of all data sources. Table 2-2 summarizes the modelling approach for each condition, and further detail is given in Supplementary Material A: Disease Module Details.

Disease incidence

To estimate the individualised annual probability of a synthetic individual developing a specific disease

conditional on their cumulative risk exposures, we used the following 3-step approach.

Step 1. The incidence proportion attributable to each modelled risk factor by age and sex is estimated, assuming a specific time lag between exposure and disease. The relationships between exposures and disease incidence included in the model are outlined in Table 2-2. The time lags in the model vary stochastically between 1 and 10 years following a shifted binomial distribution. We set the mean lag time for each pair of risk exposure and disease combination according to the best possible empirical data based on the observation period of cohort studies and time to risk reversal in randomized clinical trials (see Table 2-3 and Supplementary Material A: Disease Module Details). For example, the mean lag time between SBP and an increase in CHD risk is 4 years. That means that a change in SBP will take 4 years on average to be translated to a change in CHD risk.

Step 2. The portion of the disease incidence attributable to all the modelled risk factors is estimated and subtracted from the total incidence for 2001, assuming multiplicative risks. By multiplicative risk, we mean, for example, that if the relative risk (RR) of obesity on CHD is 2 and the RR of smoking on CHD is 3, then the RR of an obese smoker is assumed to be $2 * 3 = 6$.[1]

Table 2-2: Overview of how individual conditions are modelled

Condition	Modelled condition	Recovery and recurrence	Causal relationships
			Risk factors
Stroke	Stroke including ischemic stroke and hemorrhagic stroke including subarachnoid hemorrhage	No recovery	Y
Coronary heart disease	Coronary heart disease	No recovery	Y

Step 3. The probability of developing the disease is estimated for each individual in the synthetic population, and the results are used in an independent Bernoulli trial to select those who finally develop the disease.

Table 2-3 Causal relationships included in the model between risk factors and disease incidence; the number corresponds to the mean time lag (in years) between exposure and outcome.

---

[1] An alternative approach would be to assume additive risks. Under this assumption and using the previous example, the RR of an obese smoker would be $2 + 3 - 1 = 4$. This multiplicative risk assumption is commonly used for epidemiological modelling.[24–27]

	Physical activity	BMI	Fruit and vegetable consumption	SBP	Smoking	LDL-c	HbA1c
CHD	4	4	4	4	4	4	4
Stroke	4	3	4	4	4	4	4
Mortality from non-modelled diseases	5			5	5		

BMI = body mass index; SBP = systolic blood pressure; HbA1c = haemoglobin A1c; LDL-c = low-density lipoprotein cholesterol.

The implementation of the above method is described in more detail using CHD as an example. The same process is used for all modelled diseases.

Step A

The population-attributable risk fraction (PARF) is an epidemiological measure that estimates the proportion of the disease attributable to an associated risk factor. It depends on the relative risk associated with the risk factor and the prevalence of the risk factor in the population. In a microsimulation context where exposures to risk factors are known at the individual level and assuming multiplicative risk factors, PARF can be estimated using the formula:

$$PARF = 1 - \frac{n}{\sum_{i=1}^{n}(RR_{i1} * RR_{i2} * \ldots * RR_{ik})}$$

where n is the number of synthetic individuals in the population, and $RR_{i1\ldots ik}$ are the relative risks of the risk factors associated with CHD for each individual i. We calculated PARF based on the above formula stratified by age and sex. Consistent with findings from the respective meta-analyses used for $\text{IMPACT}_{\text{NCD-JPN}}$ (Supplementary materials A Table A-2), SBP below 112.5 mmHg on average (derived randomly from a uniform distribution between 110 and 115), LDL-c below 85 mg/dL on average (derived randomly from a uniform distribution between 70 and 100), and BMI below 22 kg/m^2 on average (derived randomly from a uniform distribution between 21 and 23), HbA1c below 6.5% on average (derived randomly from a uniform distribution between 6 and 7) were considered to have a relative risk of 1.[28] Similarly, consumption of four on average (derived randomly from three, four, or five portions) or more portions per day of fruit and vegetables was considered to have a relative risk of 1. Five or more active days (more than 30 minutes of moderate to vigorous activity) per week for CHD, one or more days per week for stroke, and two or more days per week for non-modelled cause mortality were also considered to have a relative risk of 1. All the relative risks and the reference levels were taken from published meta-analyses and empirical studies (for references, see

Supplementary Material A: Disease Module Details where we describe each disease).

Step B

The incidence of CHD not attributable to the modelled risk factors can be estimated by the formula:

$$I_{Theoretical\ minimum} = I_{Observed} * (1 - PARF)$$

Where $I_{Observed}$ is the CHD incidence and $PARF$ is from Step A. $I_{Theoretical\ minimum}$ represents CHD incidence if all the modelled risk factors were at optimal levels. The theoretical minimum incidence is calculated by age and sex only in the initial year of the simulation, and it is assumed to be stable after that.

Step C

Assuming that $I_{Theoretical\ minimum}$ is the annual baseline probability of a synthetic individual to develop CHD for a given age and sex due to risk factors not included in the model (i.e., genetics, air pollution, dietary exposures beyond fruit and vegetables, etc.), the individualised annual probability of developing CHD, $\mathbb{P}(\text{CHD} \mid \text{age, sex, exposures})$, given their risk factors were estimated by the formula:

$$\mathbb{P}(CHD \mid age, sex, exposures) = I_{Theoretical\ minimum} * RR_{i1} * RR_{i2} * RR_{i3} * \ldots * RR_{ik}$$

Where $RR_{i1\ \ldots\ ik}$ are the relative risks that are related to the specific risk exposures of the synthetic individual, the same as in step A.

Estimating the observed incidence probability $\boldsymbol{I_{Observed}}$

For the observed incidence probability, $I_{Observed}$ we used the reported incidence from the GBD 2021 results version for the years 2001 to 2019 by 5-year age group and sex.[15,16] We used the reported credible intervals from GBD to fit beta distributions for each disease/year/age group/sex combination. The GBD is the most comprehensive worldwide observational epidemiological study to date and estimated several epidemiological indices, including incidence and prevalence from 1990 to 2021 for 371 diseases and injuries in 204 countries and territories, using a standardised, replicable approach, as well as a comprehensive update on fertility and migration.

Initial prevalence

For the initial simulation year, some synthetic individuals must be allocated as prevalent cases for each modelled disease. We followed a similar procedure as with the incidence above. We obtained point estimates and credible intervals and fitted beta distributions for each disease/year/age group/sex combination.

Mortality

All synthetic individuals are exposed to the risk of dying from any of their acquired modelled conditions or any other non-modelled cause. We treat the latter as a condition that everyone is a prevalent case of. That allows us to treat it like any other condition in the model. We used disease-specific mortality rates from CHD, stroke, and any other cause to calibrate the case fatality rates by year, age, and sex in the model in order to closely track disease mortality. We applied the same PARF approach described above for incidence to the case fatality. For instance, if the RR of smoking on CHD is 3, we assume that smokers have 3 times the risk of never-smokers developing CHD, and when they do, they have 3 times the risk of never-smoking CHD cases to die from CHD. Therefore, we allow exposures to risk factors to influence the mortality probability of the simulants.

Additionally, using a similar approach to the one we used to model disease incidence, we allowed synthetic individuals with SBP higher than 140 mmHg, active smokers, and those with one or less active day per week to experience higher non-modelled cause mortality rates.[29]

3. Population-attributable risk fraction (PARF)

The $\text{IMPACT}_{\text{NCD-JPN}}$ model allows for changes in the exposures of synthetic individuals based on defined 'what if' scenarios. The change in exposures allows us to calculate the individual risk factor effects on trends in modelled incidence. We can then compare the disease-related events between the counterfactual life courses.

4. Model outputs

The population in the model for this report was set for Japan. The model has a time horizon of 19 years, from 2001 to 2019. The simulation begins in 2001. The model outputs produce adult life-course trajectories for each simulant. From these, annual summary measures such as incidence, prevalence, and mortality rates are calculated. These measures can then be compared across time and between scenarios to estimate the effects and equity of different scenarios. All outcome measures can be standardised to the 2015 Japan Standard Population or not, and stratification is possible by year, sex, and age group, allowing examination of how effects differ across sub-populations.

Crucially, the model is a dynamic, open-cohort microsimulation model. That means it is trying to estimate the actual impact of the scenarios within a dynamic population where people are born, age, change their risk factors, and die. Thus, our model's detailed modelling of population dynamics is different from that of many economic models, which are often closed-cohort models. These models follow the same population cohort over time and often have a lifetime horizon.

Uncertainty and probabilistic sensitivity analysis

$\text{IMPACT}_{\text{NCD-JPN}}$ implements a 2nd order Monte Carlo approach to estimate uncertainty intervals (UI) for each

scenario.[30,31] For each iteration, a different set of input parameters is used by sampling from the respective distributions of input parameters. We assumed log-normal distributions for relative risks and hazard ratios, normal distributions for coefficients of linear regression equations, and beta distributions for estimates of incidence, prevalence, and case fatality rates. Specifically, for relative risks and hazard ratios, the distributions were bounded above 1 when the mean was above 1 and vice versa.

$\text{IMPACT}_{\text{NCD-JPN}}$ allows stochastic uncertainty, parameter uncertainty, individual heterogeneity, and, to some extent, structural uncertainty to be propagated in the reported UI. In $\text{IMPACT}_{\text{NCD-JPN}}$, we minimise stochastic uncertainty by using the same random numbers for all scenarios when appropriate. The following example illustrates the different types of uncertainty considered in the model. Let us assume that the annual risk for CHD is 5%. Suppose we apply this risk to all individuals and randomly draw from a Bernoulli distribution with p = 5% to select those who will manifest CHD. In that case, we only consider stochastic uncertainty. Our model minimises stochastic uncertainty when we compare different policy scenarios by using the same random numbers for all scenarios, where appropriate. If we allow the annual risk for CHD to be conditional on individual characteristics (i.e., age, sex, exposure to risk factors), then individual heterogeneity is considered. Finally, when the uncertainty of the relative risks due to sampling errors is considered in estimating the annual risk for CHD, the parameter uncertainty is considered. From these three types of uncertainty, only the parameter uncertainty could be reduced by better studies in the future.

The structure of the model is grounded in fundamental epidemiological ideas and well-established causal pathways on which exposures are causally related to the specific NCDs that are explicitly modelled. Therefore, we considered this type of uncertainty relatively small and did not study it in detail, with one exception: the discrete-time bias that arises from the fact that time in $\text{IMPACT}_{\text{NCD-JPN}}$ is not continuous. A synthetic individual within the model may die of multiple causes within one year; however, the simulation's discrete-time nature does not allow for identifying the cause that 'killed' the simulant first. Every time this happens to a simulant, we randomly select a cause of death from the list of all the terminal events that occurred for the simulant that year. Hence, we propagate discrete-time uncertainty to the output.

5. Validation and calibration

As we described in the disease module, trends in disease incidence are decomposed based on the trends of causally linked disease risk factors. The process leaves a residual proportion of annual incidence change that cannot be explained by the modelled risk factors, either because the residual is driven by risk factors that we do not explicitly model, like alcohol, or because the modelling of a risk factor is incomplete, e.g., we do not model the cumulative risk of smoking. To account for this, we calibrate the modelled disease incidence to the observed one for years between 2001 and 2019, assuming that the residual has a log-linear trend.

We validated the $\text{IMPACT}_{\text{NCD-JPN}}$ epidemiological engine using internal validation plotting the modelled exposures' prevalence and disease incidence against the observed exposures' prevalence and disease incidence in

NHNS, respectively. Mortality in the model is calibrated to mortality projections as described above (**Mortality**). We present the relevant validation plots for modelled exposures in Supplementary Material B: Exposure Modelling Details and Validation stratified by sex, year, and age group. Validation plots for CHD and stroke incidence and mortality of individual conditions are shown in Supplementary Material A: Disease Module Details. Additionally, we have produced and inspected plots for multiple combinations of stratification levels that are available on request. Overall, the plots suggest that $IMPACT_{NCD\text{-}JPN}$ captures exposure trends and translates them to disease incidence and mortality reasonably well for the purpose of this project.

6. Direct and indirect costs and productivity losses

For direct costs for CHD and stroke, we sourced Estimates of National Medical Care Expenditure in 2019. Costs for productivity and informal care came from a 2017 study reporting the cost of illness for cancer, heart disease, and stroke in Japan.[32]

This study detailed total productivity losses for CHD and stroke through morbidity (limited to the time spent in hospital and outpatient visits, rather than a full spectrum of absenteeism and presenteeism) and early mortality, along with informal care costs described as the family burden of long-term care. The study did not report results by age or sex, leading us to apportion the total gross estimates by age and sex based on assumptions.
It was assumed that CHD and stroke-related productivity losses were incurred by age 30-75 only. The morbidity- and mortality-related productivity losses were apportioned based on age group and sex data on the proportion of the working population in 2016 from Ordinary Workers Data from the Ministry of Health, Labour and Welfare of Japan,[33] and CHD and stroke cases based on prevalence from the model for 2016 (the observed trend scenario) for morbidity; and working age population and mortality rates from the model for 2016 for mortality, multiplied by age and sex level population.

To estimate the informal care costs, total informal care costs were apportioned across all age groups from 30-34, 35-39… up to 85-89, then 90+. The costs were apportioned based on the proportion of disease cases in each age group and sex combination, and a study from the US[34] estimated weekly informal care hours provided for CHD and stroke by age group (54-64, 65-79, 80+). Cases aged under 30-54 were assumed to have the same number of informal care hours as 54–64-year-olds. We used the central estimates only, not the confidence intervals, and did not use the gender-based estimates. The year 2016 was chosen for consistency as most data was available for this year – the cost data were for 2014 and were inflated to 2021 costs using Japan CPI (all items).[35] Costs per person-year were calculated by dividing total costs in each gender-age group by the number of cases (prevalence * population) in each age group.

7. Strengths and limitations of this modelling approach

The $IMPACT_{NCD\text{-}JPN}$ model is an advanced, validated, flexible microsimulation of the dynamics of NCDs in a population, including important NCDs that are amenable to prevention and provide support to a range of

capabilities to conduct state-of-the-art effectiveness and cost-effectiveness analysis.

The complex dynamic generated by reductions in risk factors and disease trends needs to be modelled for a more realistic estimate of the future burden of illness from a societal perspective, which includes competing causes of illness and death. For instance, the model factors in that if CHD incidence is delayed, people may live longer and be more likely to get any of the other modelled diseases. This was evident in our projections, where, for example, the number of stroke cases increased because of people living longer. The modelling of individual conditions allows in-depth exploration of how trends in risk factors may impact the future burden of multimorbidity.

All such modelling analyses have limitations. This iteration of the model uses exposure data from NHNS waves up to 2019; however, we know that there have been changes in more recent years to both overall trends. Furthermore, the data that informs our model is from before the COVID-19 pandemic and, therefore, does not capture any of the impacts of the coronavirus or pandemic-related restrictions. The use of NHNS data from 1995-2019 was based on several reasons. Most importantly, during this time period, there is a consistent recording of risk factors based on age and sex over time. This allows us to separately model the projected incidence of long-term illness for different population subgroups. Although later versions of NHNS are available, differences in survey methodology meant it was not possible to include them in this model.

We derived trends in disease incidence and mortality from the GBD estimates and the Vital Statistics in Japan provided by Ministry of Health, Labour and Welfare, respectively. Whilst this source data largely represents the population of Japan, selection bias is unavoidable.

We sourced individual-level data on the aforementioned established CVD risk factor exposures from the NHNS for participants aged 20 to 99 between 1995 and 2019 or any available years in which the risk factor was measured if not consistently available throughout the entire period. For periods where data was unavailable for certain risk factors, back projections were performed. The results of the back projections were verified to align with the observed trends from the available data, and no issues were identified.

8. References

1. Collins B, Kypridemos C, Cookson R, Parvulescu P, McHale P, Guzman-Castillo M, et al. Universal or targeted cardiovascular screening? Modelling study using a sector-specific distributional cost effectiveness analysis. Prev Med. 2020;130:105879.
2. Collins B, Kypridemos C, Pearson-Stuttard J, Huang Y, Bandosz P, Wilde P, et al. FDA sodium reduction targets and the food industry: are there incentives to reformulate? Microsimulation cost-effectiveness analysis. Milbank Q. 2019;97:858–80.
3. Huang Y, Kypridemos C, Liu J, Lee Y, Pearson-Stuttard J, Collins B, et al. Cost-effectiveness of the US Food and Drug Administration added sugar labeling policy for improving diet and health. Circulation. 2019;139:2613–24.
4. Kypridemos C, Allen K, Hickey GL, Guzman-Castillo M, Bandosz P, Buchan I, et al. Cardiovascular screening to reduce the burden from cardiovascular disease: microsimulation study to quantify policy options. BMJ. 2016;353:i2793.
5. Kypridemos C, Collins B, McHale P, Bromley H, Parvulescu P, Capewell S, et al. Future cost-effectiveness and equity of the NHS Health Check cardiovascular disease prevention programme: microsimulation modelling using data from Liverpool, UK. PLoS Med. 2018;15:e1002573.
6. Kypridemos C, Guzman-Castillo M, Hyseni L, Hickey GL, Bandosz P, Buchan I, et al. Estimated reductions in cardiovascular and gastric cancer disease burden through salt policies in England: an $IMPACT_{NCD}$ microsimulation study. BMJ Open. 2017;7:e013791.
7. Laverty AA, Kypridemos C, Seferidi P, Vamos EP, Pearson-Stuttard J, Collins B, et al. Quantifying the impact of the public health responsibility deal on salt intake, cardiovascular disease and gastric cancer burdens: interrupted time series and microsimulation study. J Epidemiol Community Health. 2019;73:881–7.
8. Nilson EAF, Pearson-Stuttard J, Collins B, Guzman-Castillo M, Capewell S, O'Flaherty M, et al. Estimating the health and economic effects of the voluntary sodium reduction targets in Brazil: microsimulation analysis. BMC Med. 2021;19:225.
9. O'Flaherty M, Lloyd-Williams F, Capewell S, Boland A, Maden M, Collins B, et al. Modelling tool to support decision-making in the NHS Health Check programme: workshops, systematic review and co-production with users. Health Technol Assess Winch Engl. 2021;25:1–234.
10. Pearson-Stuttard J, Kypridemos C, Collins B, Mozaffarian D, Huang Y, Bandosz P, et al. Estimating the health and economic effects of the proposed US food and drug administration voluntary sodium reformulation: microsimulation cost-effectiveness analysis. PLoS Med. 2018;15:e1002551.
11. Ikeda N, Takimoto H, Imai S, Miyachi M, Nishi N. Data resource profile: the Japan national health and nutrition survey (NHNS). Int J Epidemiol. 2015;44:1842–9.
12. Ministry of Health L, Welfare. The national health and nutrition survey in Japan, 2019 [Internet]. Tokyo, Japan: Ministry of Health, Labour and Welfare; 2020. Available from: https://www.nibiohn.go.jp/eiken/kenkounippon21/en/eiyouchousa/

13. Ministry of Health L, Welfare. Vital statistics in Japan [Internet]. Tokyo, Japan: Ministry of Health, Labour and Welfare; 2023. Available from: https://www.mhlw.go.jp/english/database/db-hw/vs01.html
14. Kiyoshige E, Ogata S, O'Flaherty M, Capewell S, Takegami M, Iihara K, et al. Projections of future coronary heart disease and stroke mortality in Japan until 2040: a bayesian age-period-cohort analysis. Lancet Reg Health - West Pac. 2022;31:100637.
15. Ferrari AJ, Santomauro DF, Aali A, Abate YH, Abbafati C, Abbastabar H, et al. Global incidence, prevalence, years lived with disability (YLDs), disability-adjusted life-years (DALYs), and healthy life expectancy (HALE) for 371 diseases and injuries in 204 countries and territories and 811 subnational locations, 1990-2021: a systematic analysis for the global burden of disease study 2021. Lancet Lond Engl. 2024;403:2133–61.
16. Global Burden of Disease Collaborative Network. Global burden of disease study 2021 (GBD 2021) results [Internet]. Seattle, United States: Institute for Health Metrics and Evaluation (IHME); 2022 [cited 2024 Oct 9]. Available from: https://vizhub.healthdata.org/gbd-results/
17. Statistics Bureau of Japan. Population estimates [Internet]. Tokyo, Japan: Statistics Bureau of Japan; 2022. Available from: https://www.stat.go.jp/english/data/jinsui/index.html
18. National Institute of Population and Social Security Research. Population projections for Japan (2023 revision): 2021 to 2070 [Internet]. Tokyo, Japan: National Institute of Population and Social Security Research; 2023. Available from: https://www.ipss.go.jp/index-e.asp
19. Stasinopoulos MD, Rigby RA, Heller GZ, Voudouris V, De Bastiani F. Flexible regression and smoothing: using GAMLSS in R. Boca Raton: CRC Press/Taylor & Francis Group; 2017. 549 p.
20. Rigby RA, Stasinopoulos MD, Heller GZ, De Bastiani F. Distributions for Modelling Location, Scale, and Shape Using GAMLSS in R. [Internet]. New York: CRC Press LLC; 2019 [cited 2019 Nov 7]. Available from: http://public.ebookcentral.proquest.com/choice/publicfullrecord.aspx?p=5909942
21. Suen SC, Goldhaber-Fiebert JD, Basu S. Matching Microsimulation Risk Factor Correlations to Cross-sectional Data: The Shortest Distance Method. Med Decis Mak Int J Soc Med Decis Mak. 2018;38:452–64.
22. Embrechts P, Lindskog F, Mcneil A. Modelling Dependence with Copulas and Applications to Risk Management. In: Handbook of Heavy Tailed Distributions in Finance [Internet]. Elsevier; 2003 [cited 2020 Feb 13]. p. 329–84. Available from: https://linkinghub.elsevier.com/retrieve/pii/B9780444508966500108
23. Levin ML. The occurrence of lung cancer in man. Acta Unio Int Contra Cancrum. 1953;9:531–41.
24. Andersson T, Alfredsson L, Källberg H, Zdravkovic S, Ahlbom A. Calculating measures of biological interaction. Eur J Epidemiol. 2005;20:575–9.
25. Mehta N, Preston S. Are major behavioral and sociodemographic risk factors for mortality additive or multiplicative in their effects? Soc Sci Med 1982. 2016;154:93–9.
26. Comparative quantification of health risks [Internet]. Geneva: World Health Organisation; 2004 [cited 2014 Jan 30]. Available from: https://www.who.int/publications/i/item/9241580313

27. Murray CJL, Aravkin AY, Zheng P, Abbafati C, Abbas KM, Abbasi-Kangevari M, et al. Global burden of 87 risk factors in 204 countries and territories, 1990–2019: a systematic analysis for the global burden of disease study 2019. Lancet. 2020;396:1223–49.
28. Singh GM, Danaei G, Farzadfar F, Stevens GA, Woodward M, Wormser D, et al. The age-specific quantitative effects of metabolic risk factors on cardiovascular diseases and diabetes: a pooled analysis. PLOS ONE. 2013;8:e65174.
29. Stringhini S, Carmeli C, Jokela M, Avendaño M, Muennig P, Guida F, et al. Socioeconomic status and the 25 × 25 risk factors as determinants of premature mortality: a multicohort study and meta-analysis of 1·7 million men and women. Lancet. 2017;389:1229–37.
30. Koerkamp BG, Stijnen T, Weinstein MC, Hunink MGM. The combined analysis of uncertainty and patient heterogeneity in medical decision models. Med Decis Making. 2011;31:650–61.
31. Briggs AH, Weinstein MC, Fenwick EAL, Karnon J, Sculpher MJ, Paltiel AD. Model parameter estimation and uncertainty: a report of the ISPOR-SMDM modeling good research practices task force-6. Value Health. 2012;15:835–42.
32. Matsumoto K, Hanaoka S, Wu Y, Hasegawa T. Comprehensive cost of illness of three major diseases in Japan. J Stroke Cerebrovasc Dis Off J Natl Stroke Assoc. 2017;26:1934–40.
33. Ministry of Health L, Welfare. Ordinary workers data [Internet]. [cited 2024 Feb 26]. Available from: https://www.mhlw.go.jp/english/database/db-l/ordinary.html
34. Dunbar SB, Khavjou OA, Bakas T, Hunt G, Kirch RA, Leib AR, et al. Projected costs of informal caregiving for cardiovascular disease: 2015 to 2035: a policy statement from the american heart association. Circulation. 2018;137:e558–77.
35. Statistics Bureau M of IA, Communications. 2020-base explanation of the consumer price index [Internet]. Tokyo, Japan: Statistics Bureau, Ministry of Internal Affairs and Communications; 2022 [cited 2024 Mar 8]. Available from: https://www.stat.go.jp/english/data/cpi/1590.html
36. Fukuda H, Ninomiya H, Ueba Y, Ohta T, Kaneko T, Kadota T, et al. Impact of temperature decline from the previous day as a trigger of spontaneous subarachnoid hemorrhage: case-crossover study of prefectural stroke database. J Neurosurg. 2019;133:374–82.
37. Kojima S, Michikawa T, Tsujita K. Three-year follow-up of the impact of kumamoto earthquake on acute myocardial infarctions: an interrupted time series analysis. Am Heart J Plus Cardiol Res Pract. 2023;26:100246.

A) Supplementary Material A: Disease Module Details and Validation

The following subsections present data sources, structural diagrams, and validation plots for each condition.

Model structure

Figure A-1 is a 'birds-eye' diagrammatic presentation of all the relationships modelled between risk factors and conditions (i.e., CHD and stroke in the present IMPACT $_{NCD}$ Japan), as described in the above sections. Note that IMPACT$_{NCD-JPN}$ and, consequently, the plot below include two exposures that we haven't described so far because they were irrelevant to this study. These are antidiabetic medication (Med_DM) and antihypertensive medication (Med_HT) that we use to define two additional diseases, type 2 diabetes mellitus (t2dm) and hypertension (htn) that are also irrelevant to this study.

Figure A-1. Relationships modelled between exposures and conditions.

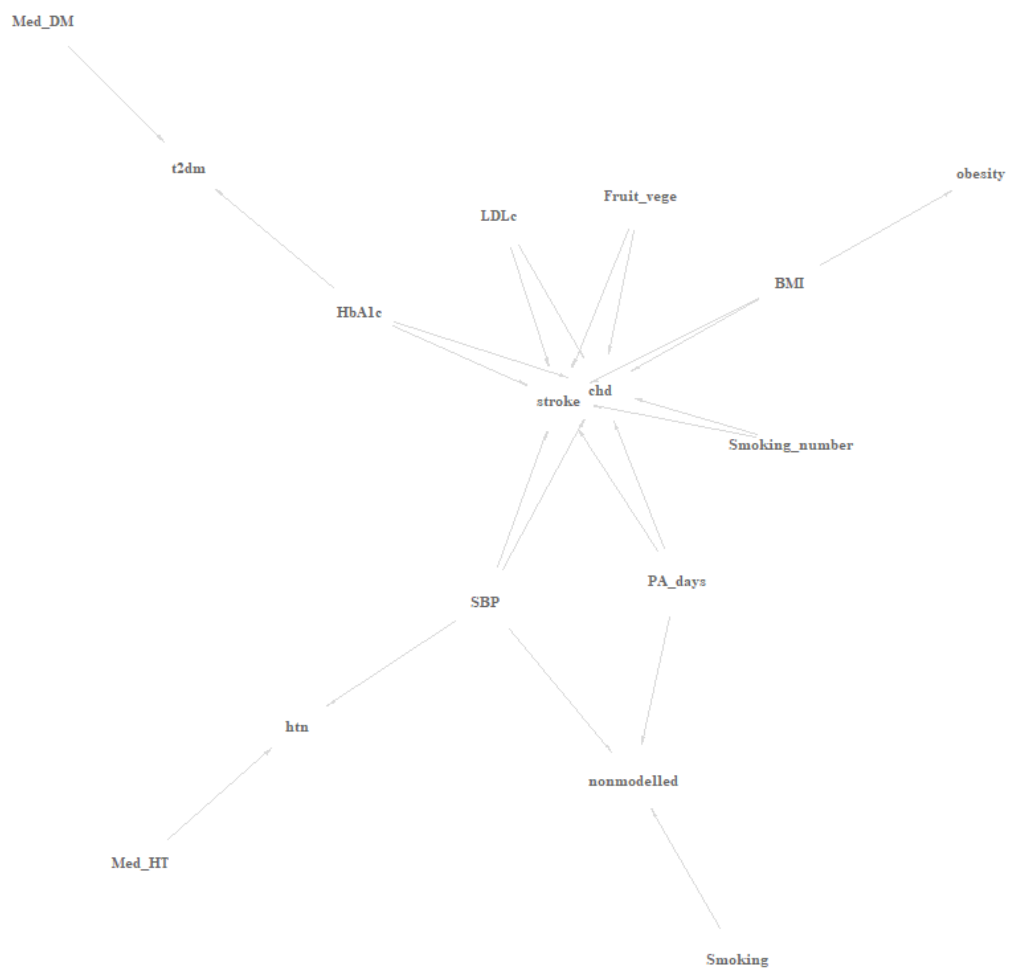

Coronary Heart Disease (CHD)

Table A-1 Modelling assumptions for incident CHD

**Component**	**Assumptions / Details**
Disease type	Chronic – no recovery
Risk factor associations	Smoking, fruit and vegetable intake, physical activity, body mass index, systolic blood pressure, low-density lipoprotein cholesterol, and hemoglobin A1c
Disease dependencies	NA
**Data resources**	The incidence and prevalence of CHD were derived from those of "ischemic heart disease" (ICD-10 code I20-I25) estimated by the Global Burden of Diseases, Injuries, and Risk Factors Study (GBD) 2021 results version (Global Burden of Disease Collaborative Network. Global burden of disease study 2021 (GBD 2021) results [Internet]. Seattle, United States: Institute for Health Metrics and Evaluation (IHME); 2022 [cited 2024 Oct 9]. Available from: https://vizhub.healthdata.org/gbd-results/).<br><br>Mortality from CHD was derived from mortality from "acute myocardial infarction" and "other ischemic heart diseases" (ICD-10 codes I20-I22, I24, and I25) reported in the Vital Statistics in Japan provided by the Ministry of Health, Labour and Welfare. Although ICD-10 code I23 (certain current complications after acute myocardial infarction) is excluded from the official Japanese Vital Statistics mortality statistics, deaths originally certified as I23 are routinely reclassified to I21 or I22 (acute myocardial infarction or subsequent myocardial infarction) as the underlying cause of death in accordance with coding guidelines. Therefore, there is no significant loss of mortality data due to this exclusion. This classification practice is based on ICD-10 (2013 revision) and is implemented in the vital statistics published by the Ministry of Health, Labour and Welfare, Japan (Ministry of Health L, Welfare. Vital statistics in Japan [Internet]. Tokyo, Japan: Ministry of Health, Labour and Welfare; 2023. Available from: https://www.mhlw.go.jp/english/database/db-hw/vs01.html).

Figure A-2. Causal structure of risk factor associations and disease dependencies for incident CHD

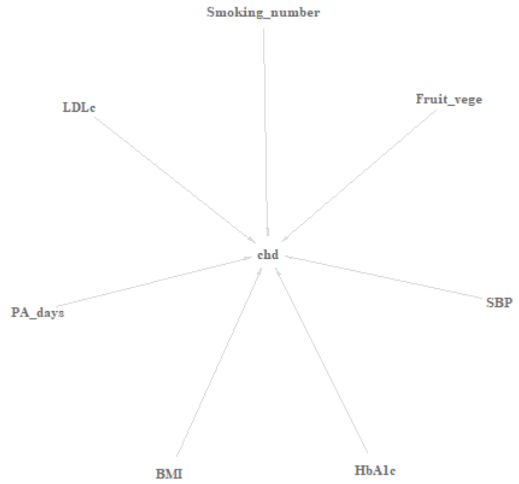

Table A-2 - Data sources for causal associations between risk factors and CHD incidence

Parameter	Details	Comments	Source
Relative risk for active smoking	GBD meta-analysis	RR from the GBD 2019 study by smoking intensity	Global Burden of Disease Study 2019 (GBD 2019) Data Resources | GHDx [Internet]. [cited 2022 Mar 16];Available from: http://ghdx.healthdata.org/record/ihme-data/gbd-2019-relative-risks
Relative risk for systolic blood pressure	Meta-analysis of individual data from 61 prospective studies	Stratified by age and sex. Adjusted for regression dilution and total blood cholesterol and, where available, lipid fractions (HDL and non-HDL cholesterol), diabetes, weight, alcohol consumption, and smoking at baseline.	Age-specific relevance of usual blood pressure to vascular mortality: a meta-analysis of individual data for one million adults in 61 prospective studies. The Lancet 2002;360:1903–13. (Figures 3 and 5)
Relative risk for low-density lipoprotein cholesterol	Meta-analysis of individual data from 115 prospective studies	Adjusted for age, sex, SBP, smoking status, history of diabetes, BMI, HDL-c, and triglyceride. We extrapolate HR for people aged between 30-39 using 40-59.	Major lipids, apolipoproteins, and risk of vascular disease. Emerging Risk Factors Collaboration, et al. JAMA. 2009 Nov 11;302(18):1993-2000. (eFgiure 2; non-HDL-c corresponding to LDL-c due to adjustment for HDL-c and TG.)
Relative risk for body mass index	Meta-analysis of 58 prospective studies	Stratified by age. Adjusted for age, sex, smoking status, systolic blood pressure, history of diabetes, and total and HDL cholesterol.	The Emerging Risk Factors Collaboration. Separate and combined associations of body-mass index and abdominal adiposity with cardiovascular disease: collaborative analysis of 58 prospective studies. The Lancet 2011;377:1085–95. (Table 1 and Figure 2)
Relative risk for hemoglobin A1c	Meta-analysis of 8 prospective studies	Adjusted for age, sex, blood pressure, smoking, BMI, duration of diabetes mellitus, and lipid in most of the meta-analyzed studies.	Zhang Y, et al. Glycosylated hemoglobin in relationship to cardiovascular outcomes and death in patients with type 2 diabetes: a systematic review and meta-analysis. PLoS One. 2012;7(8):e42551. (Figure 2)
Relative risk for physical activity	Meta-analysis of 18 cohort studies for CHD and 8 cohort studies for ischemic stroke	Stratified by age and sex. Adjusted for measurement error, age, sex, smoking, blood pressure and cholesterol.	Bull FC, Armstrong TP, Dixon T, Ham S, Neiman A, Pratt M. Comparative quantification of health risks. Chapter 10: physical inactivity. Geneva: World Health Organization; 2004. (Tables 10.19 and 10.20)
Relative risk for fruit and vegetable consumption	Meta-analysis of 9 cohort studies	RR per portion of F&V. Multiply-adjusted.	Dauchet L, Amouyel P, Hercberg S, Dallongeville J. Fruit and Vegetable Consumption and Risk of Coronary Heart Disease: A Meta-Analysis of Cohort Studies. J Nutr 2006;136:2588–93.

Parameter	Details	Comments	Source
Exposure levels below which no excess risk for cardiometabolic disease is assumed for SBP, BMI, and total cholesterol	Pooling of 123 cohorts	Data on 1.4 million individuals and 52,000 CVD events. We assumed the levels are similar for other diseases beyond cardiometabolic.	Singh GM, Danaei G, Farzadfar F, Stevens GA, Woodward M, Wormser D, et al. The age-specific quantitative effects of metabolic risk factors on cardiovascular diseases and diabetes: a pooled analysis. PLOS ONE 2013;8:e65174.

Validation plots for CHD incidence and mortality

Figure A-3. Validation plot for modelled CHD incidence by sex

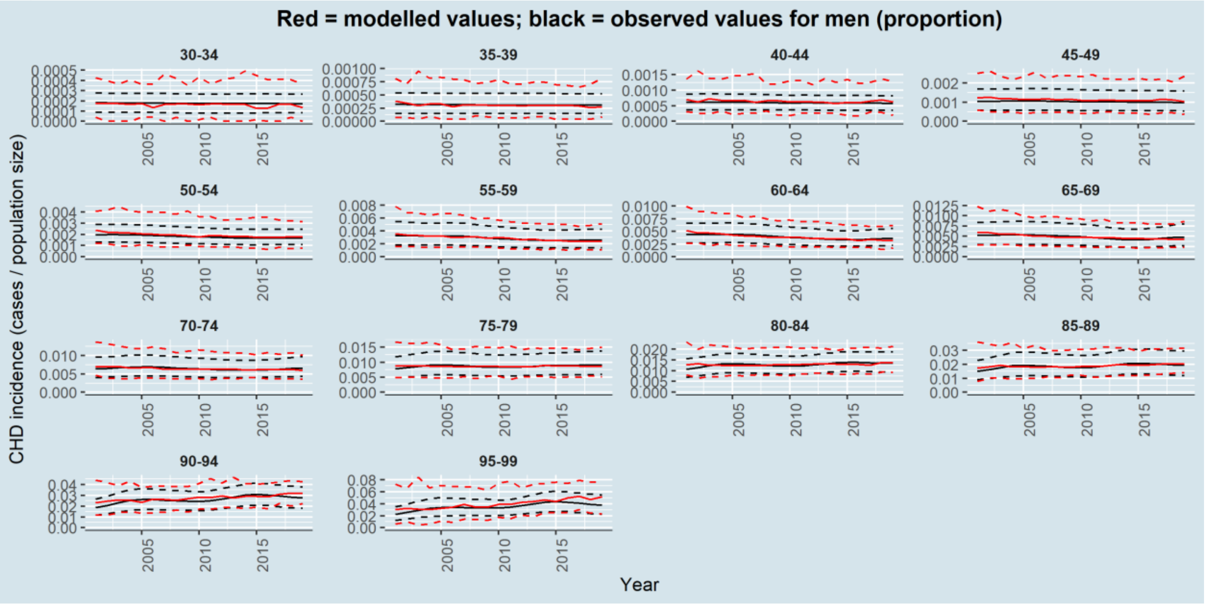

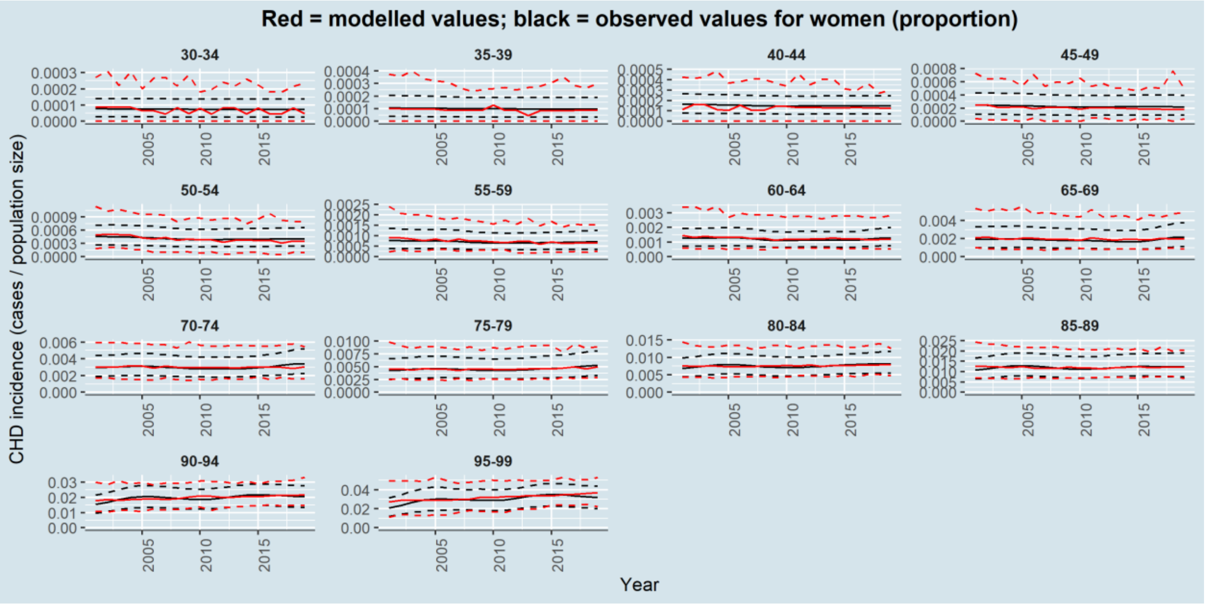

Figure A-4. Validation plot for modelled CHD mortality by sex

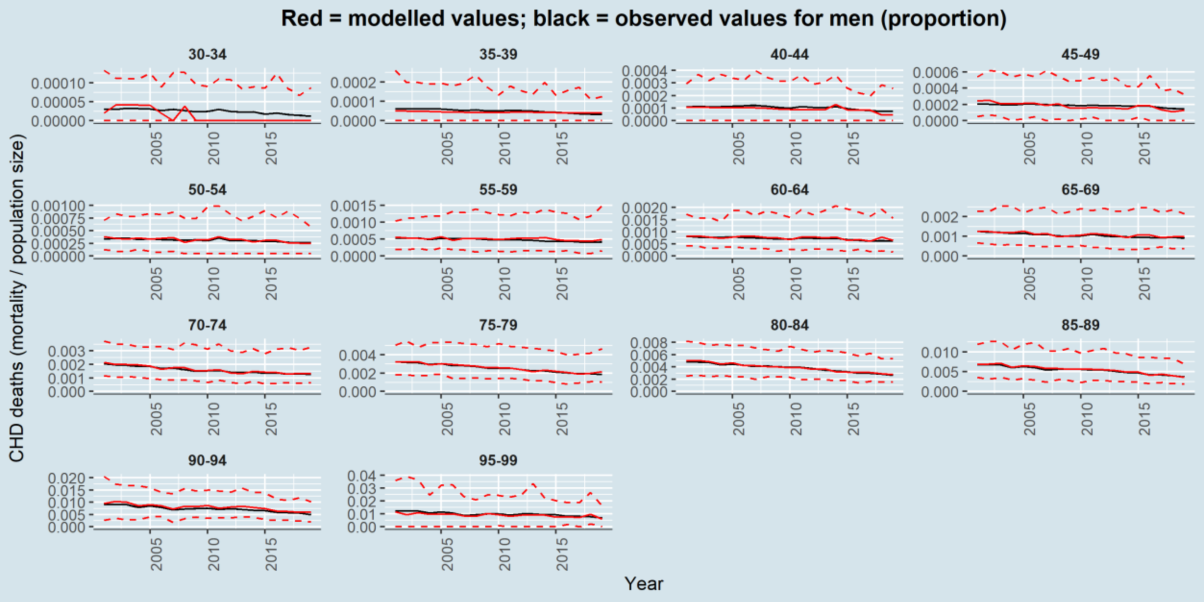

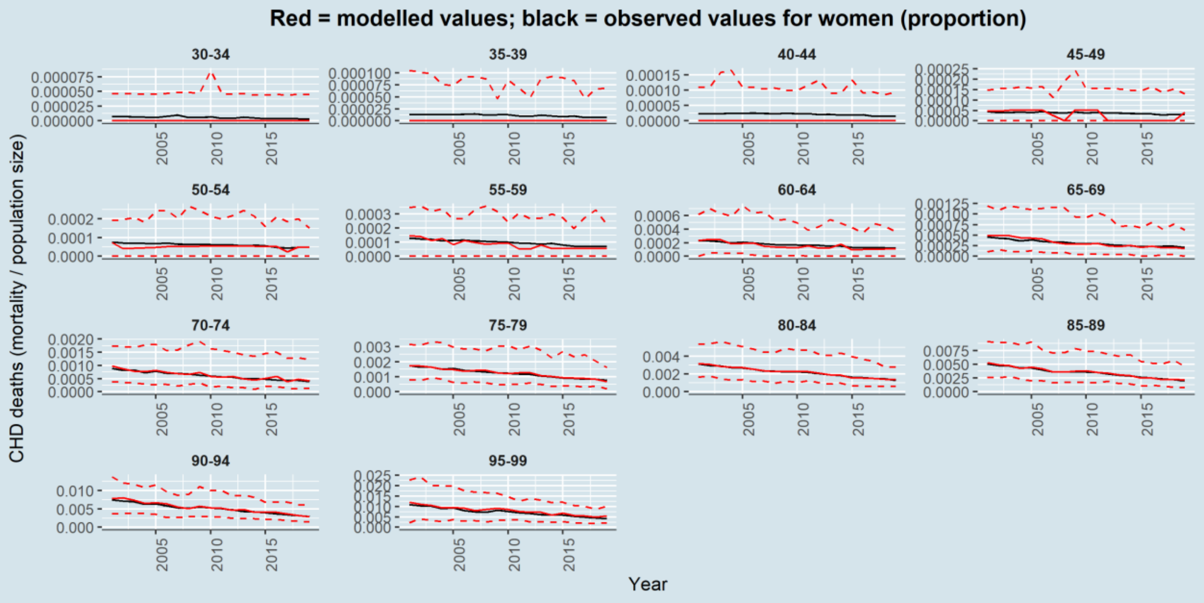

Note: In women aged 30–49, observed CHD mortality rates are very low, so small absolute differences may appear as large relative gaps. All observed rates (black lines) remain within the model's 95% uncertainty intervals (red dashed lines).

Stroke (ischemic stroke and hemorrhagic stroke, including subarachnoid hemorrhage)

Table A-3 - Modelling assumptions for incident stroke

Component	Assumptions / Details
Disease type	Chronic – no recovery
Risk factor associations	Smoking, fruit and vegetable intake, physical activity, body mass index, systolic blood pressure, low-density lipoprotein cholesterol, and hemoglobin A1c
Disease dependencies	NA
**Data resources**	The incidence and prevalence of stroke were derived from those of “stroke” (I60-I69 of the ICD-10 code) estimated by the Global Burden of Diseases, Injuries, and Risk Factors Study (GBD) 2021 results version (Global Burden of Disease Collaborative Network. Global burden of disease study 2021 (GBD 2021) results [Internet]. Seattle, United States: Institute for Health Metrics and Evaluation (IHME); 2022 [cited 2024 Oct 9]. Available from: https://vizhub.healthdata.org/gbd-results/).<br><br>Mortality from stroke was derived from that from “stroke” (I60- I69 of the ICD-10 code) reported in Vital Statistics in Japan, provided by the Ministry of Health, Labour and Welfare (Ministry of Health L, Welfare. Vital statistics in Japan [Internet]. Tokyo, Japan: Ministry of Health, Labour and Welfare; 2023. Available from: https://www.mhlw.go.jp/english/database/db-hw/vs01.html)

Figure A-5. Causal structure of risk factor associations and disease dependencies for incident stroke

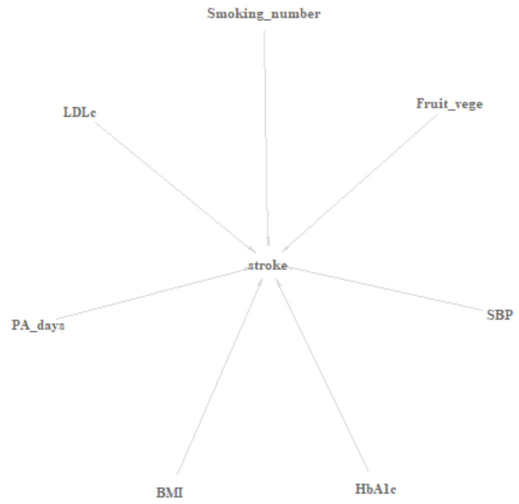

Table A-4 - Data sources for causal associations between risk factors and stroke incidence

Parameter	Details	Comments	Source
Relative risk for active smoking	GBD meta-analysis	RR from the GBD 2019 study	Global Burden of Disease Study 2019 (GBD 2019) Data Resources | GHDx [Internet]. [cited 2022 Mar 16];Available from: http://ghdx.healthdata.org/record/ihme-data/gbd-2019-relative-risks
Relative risk for systolic blood pressure	Meta-analysis of individual data from 61 prospective studies	Stratified by age and sex. Adjusted for regression dilution and total blood cholesterol and, where available, lipid fractions (HDL and non-HDL cholesterol), diabetes, weight, alcohol consumption, and smoking at baseline.	Age-specific relevance of usual blood pressure to vascular mortality: a meta-analysis of individual data for one million adults in 61 prospective studies. The Lancet 2002;360:1903–13. (Figures 3 and 5)
Relative risk for low-density lipoprotein cholesterol	Meta-analysis of individual data from 32 prospective studies	Adjusted for age, sex, SBP, smoking status, history of diabetes, BMI, HDL-c, and TG We extrapolate HR for people aged between 30-39 using 40-59.	Major lipids, apolipoproteins, and risk of vascular disease. Emerging Risk Factors Collaboration, et al. JAMA. 2009 Nov 11;302(18):1993-2000. (P1997 left top in results section and eTable 5 for ischemic stroke: non-HDL-c corresponding to LDL-c due to adjustment for HDL-c and TG (1.12 [1.04-1.20]). Based on the HR of ischemic stroke, we obtained adjusted HR of all types stroke by ratios of non-ischemic stroke to ischemic stroke)
Relative risk for body mass index	Meta-analysis of 58 prospective studies	Stratified by age. Adjusted for age, sex, smoking status, systolic blood pressure, history of diabetes, and total and HDL cholesterol.	The Emerging Risk Factors Collaboration. Separate and combined associations of body-mass index and abdominal adiposity with cardiovascular disease: collaborative analysis of 58 prospective studies. The Lancet 2011;377:1085–95. (Table 1 and Figure 2)
Relative risk for hemoglobin A1c	Meta-analysis of 4 prospective studies	Adjusted for age, sex, blood pressure, smoking, and lipid in most of the meta-analyzed studies.	Zhang Y, et al. Glycosylated hemoglobin in relationship to cardiovascular outcomes and death in patients with type 2 diabetes: a systematic review and meta-analysis. PLoS One. 2012;7(8):e42551. (Figure 2)
Relative risk for physical activity	Meta-analysis of 18 cohort studies for CHD and 8 cohort studies for ischemic stroke	Stratified by age and sex. Adjusted for measurement error, age, sex, smoking, blood pressure and cholesterol.	Bull FC, Armstrong TP, Dixon T, Ham S, Neiman A, Pratt M. Comparative quantification of health risks. Chapter 10: physical inactivity. Geneva: World Health Organization; 2004. (Tables 10.19 and 10.20)
Relative risk for fruit and vegetable consumption	Meta-analysis of 7 cohort studies	RR per portion of F&V. Multiply-adjusted.	Dauchet L, Amouyel P, Dallongeville J. Fruit and vegetable consumption and risk of stroke A meta-analysis of cohort studies. Neurology 2005;65:1193–7.

Validation plots for stroke incidence and mortality

Figure A-6. Validation plot for modelled stroke incidence for by sex

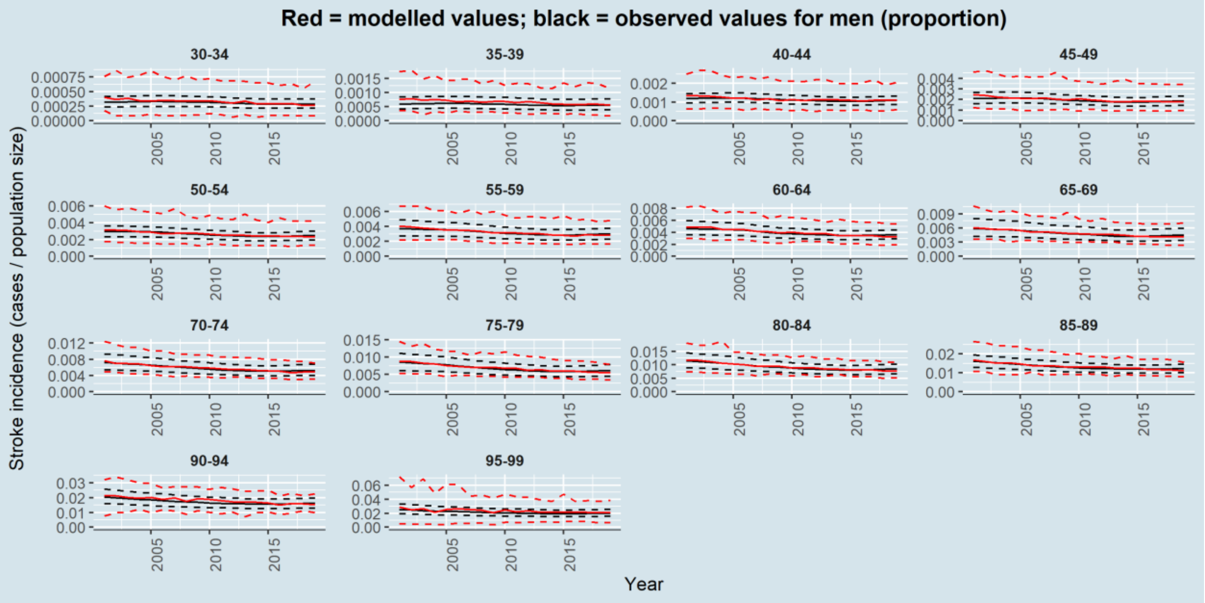

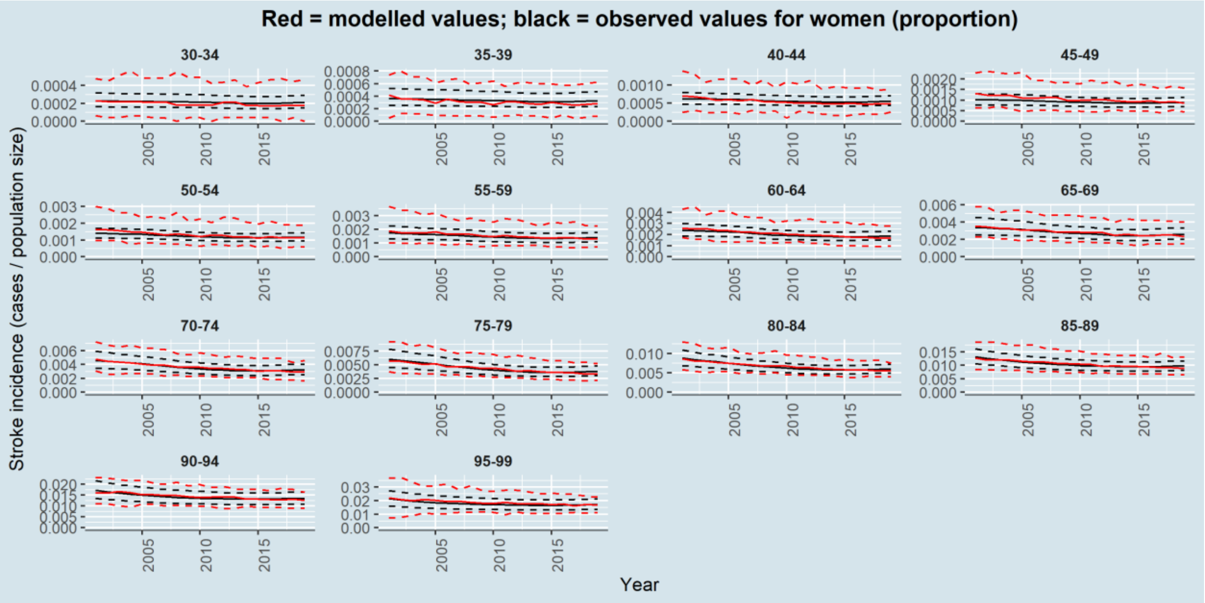

Figure A-7. Validation plot for modelled stroke mortality by sex

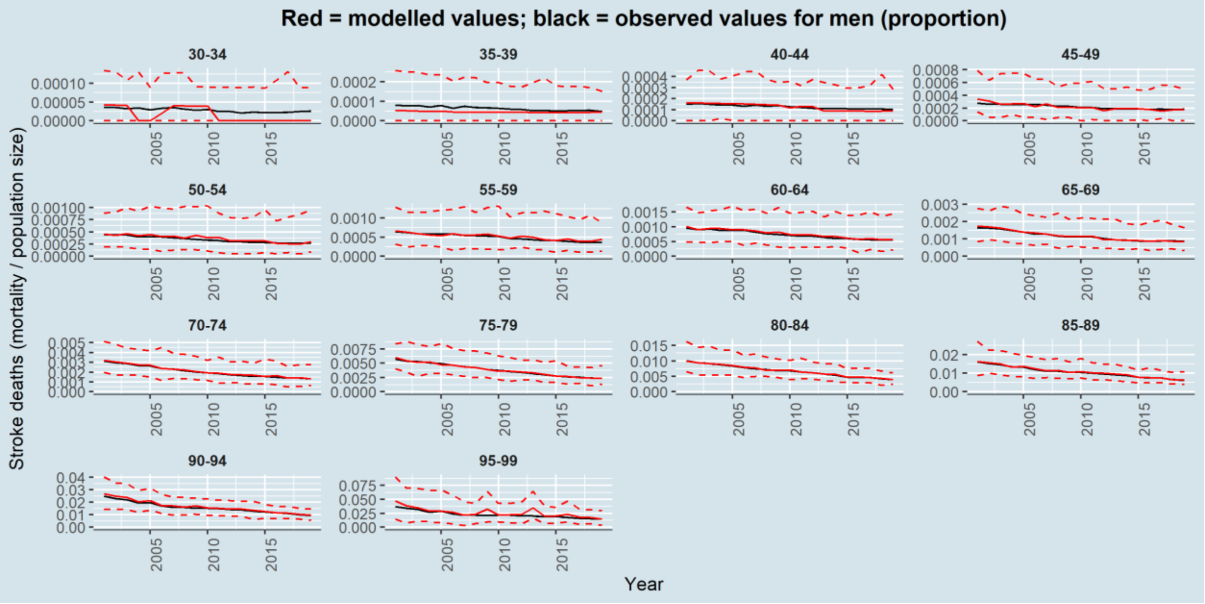

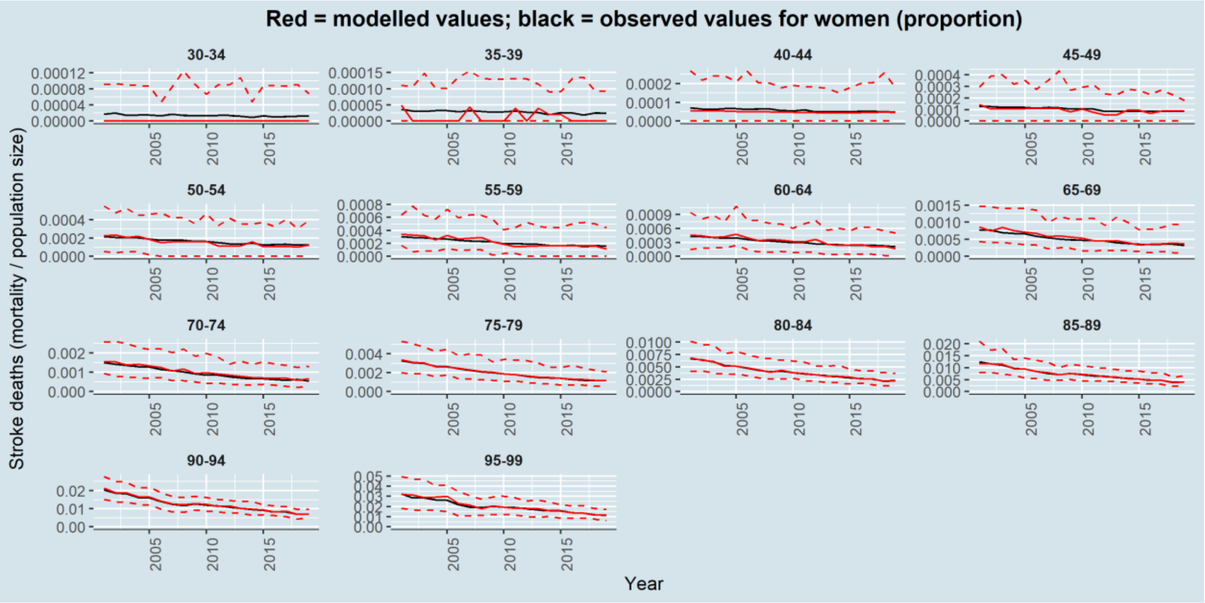

Non-modelled mortality

Table A-5 Modelling assumptions for mortality for causes other than the case fatalities described above

Component	Assumptions / Details
Outcome type	Mortality from any cause other than CHD and stroke
Risk factor associations	Smoking, systolic blood pressure, physical activity
Disease dependencies	None

Figure A-8. Causal structure of risk factor associations and disease dependencies for incident non-modelled mortality

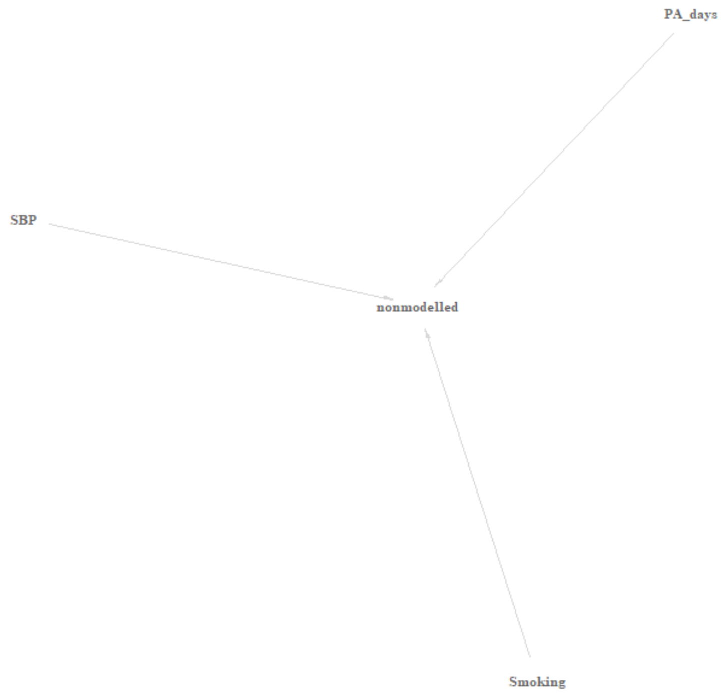

Table A-6 Data sources for causal associations between risk factors and non-modelled mortality

Parameter	Details	Comments	Source
Relative risk for smoking status	Meta-analysis of 1.7 million men and women	Multiply adjusted. We used the Non-CVD, non-cancer mortality effects.	Stringhini S, Carmeli C, Jokela M, Avendaño M, Muennig P, Guida F, et al. Socioeconomic status and the 25 × 25 risk factors as determinants of premature mortality: a multicohort study and meta-analysis of 1·7 million men and women. The Lancet 2017;389:1229–37. (Figure 4)
Relative risk for systolic blood pressure	Meta-analysis of 1.7 million men and women	Multiply adjusted. We used the Non-CVD, non-cancer mortality effects. We applied the effect to those with SBP > 140 mmHg	Stringhini S, Carmeli C, Jokela M, Avendaño M, Muennig P, Guida F, et al. Socioeconomic status and the 25 × 25 risk factors as determinants of premature mortality: a multicohort study and meta-analysis of 1·7 million men and women. The Lancet 2017;389:1229–37. (Figure 4)
Relative risk for physical activity	Meta-analysis of 1.7 million men and women	Multiply adjusted. We used the Non-CVD, non-cancer mortality effects. We applied the effect only to those with one or less active days per week.	Stringhini S, Carmeli C, Jokela M, Avendaño M, Muennig P, Guida F, et al. Socioeconomic status and the 25 × 25 risk factors as determinants of premature mortality: a multicohort study and meta-analysis of 1·7 million men and women. The Lancet 2017;389:1229–37. (Figure 4)

Validation plots for non-modelled mortality

Figure A-9. Validation plot for non-modelled mortality by sex

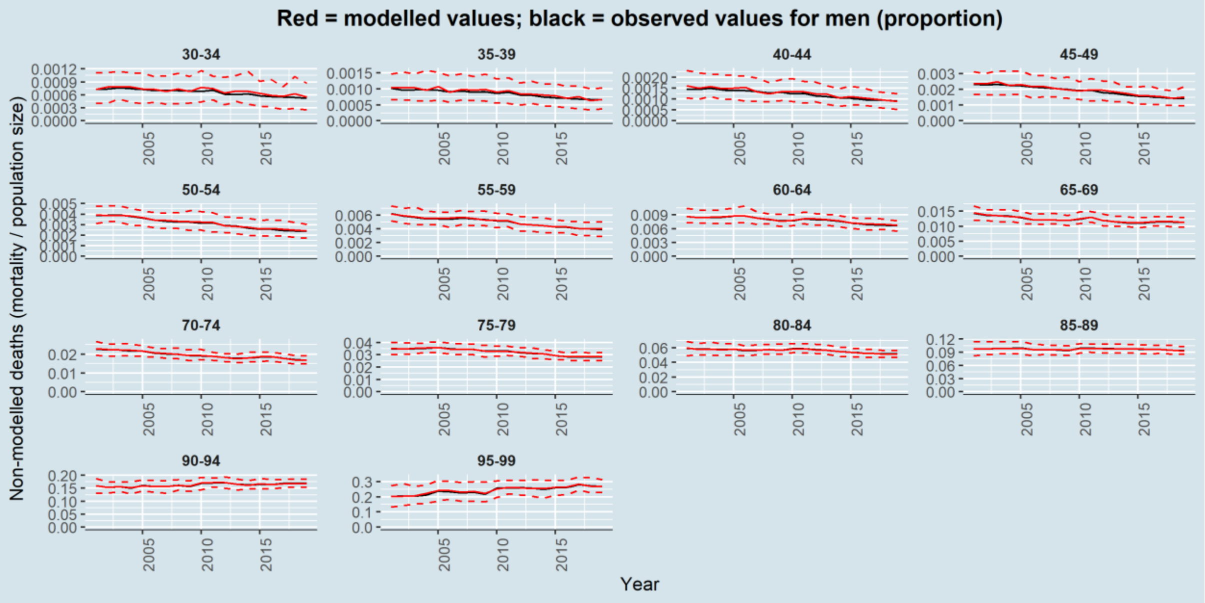

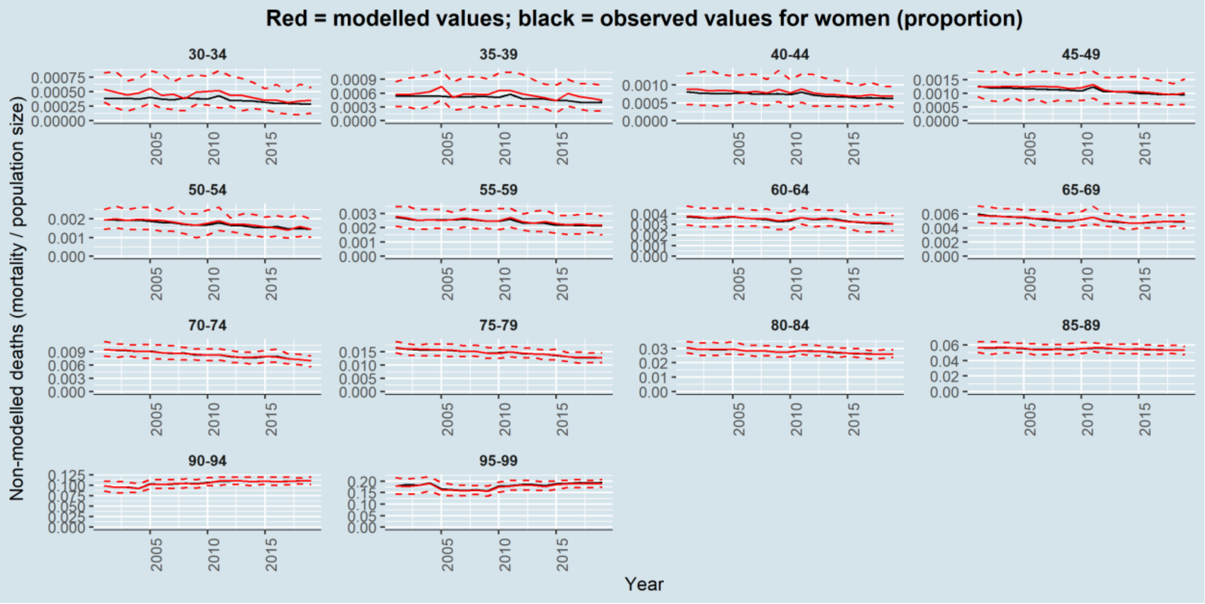

B) Supplementary Material B: Exposure Modelling Details and Validation

Exposure modelling details

Table B-1 - Exposure modelling in IMPACT$_{NCD-JPN}$

Exposure	Statistical Modelling (distribution)	Independent Variables	Comment
Active days per week	Ordered logistic regression	Year, age, sex	Active days per week was ordinally modelled as 0, 1, 2, 3, 4, 5, 6, and 7.
Daily fruit and vegetable consumption in grams	GAMLSS (Zero Altered Negative binomial type I)	Year, age, sex	The detailed methodology of the dietary assessment in NHNS has been described elsewhere.[11,12] NHNS employed a one-day dietary recording approach that combined weighing and household measurement methods. The survey period was set in October–November, excluding weekends, public holidays, and days of irregular food consumption. The main household meal preparers documented the dietary intake of all members aged one year and above. Trained professionals, primarily registered dietitians, distributed dietary record booklets and provided instructions through both written materials and face-to-face guidance.<br>Participants were asked to measure food items using scales whenever feasible and to use household measuring tools for seasonings and condiments. When weighing was impractical, such as for pre-packaged meals, they estimated portion sizes and listed as many ingredient details as possible. Additional data on food wastage and meal-sharing within the household was also recorded. Interviewers later revisited each home to collect, verify, and supplement missing or unclear information.<br>The dietary data were coded following Japan's Standard Tables of Food Composition, with necessary conversions applied for mixed dishes and processed foods. Nutrient intake was calculated based on these standards. The reliability of this method was previously evaluated by comparing dietary records from trained dietetic students and meal preparers, revealing a high degree of consistency.
Current smoking status	GAMLSS (binomial)	Year, age, sex, tabaco tax changes	Tabaco tax changes were categorical variables as "0" for 2003-2005, "1" for 2006-2009, "2" for 2010-2017, and 3 for 2018-2019.
Ex-smoking status	GAMLSS (binomial)	Year, age, sex	
Cigarettes per day for current smokers	Ordered logistic regression	Year, age, sex	The number of cigarettes per day for current smokers was ordinally modelled 1-5, 6-10, 11-15, 16-20, 21-25, 26-30, 31-35, 36-40, and more than 40.
BMI	GAMLSS (Box-Cox t with log link for mean)	Year, age, sex, physical activity	
HbA1c	GAMLSS (Box-Cox t)	Year, age, sex, medication use for	

Exposure	Statistical Modelling (distribution)	Independent Variables	Comment
		diabetes, BMI	
Diabetes mellitus medication	GAMLSS (binomial)	Year, age, sex	
SBP	GAMLSS (Box-Cox Power Exponential)	Year, age, sex, BMI, smoking, medication use for hypertension	
Hypertension medication	GAMLSS (binomial)	Year, age, sex	
LDL-c	GAMLSS (Box-Cox t)	Year, age, sex, BMI, medication use for hyperlipidemia	
Hyperlipidemia medication	GAMLSS (binomial)	Year, age, sex	

Exposure validation plots

The following figures are the cumulative probability validation plots for the simulated exposures compared to the observed National Health Nutrition Survey for Japan data. The plots are presented by year and 10-year age group for each sex.

We also present trends in exposure for the main exposures modelled (BMI, SBP, LDL-c, HbA1c, fruit and vegetable intake, active days, smoking prevalence).

Body mass index

Figure B-1. Validation: BMI – NHNS and projected IMPACT$_{\text{NCD-JPN}}$ trends by year, age, and sex

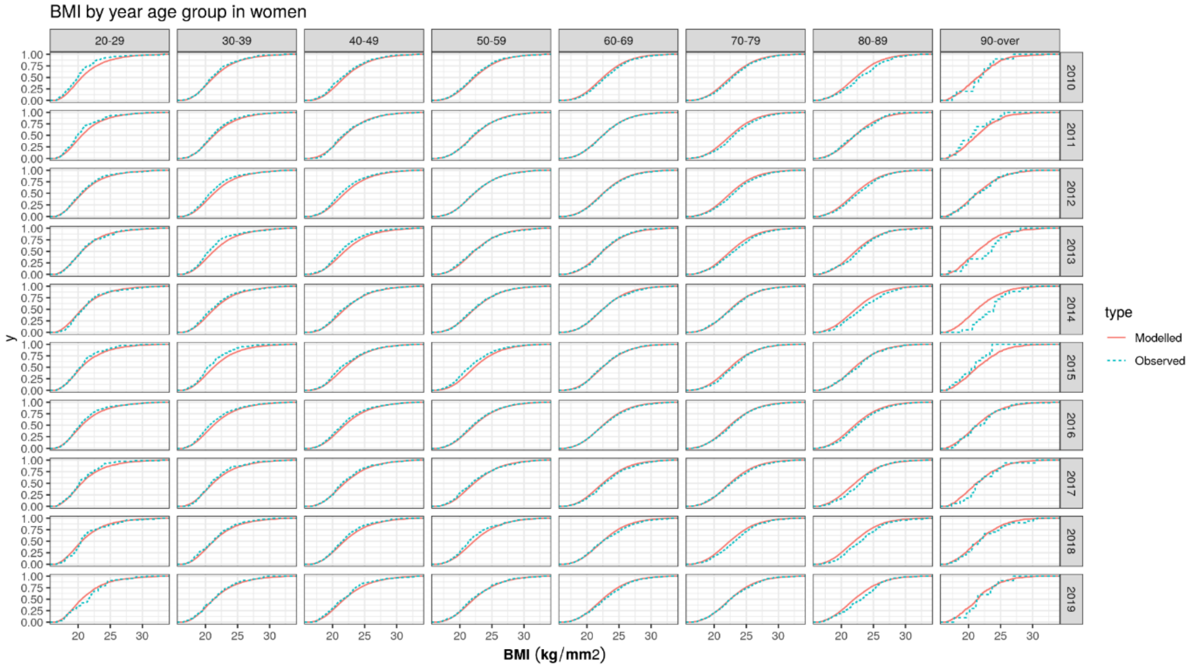

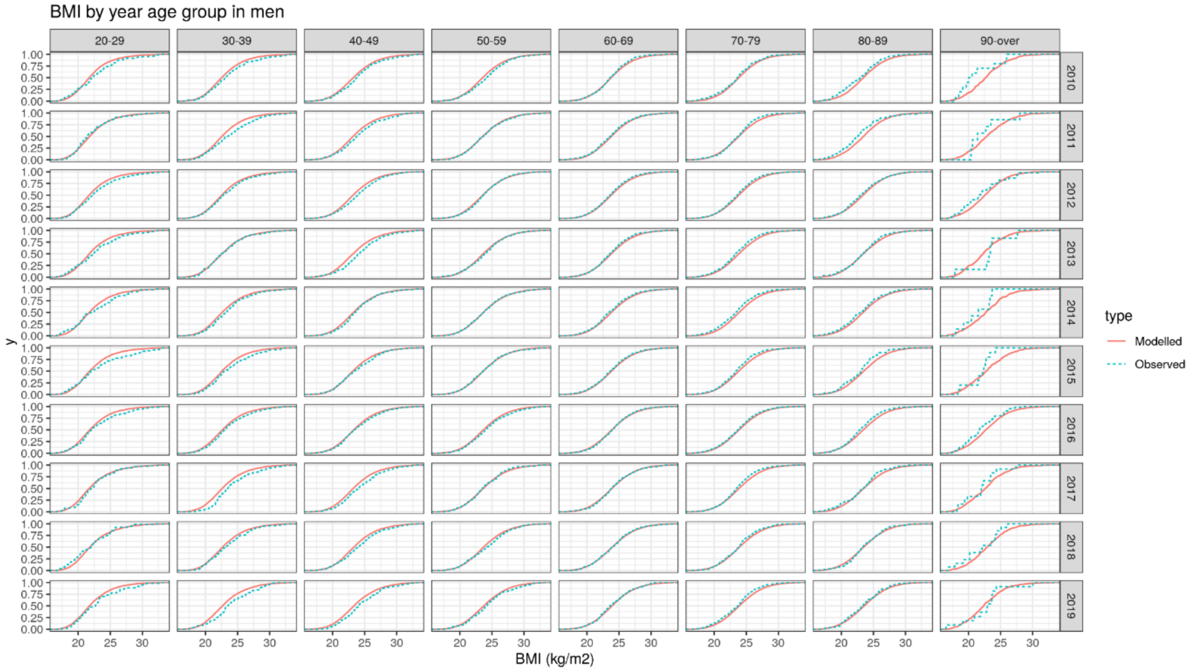

Figure B-2. Validation: BMI – NHNS and projected IMPACT$_{NCD-JPN}$ trends by year, age, and sex

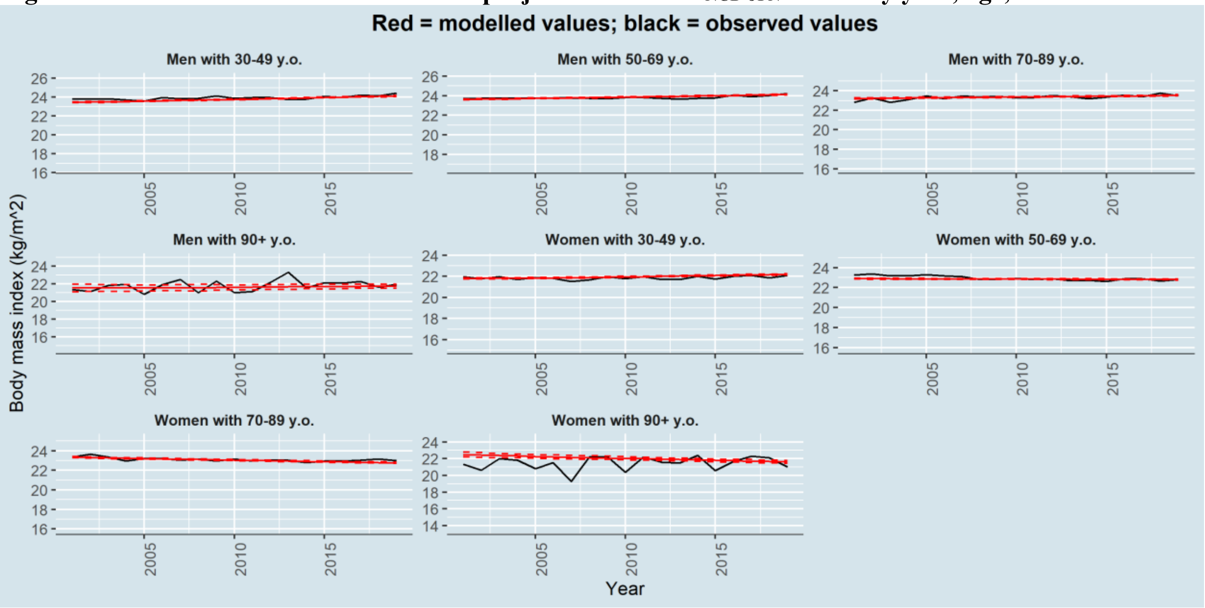

Smoking status

Figure B-3. Validation: Proportion of current smokers – NHNS and projected IMPACT$_{NCD\text{-}JPN}$ trends by year, age, and sex

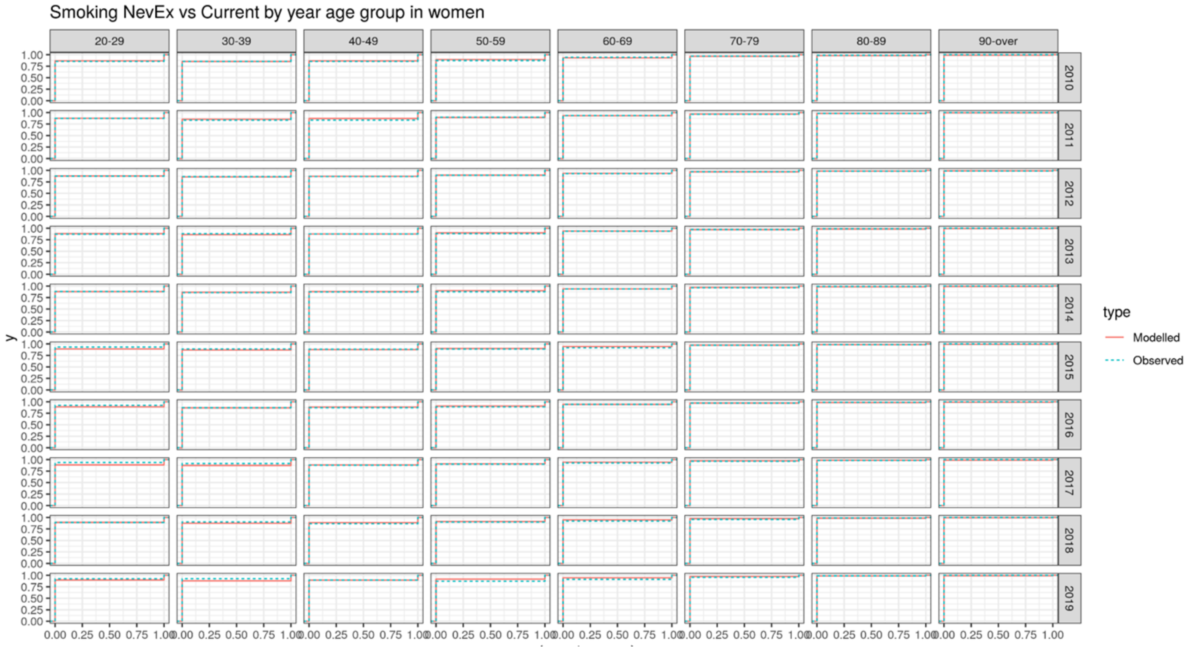

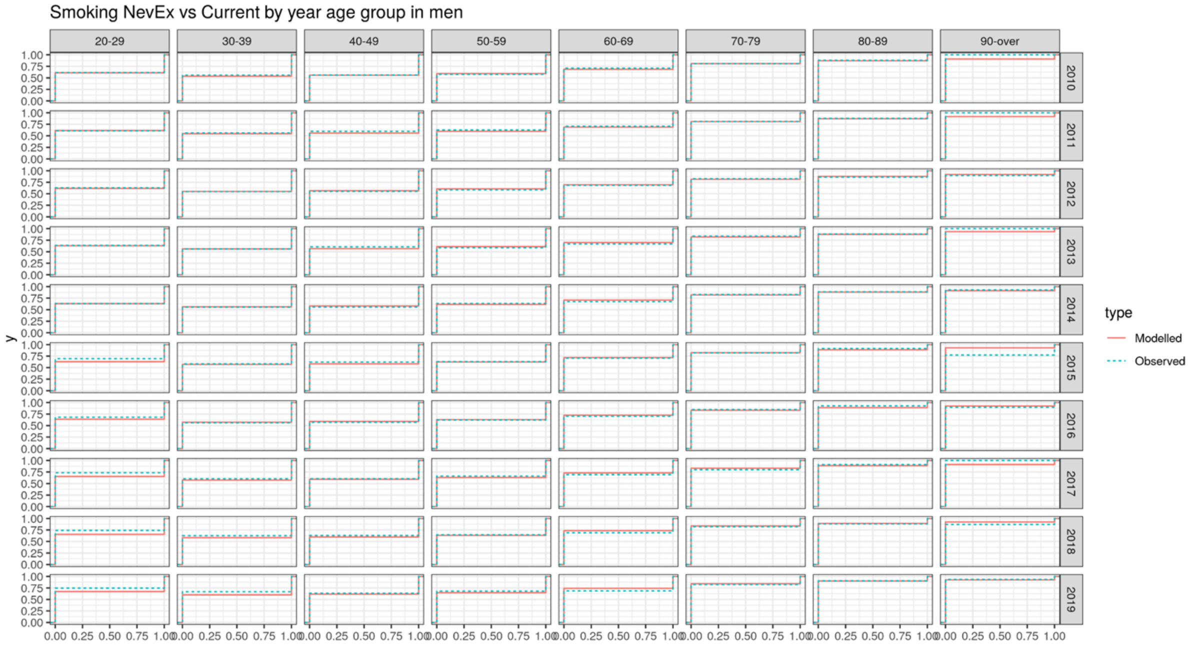

Figure B-4. Validation: Proportion of ex-smokers – NHNS and projected IMPACT$_{NCD-JPN}$ trends by year, age, and sex

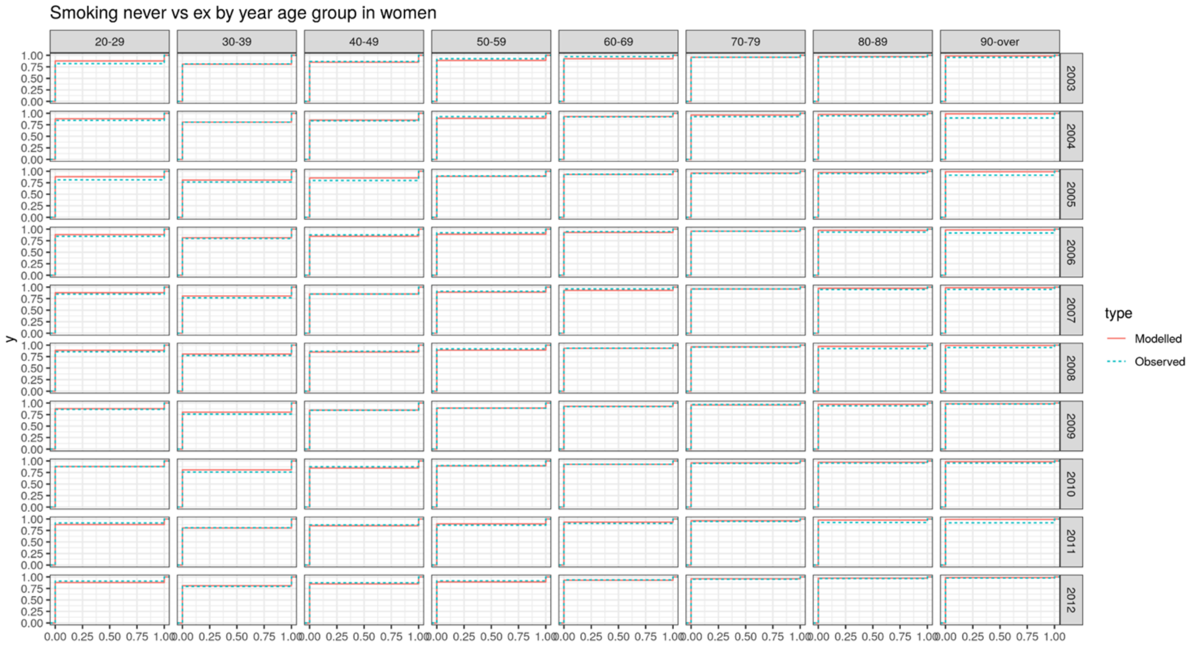

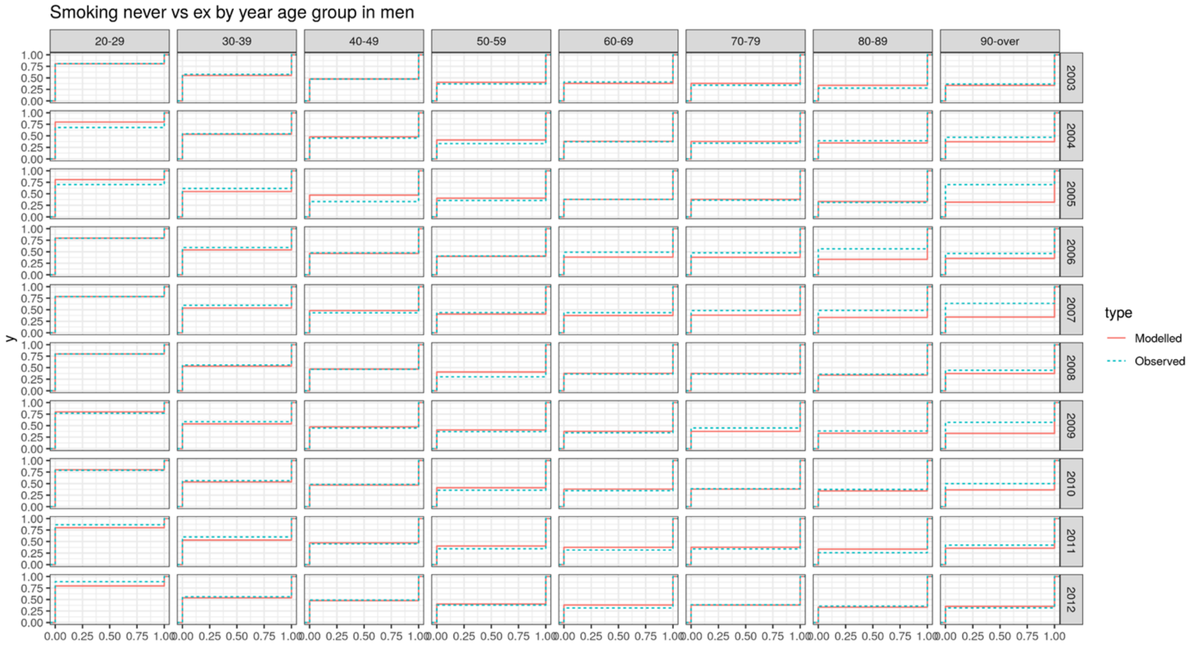

Number of cigarettes smoked (current smokers)

Figure B-5. Validation: Number of cigarettes smoked – NHNS and projected IMPACT$_{NCD-JPN}$ trends by year, age, and sex

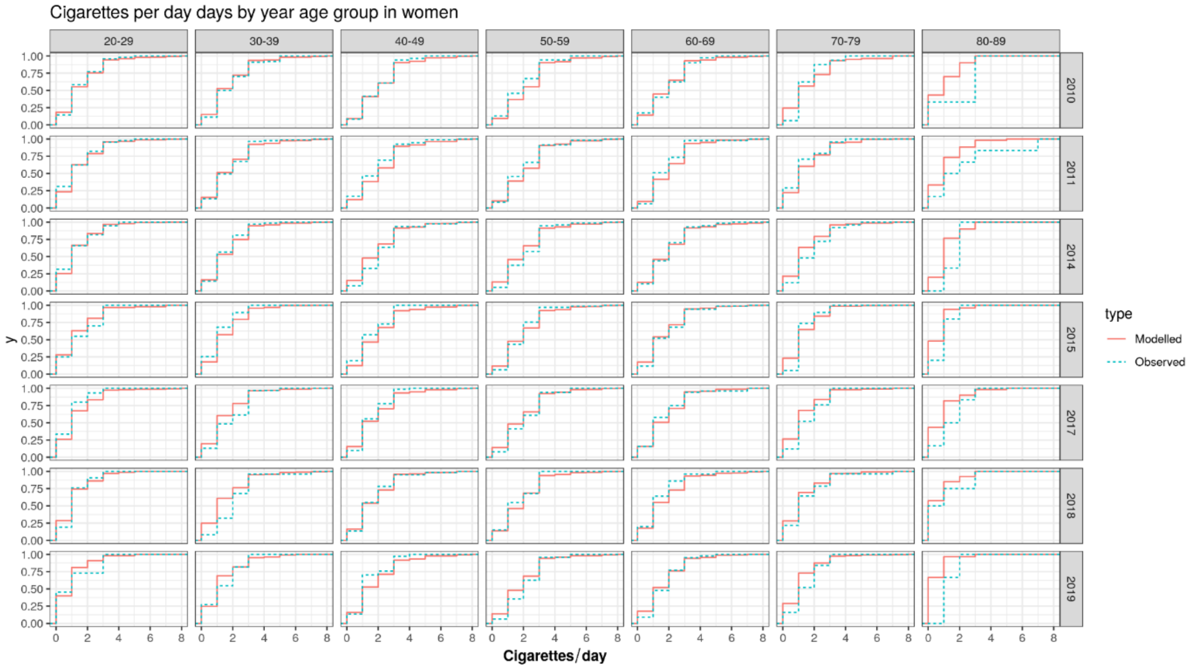

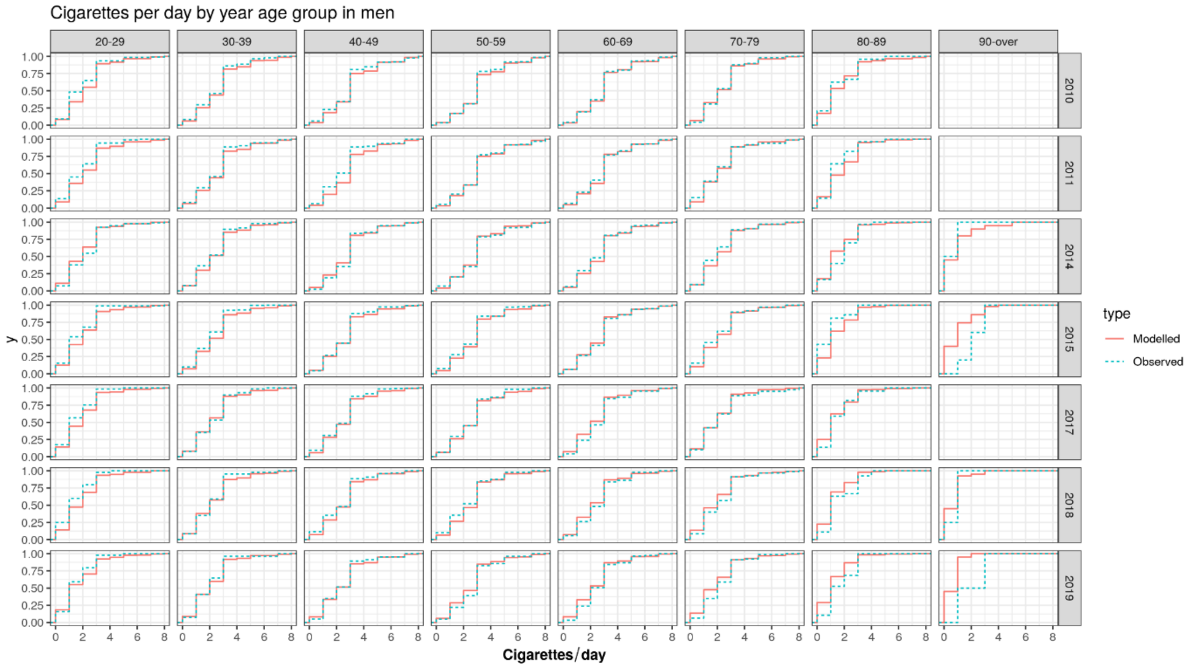

Figure B-6. Validation: Proportion of current- and never-smokers – NHNS and projected IMPACT$_{NCD\text{-}JPN}$ trends by year, age, and sex

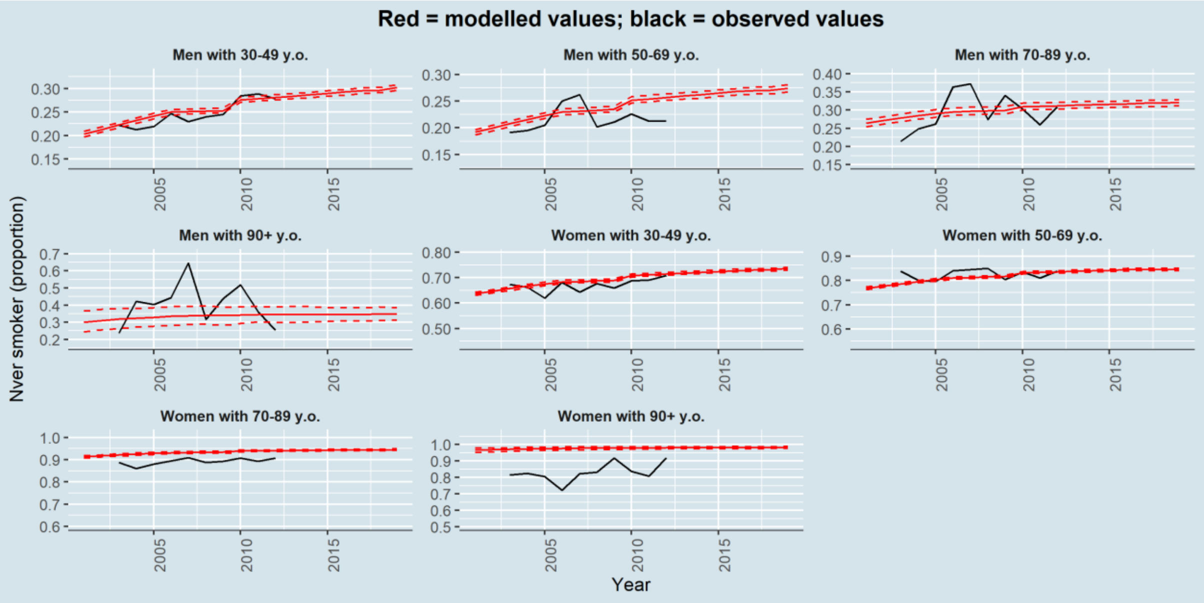

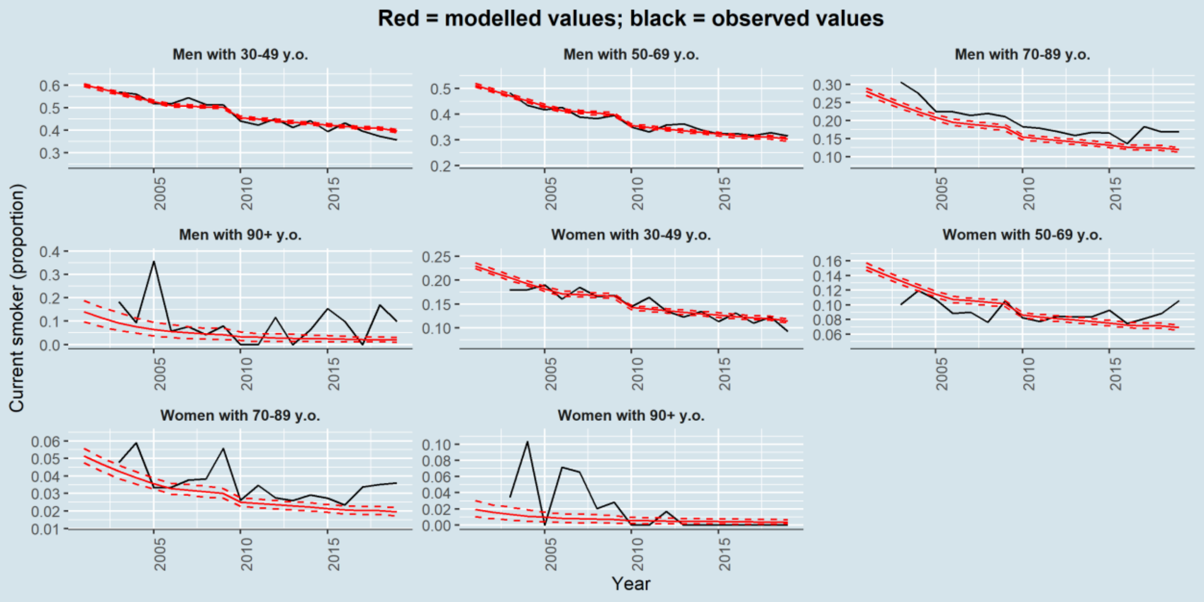

Physical Active days

Figure B-7. Validation: Active days – NHNS and projected IMPACT$_{NCD-JPN}$ trends by year, age, and sex

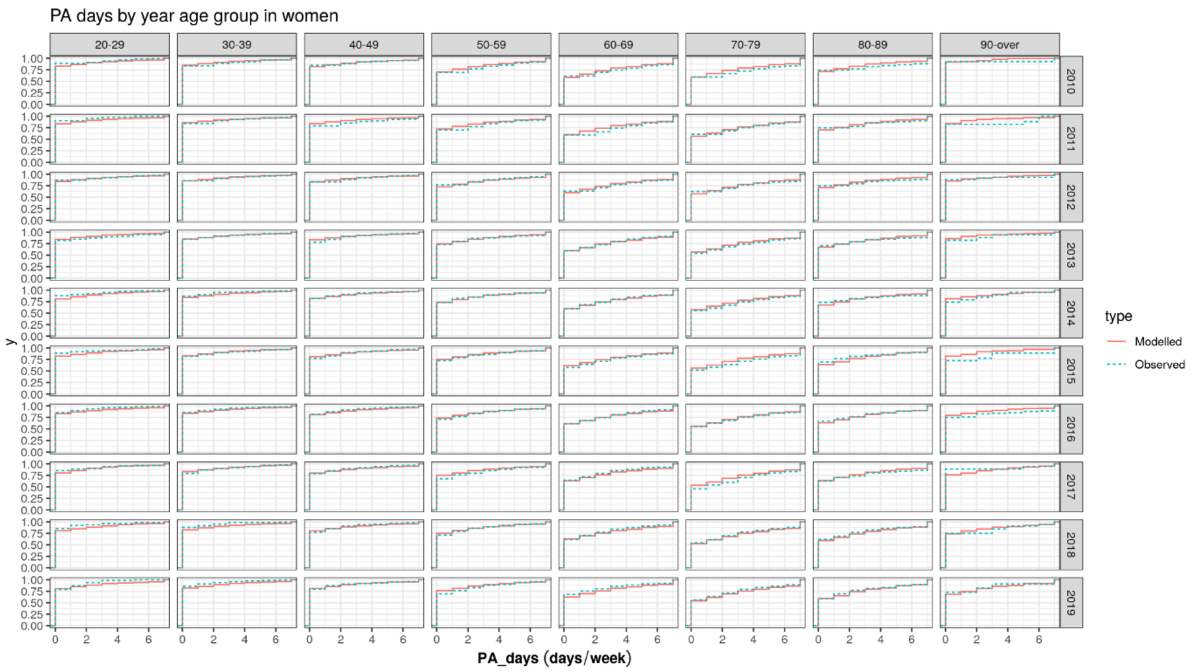

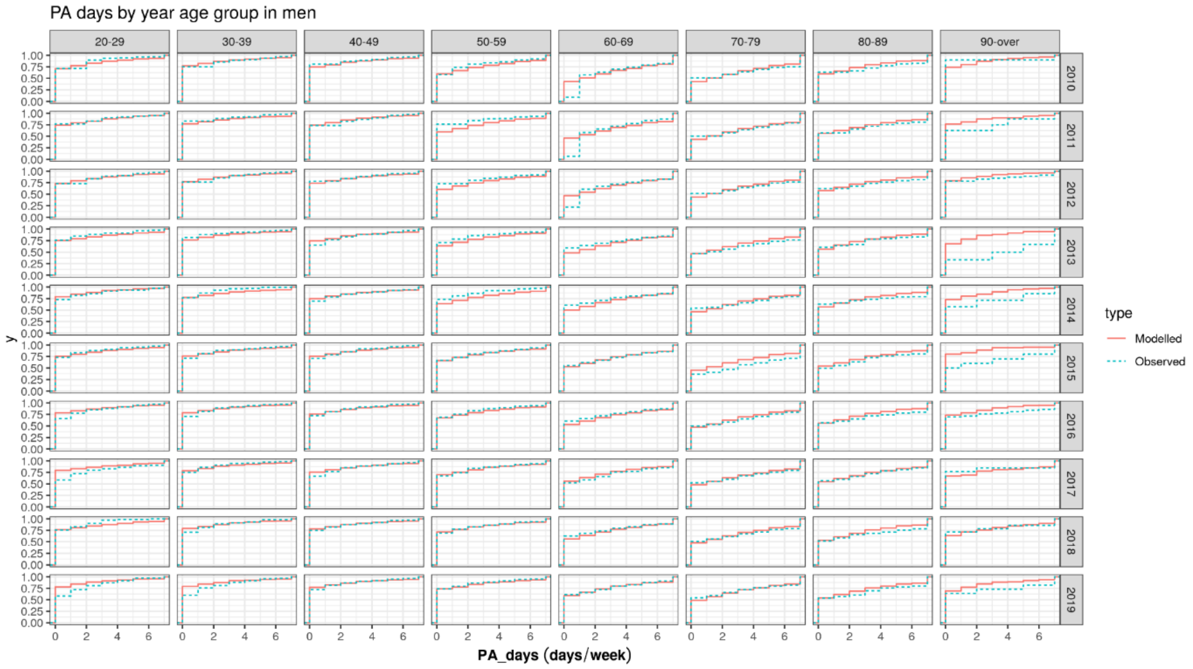

Figure B-8. Validation: Active days – NHNS and projected IMPACT$_{NCD-JPN}$ trends by year, age, and sex

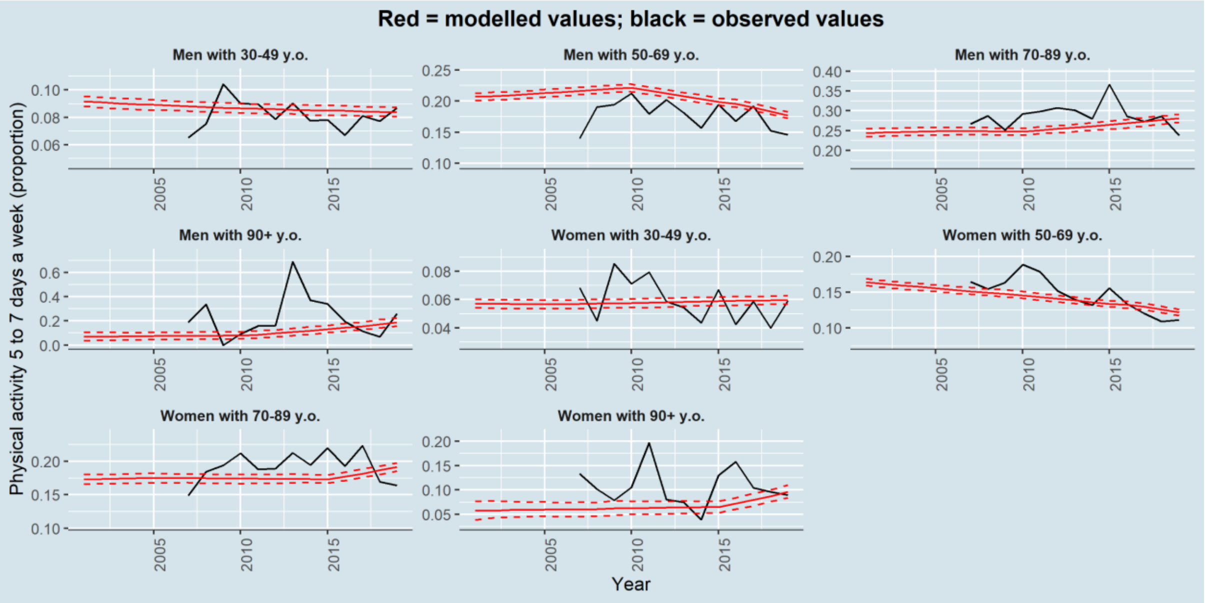

Fruit and Vegetable intake

Figure B-9. Validation: Fruit and vegetable intake – NHNS and projected IMPACT$_{NCD-JPN}$ trends by year, age, and sex

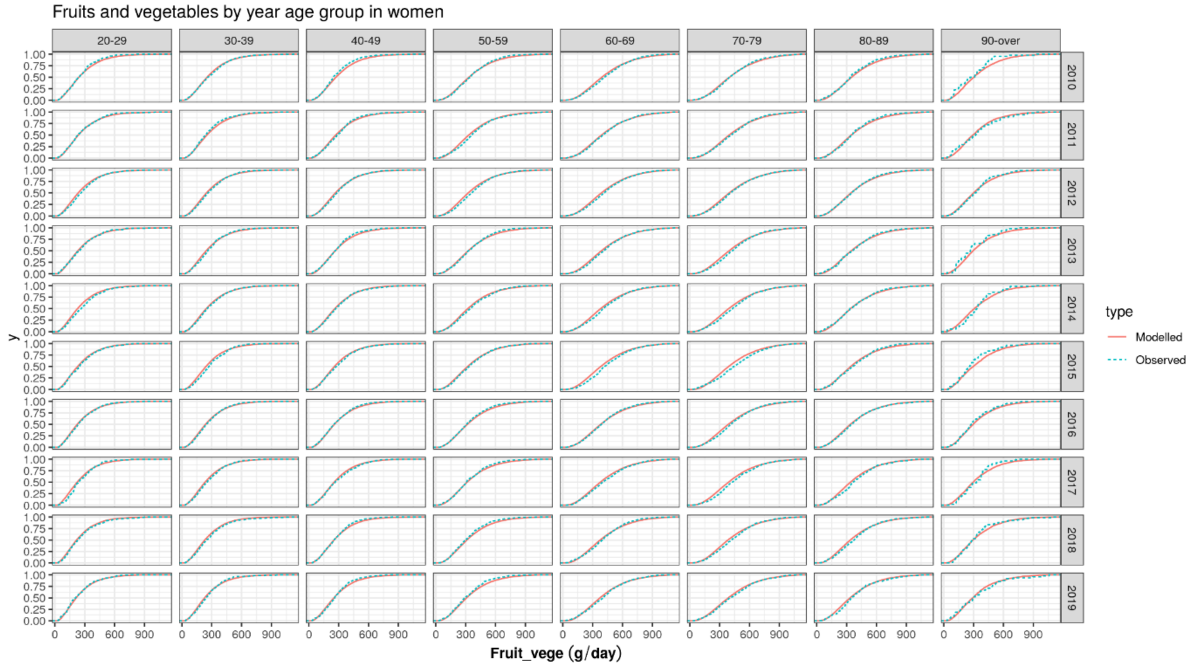

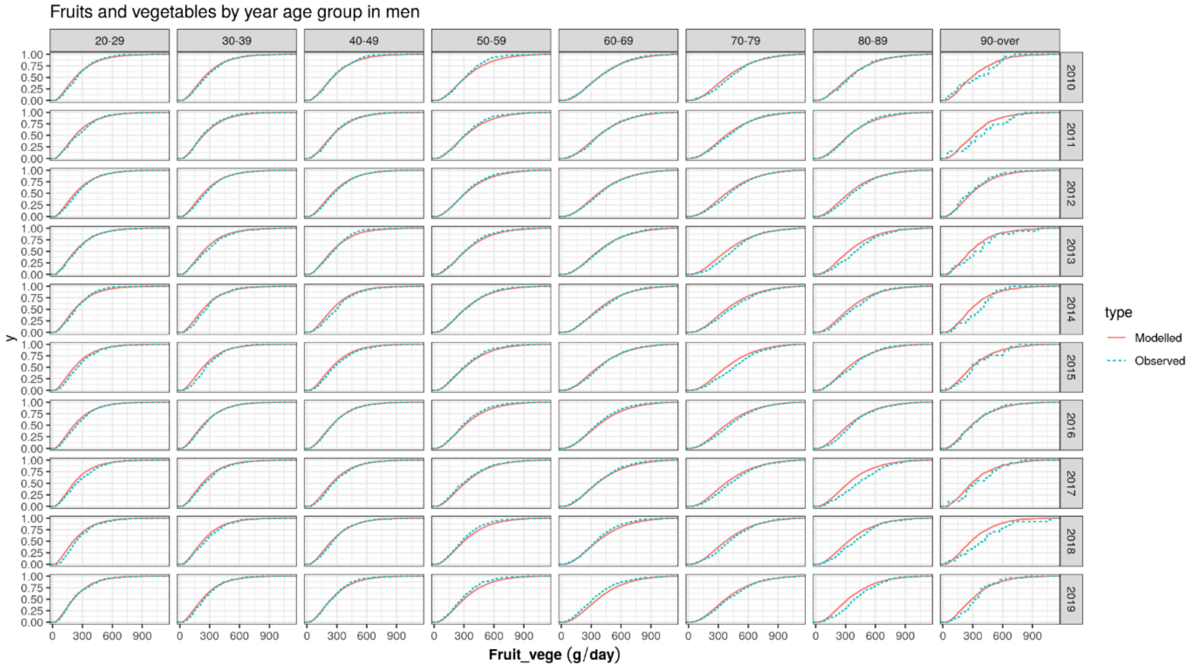

Figure B-10. Validation: Fruit and vegetable intake – NHNS and projected IMPACT$_{NCD-JPN}$ trends by year, age, and sex

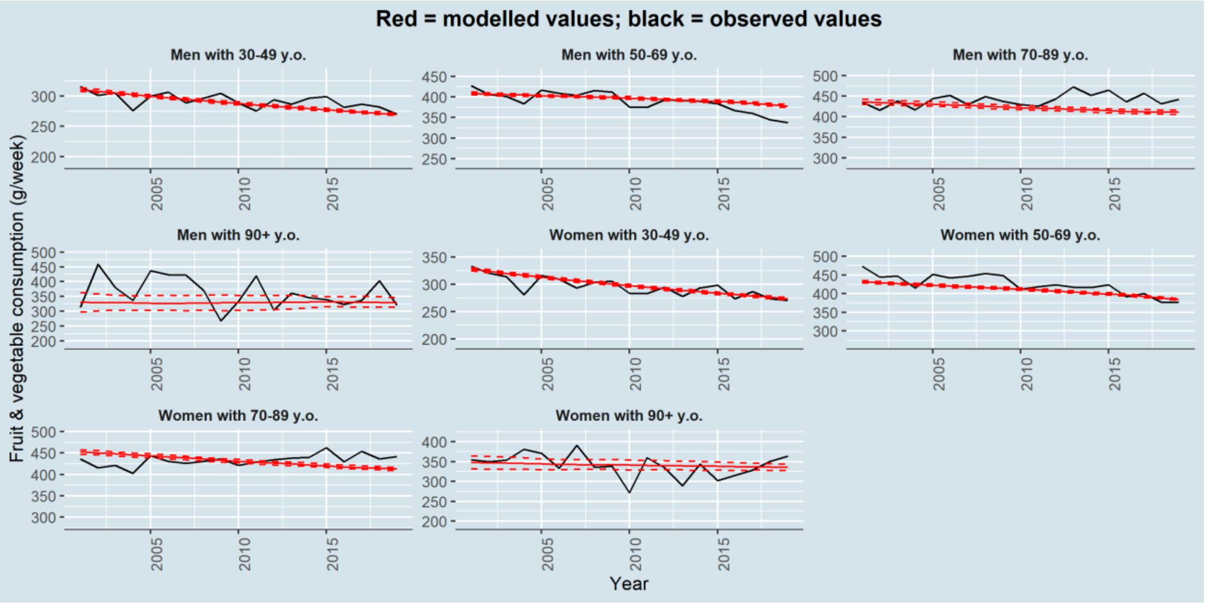

Medication use for diabetes

Figure B-11. Validation: medication use for diabetes – NHNS and projected IMPACT$_{NCD-JPN}$ trends by year, age, and sex

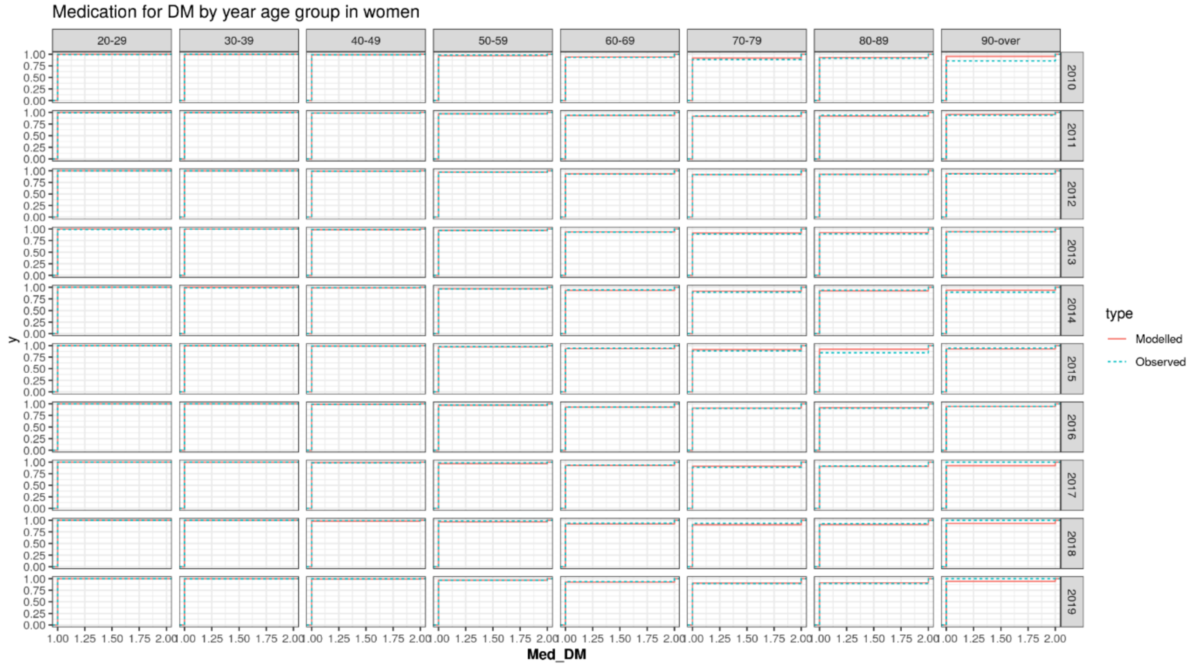

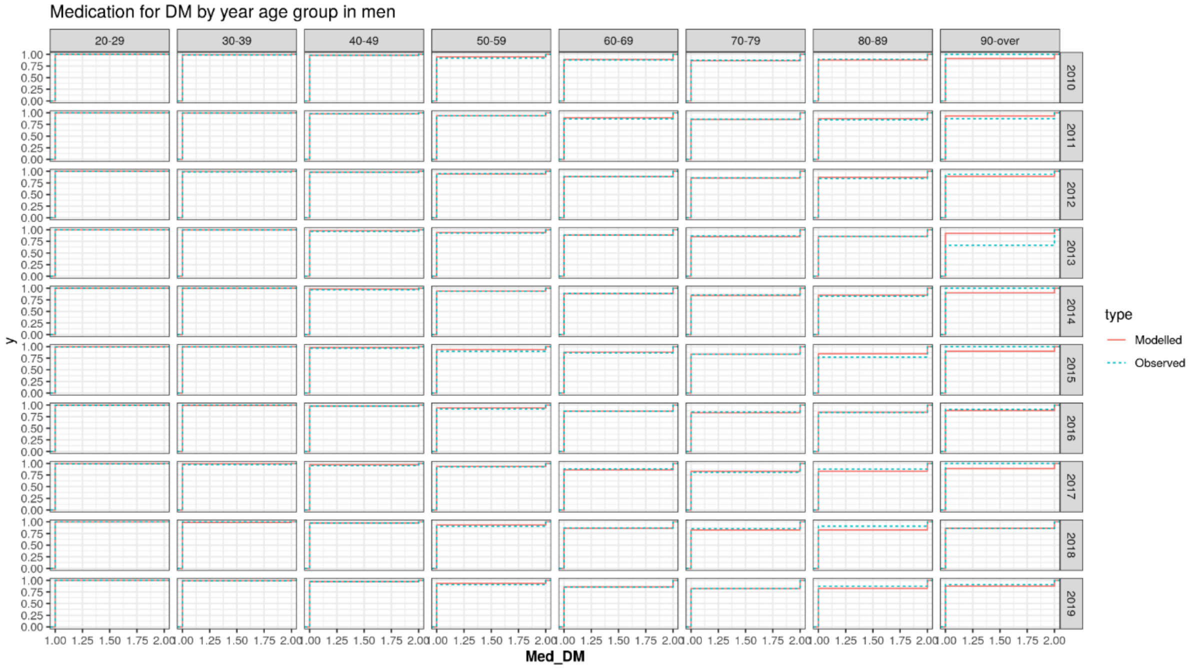

Figure B-12. Validation: medication use for diabetes – NHNS and projected IMPACT$_{NCD\text{-}JPN}$ trends by year, age, and sex

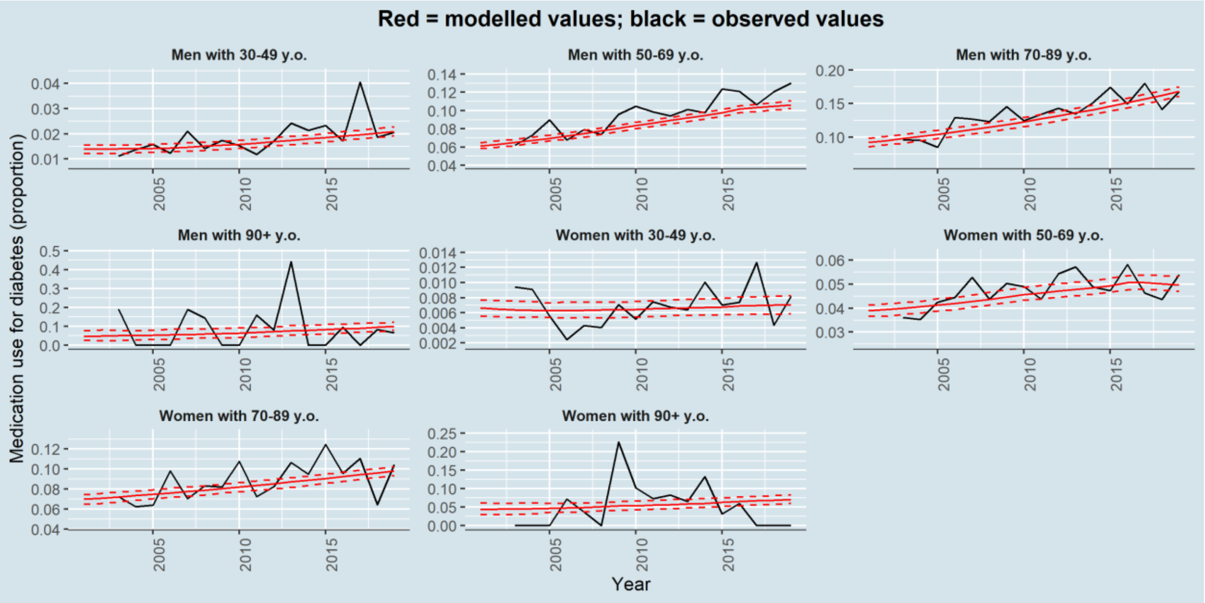

Medication use for hyperlipidemia

Figure B-13. Validation: medication use for hyperlipidemia – NHNS and projected IMPACT$_{NCD-JPN}$ trends by year, age, and sex

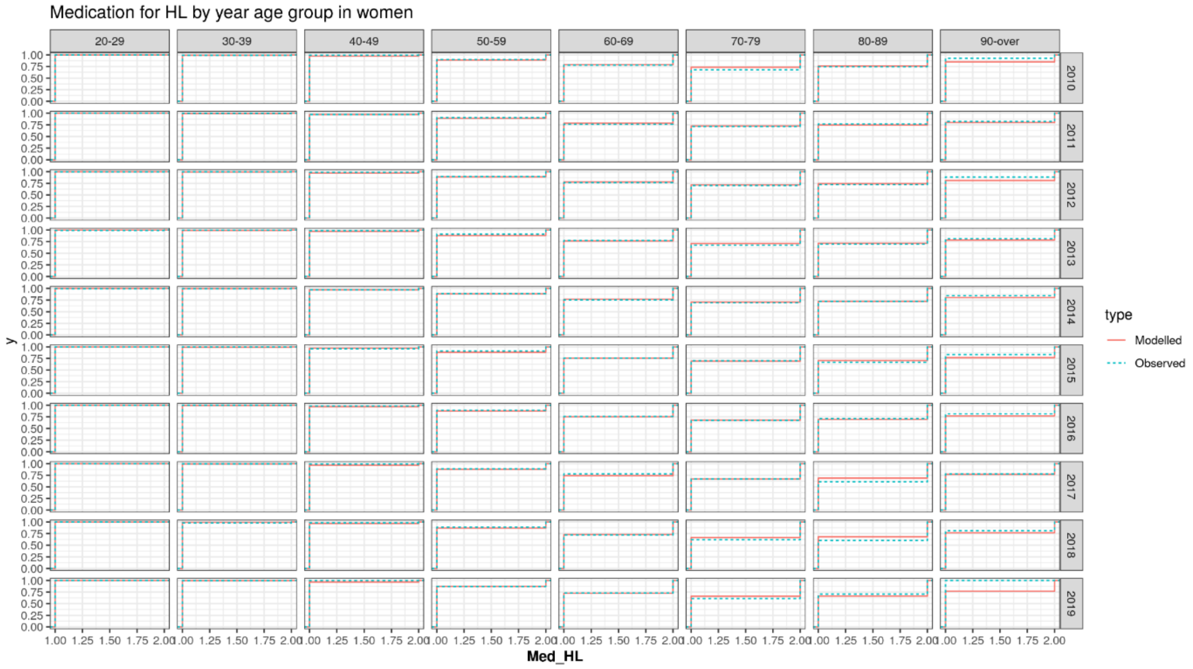

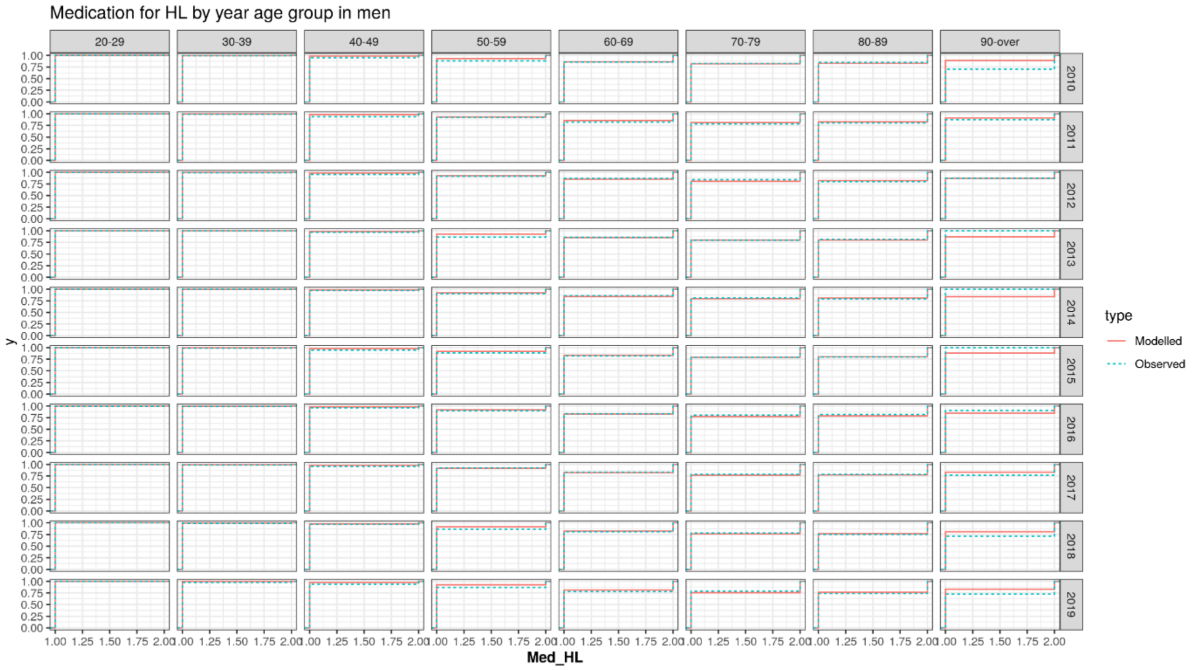

Figure B-14. Validation: medication use for hyperlipidemia – NHNS and projected IMPACT$_{NCD-JPN}$ trends by year, age, and sex

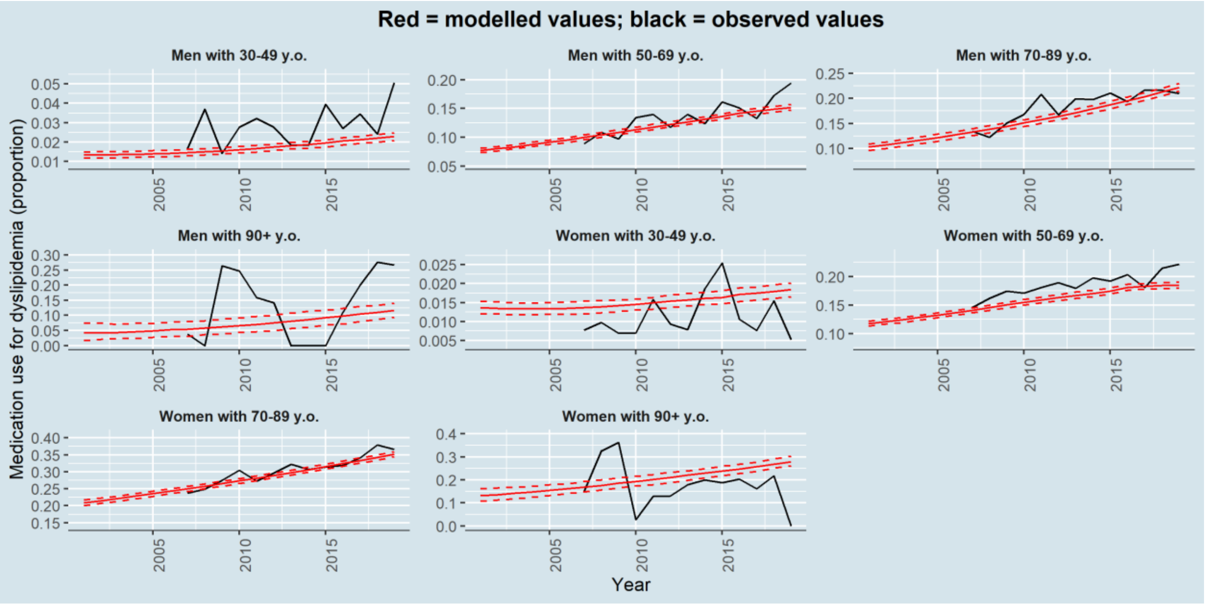

Medication use for hypertension

Figure B-15. Validation: medication use for hypertension – NHNS and projected IMPACT$_{NCD\text{-}JPN}$ trends by year, age, and sex

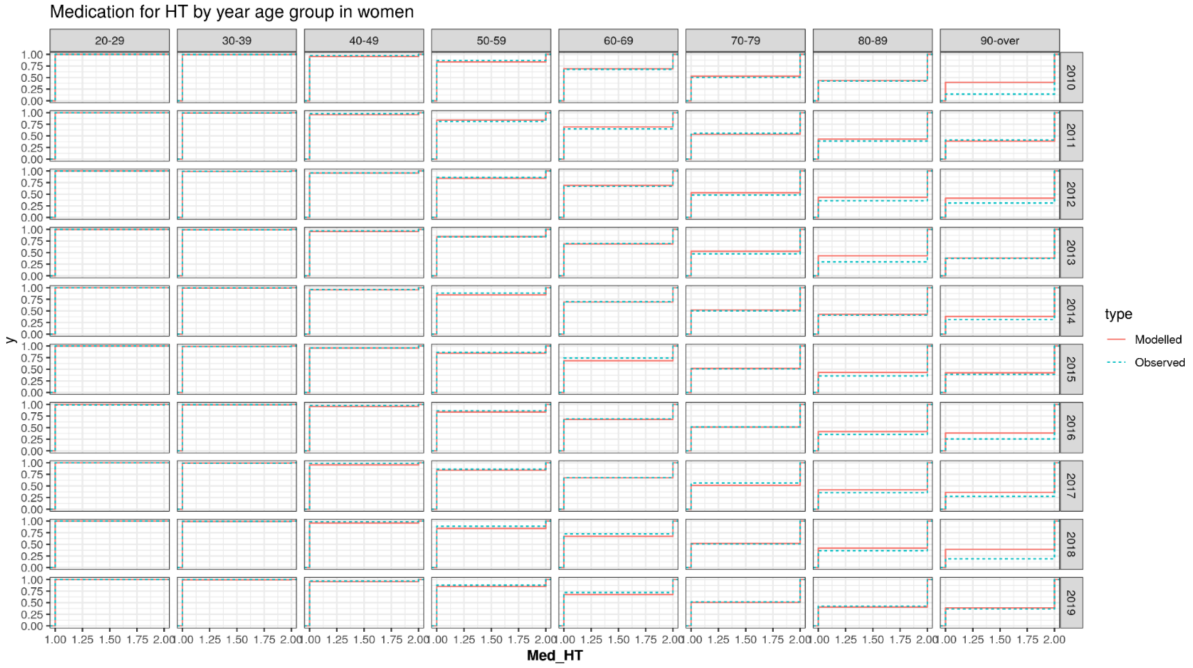

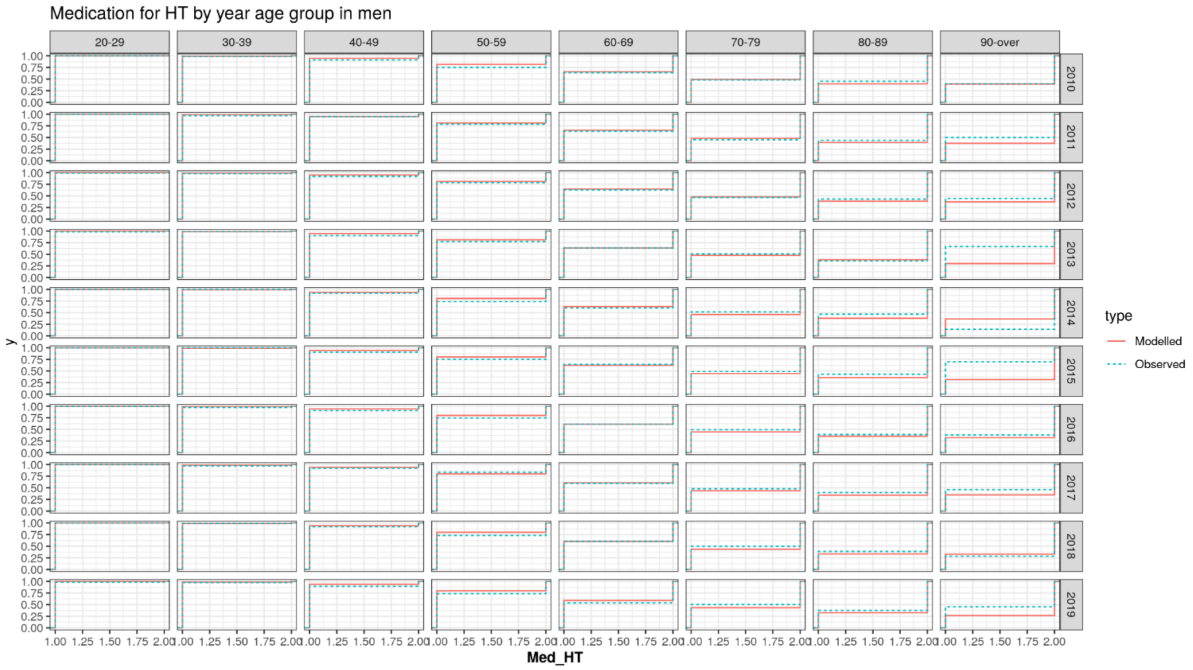

Figure B-16. Validation: medication use for hypertension – NHNS and projected IMPACT$_{NCD-JPN}$ trends by year, age, and sex

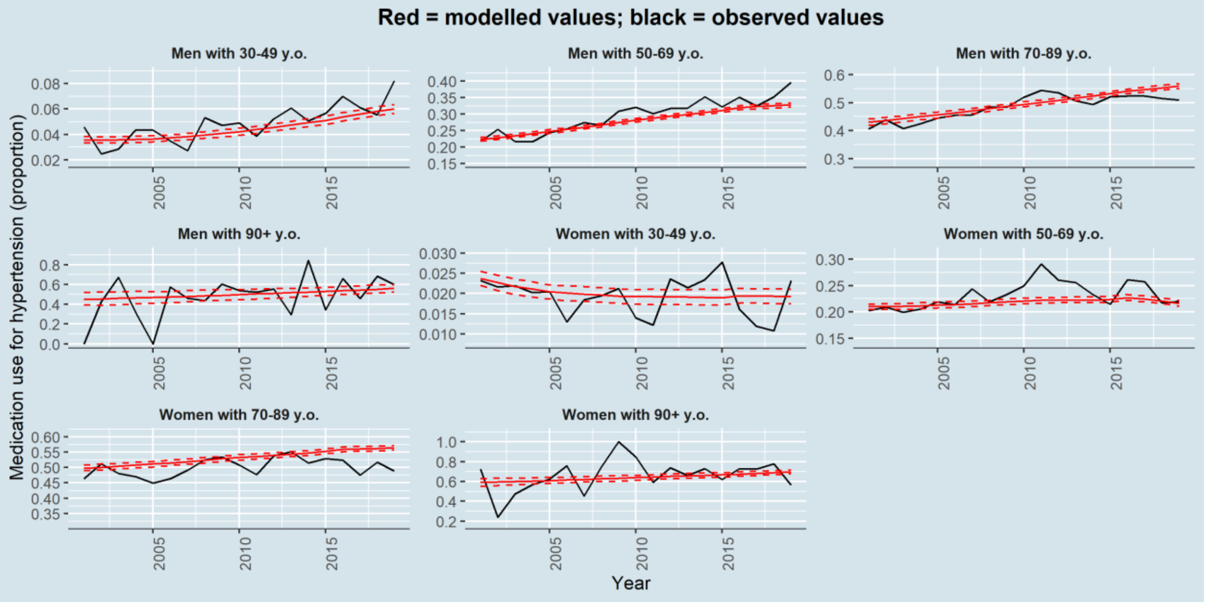

Systolic blood pressure

Figure B-17. Validation: Systolic blood pressure – NHNS and projected IMPACT$_{NCD-JPN}$ trends by year, age, and sex

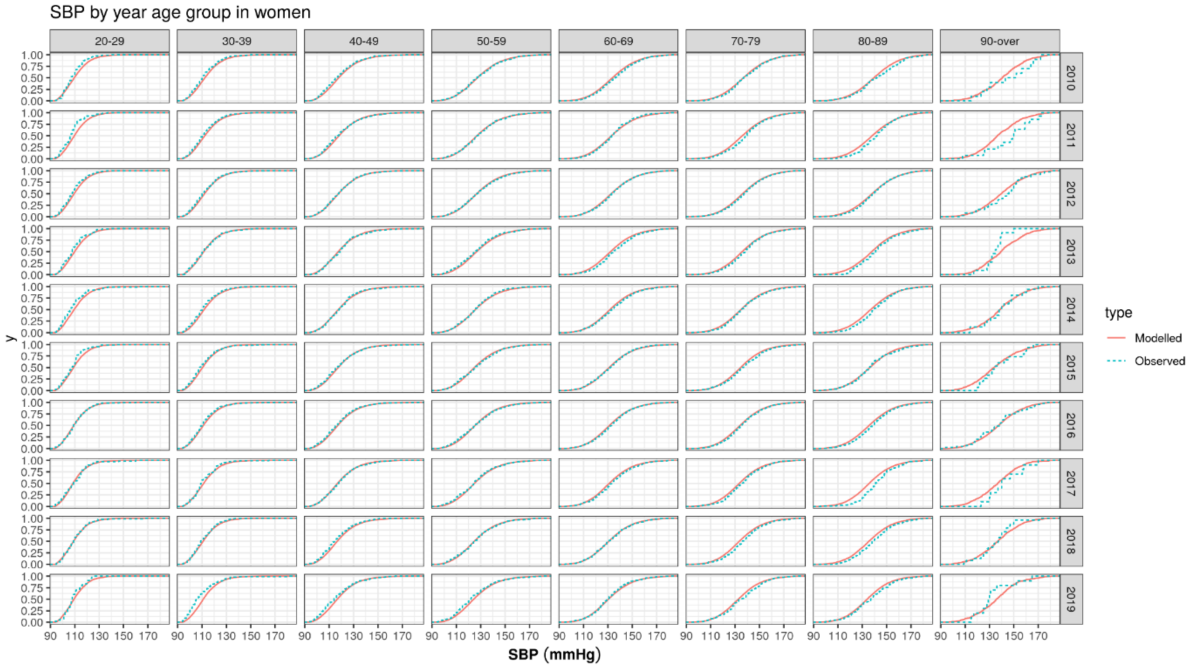

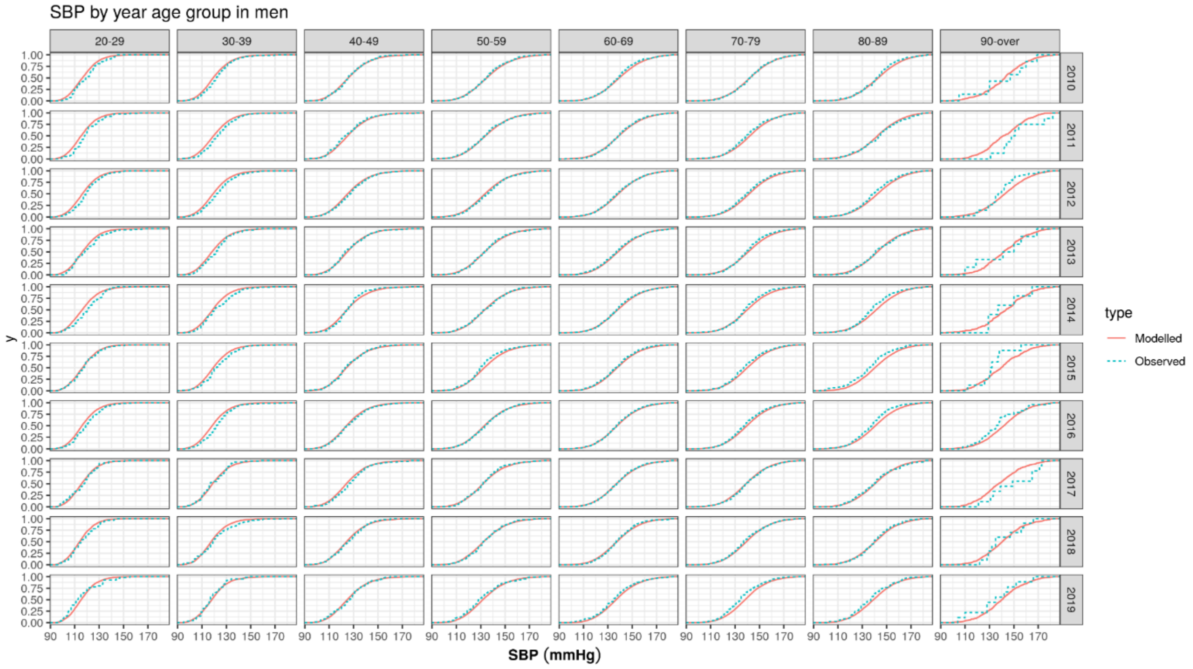

Figure B-18. Validation: Systolic blood pressure – NHNS and projected IMPACT$_{NCD-JPN}$ trends by year, age, and sex

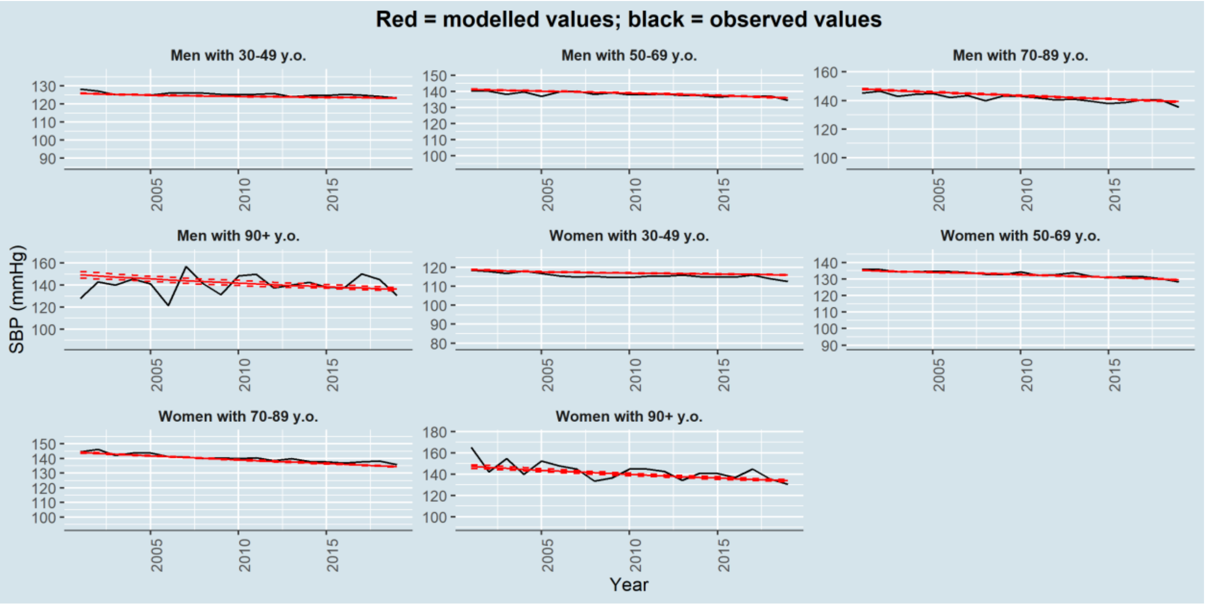

HbA1c

Figure B-19. Validation: HbA1c – NHNS and projected IMPACT$_{NCD-JPN}$ trends by year, age, and sex

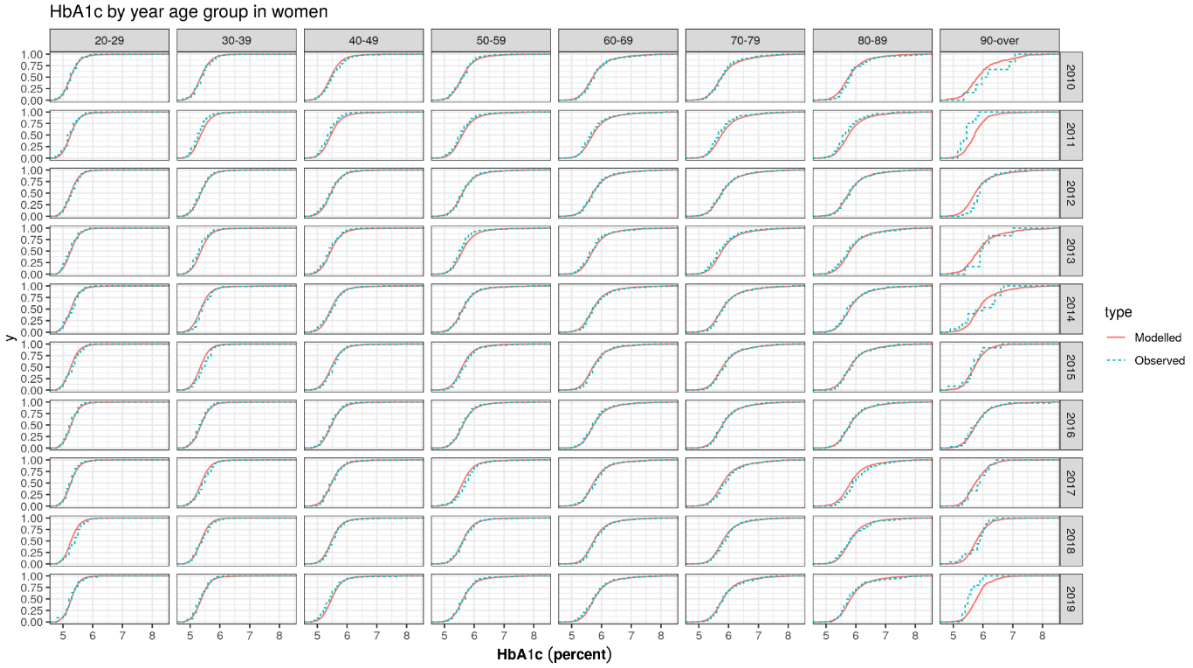

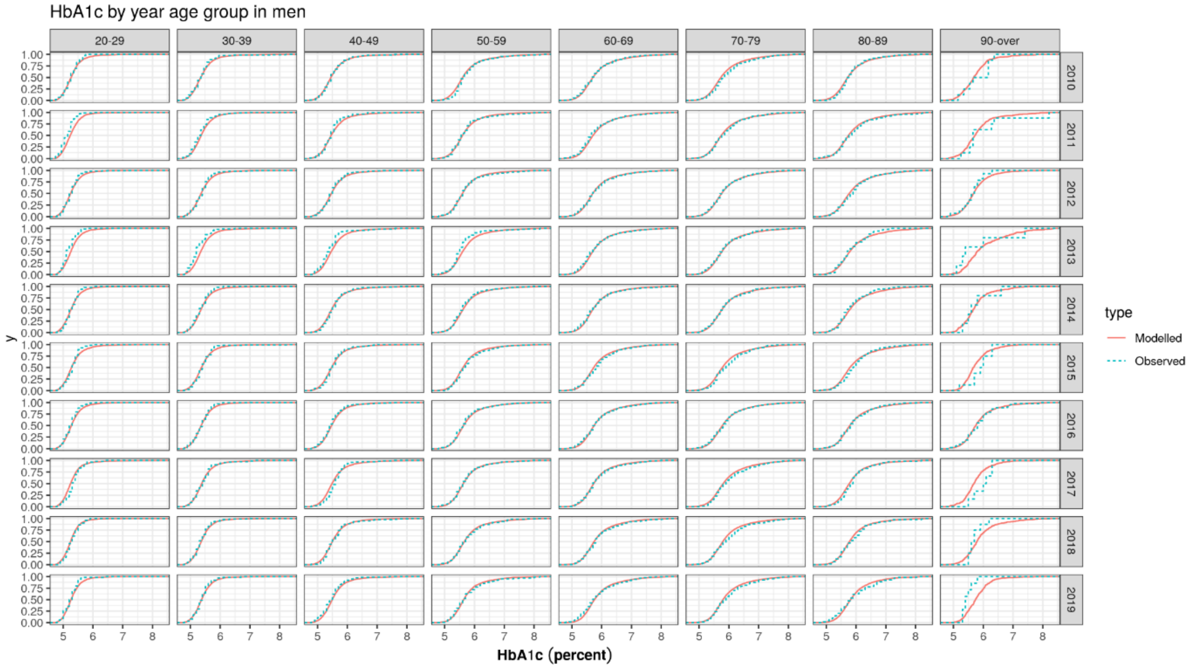

Figure B-20. Validation: HbA1c – NHNS and projected IMPACT$_{NCD-JPN}$ trends by year, age, and sex

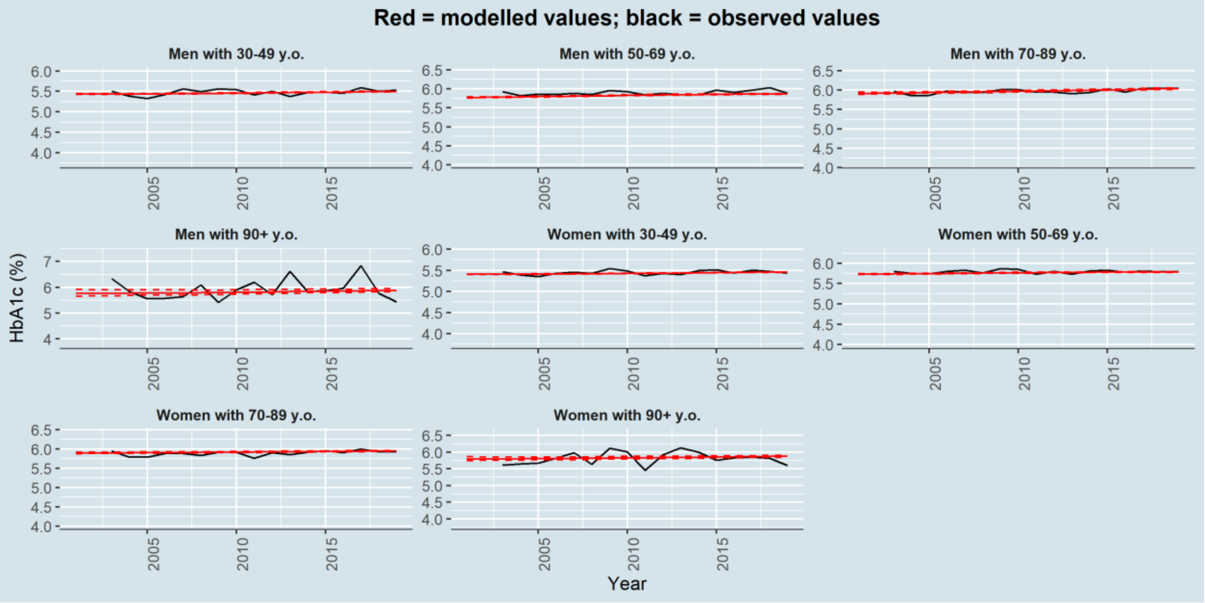

LDL-c

Figure B-21. Validation: LDL-c – NHNS and projected IMPACT$_{NCD\text{-}JPN}$ trends by year, age, and sex

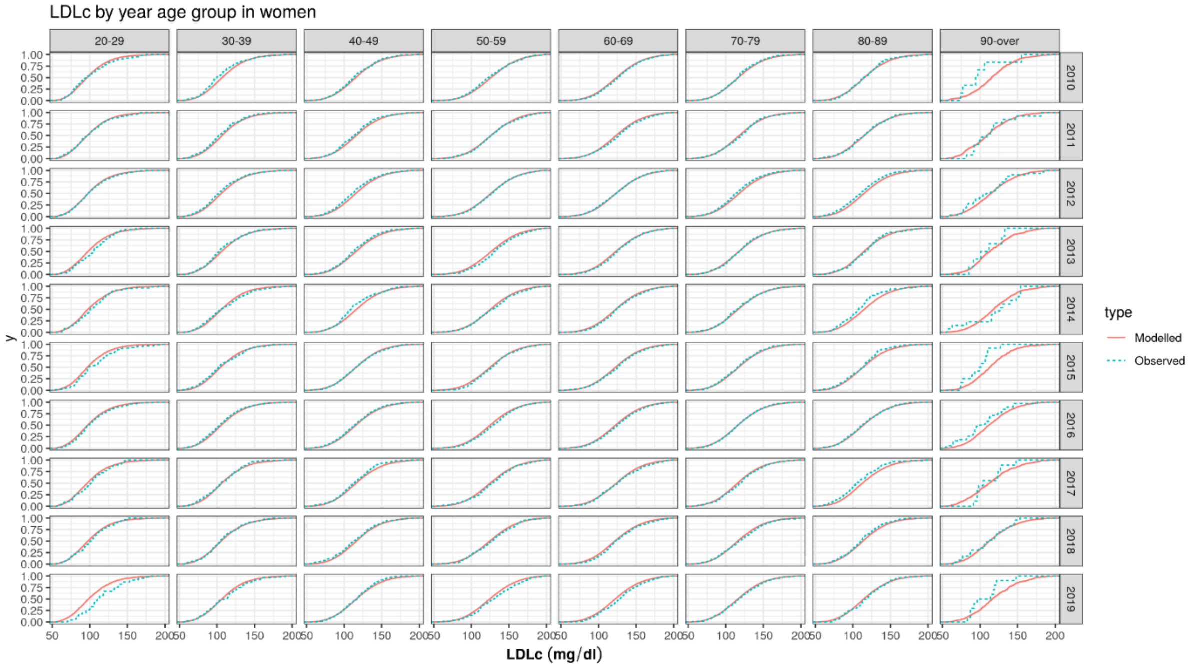

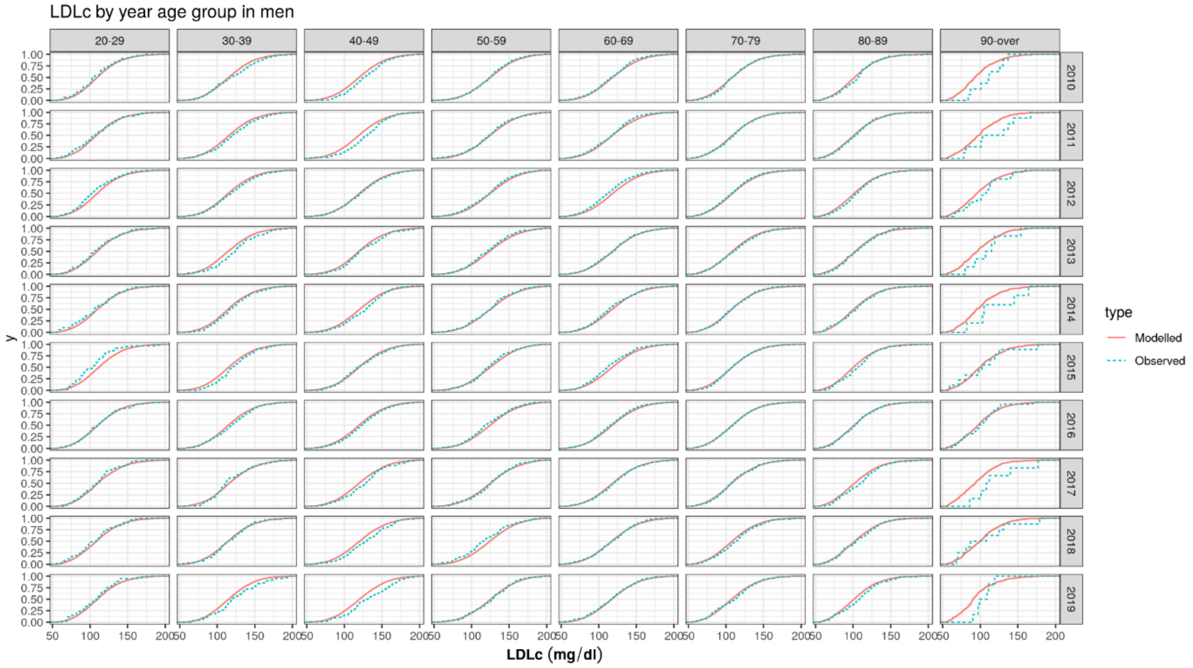

Figure B-22. Validation: LDL-c – NHNS and projected IMPACT$_{NCD\text{-}JPN}$ trends by year, age, and sex

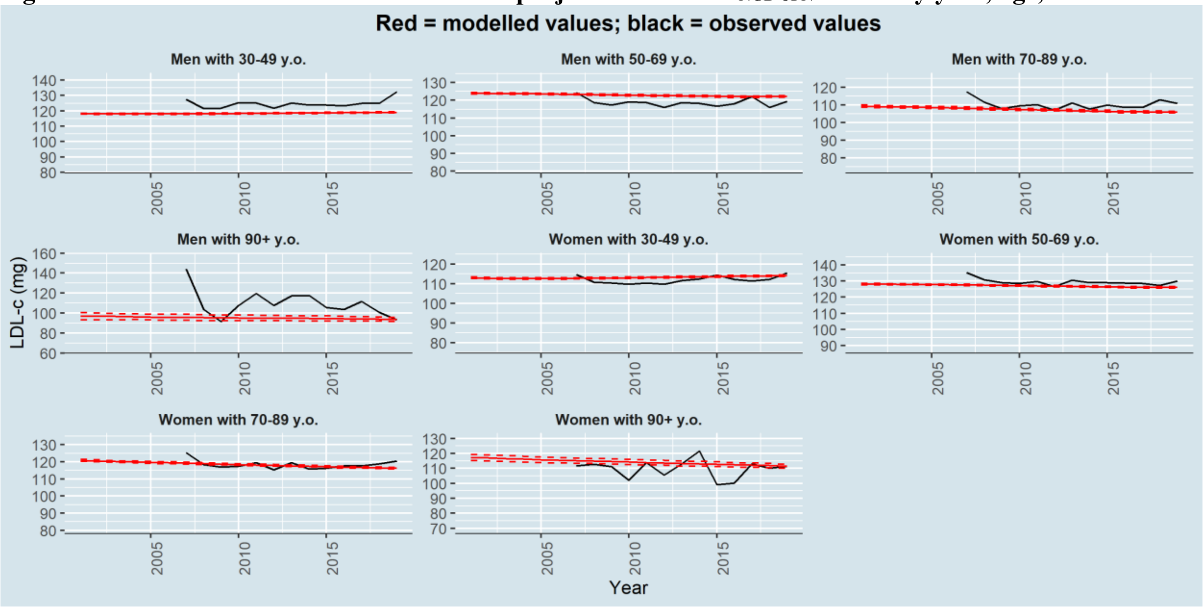

C) Supplementary Material C: Incidence rate comparisons between GBD and Japanese local registries

The $IMPACT_{NCD-JPN}$ model used GBD data for CHD and stroke incidence rates in 2001 as the initial year due to the absence of real-world national CVD morbidity data. However, the following local datasets were available: the stroke incidence rates for first-onset cases in Kochi Prefecture and the acute myocardial infarction (AMI) incidence rates, including both initial onset and recurrence, in Kumamoto Prefecture.

The current $IMPACT_{NCD-JPN}$ model aimed to quantify how changes in the national distribution of CVD risk factors contributed to the national observed trends in CVD (combined and separate for CHD and stroke) burden in Japan's adult population, leading to that we decided to use the GBD data as input data of the model. We compared 1) the stroke incidence rates from the Kochi registry with the stroke incidence estimates for Kochi from the GBD in 2019 and 2) the AMI incidence rates from the Kumamoto registry with the CHD incidence estimates for Kumamoto from the GBD.

For the stroke incidence in Kochi Prefecture, we used the Kochi Acute Stroke Survey of Onset (KATSUO) registry study conducted by the Department of Health at the Kochi Prefectural Office, which enrolled the cases of acute stroke admitted to all acute stroke hospitals in Kochi Prefecture within 7 days of symptom onset.[36]

For the AMI incidence in Kumamoto Prefecture, we used the Kumamoto Acute Coronary Events (KACE) registry study. The KACE study is a multicenter observational research project involving 21 institutions across Kumamoto Prefecture that are equipped to treat AMI patients and conduct coronary angiography and related interventions. Most AMI cases in Kumamoto Prefecture are directed to these participating facilities.[37] The KACE study registers AMI patients only, leading to much lower incidence rates across age groups compared to the CHD incidence rate in GBD.

Furthermore, we compared the incidence of GBD with that of published data from population-based registries in a similar manner above for stroke and CHD/AMI. We used the incidence of stroke from 3) Shiga Stroke Registry (Circ J 2017; 81: 1636-46.); and the incidence of AMI from 4) Shiga Stroke and Heart Attack Registry (J Atheroscler Thromb 2023; 30: 1407-19). Both are population-based registries that cover approximately 1.4 million residents of Shiga Prefecture.

(1)

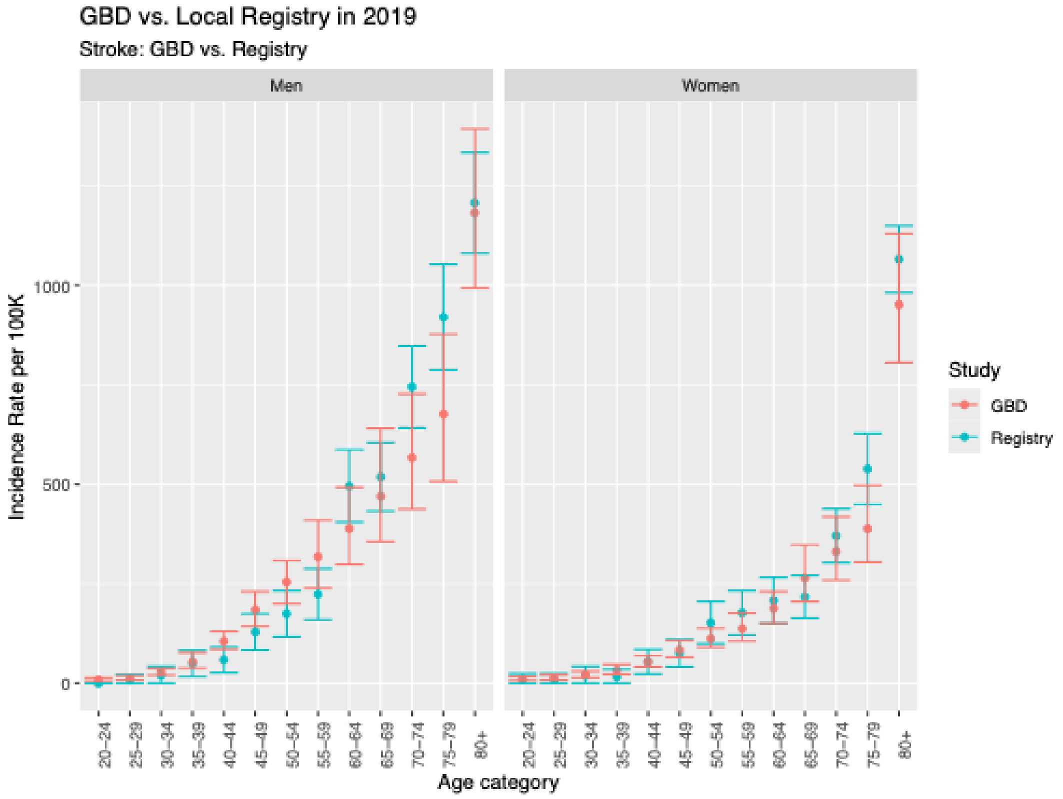

(2)

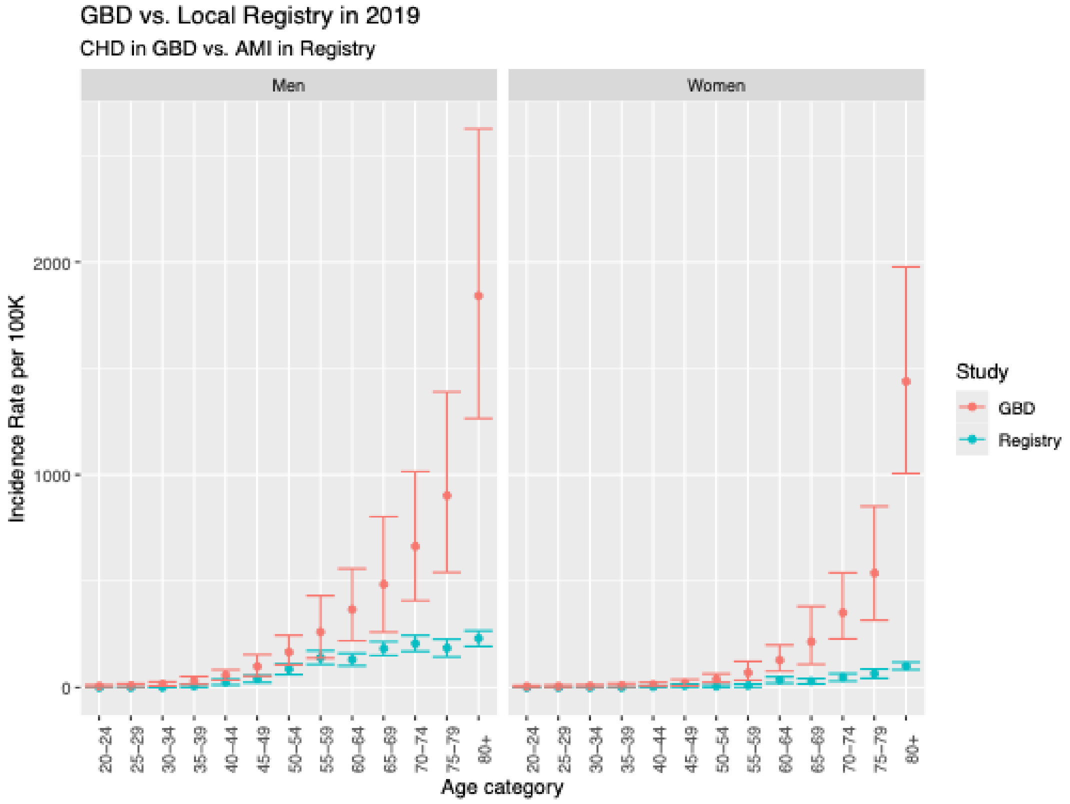

Note: The local registry registered AMI patients only, leading to a much lower incidence rate than the CHD incidence rate in GBD.

(3)

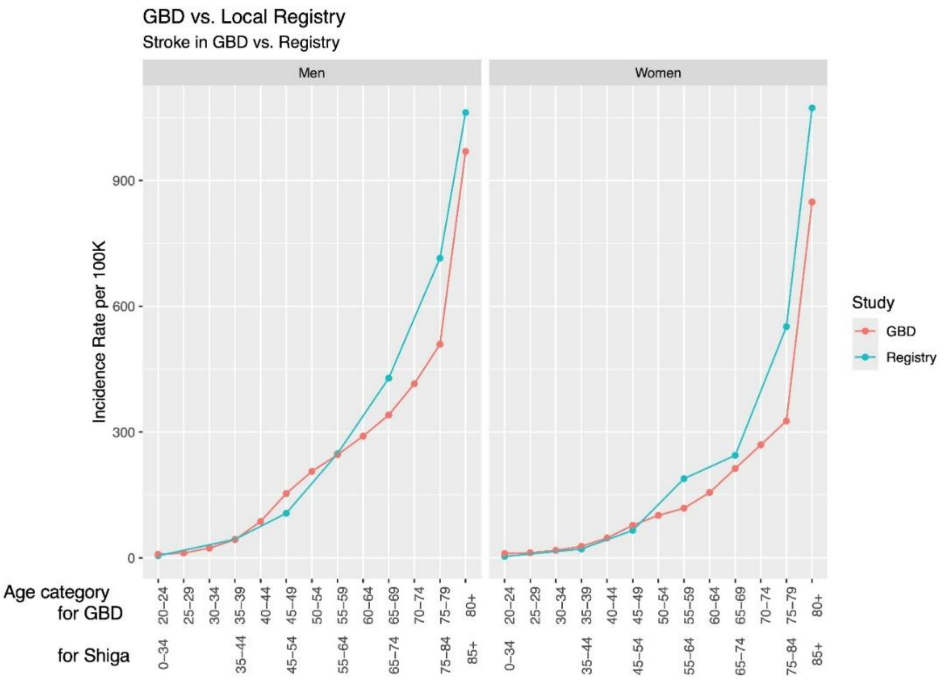

(4)

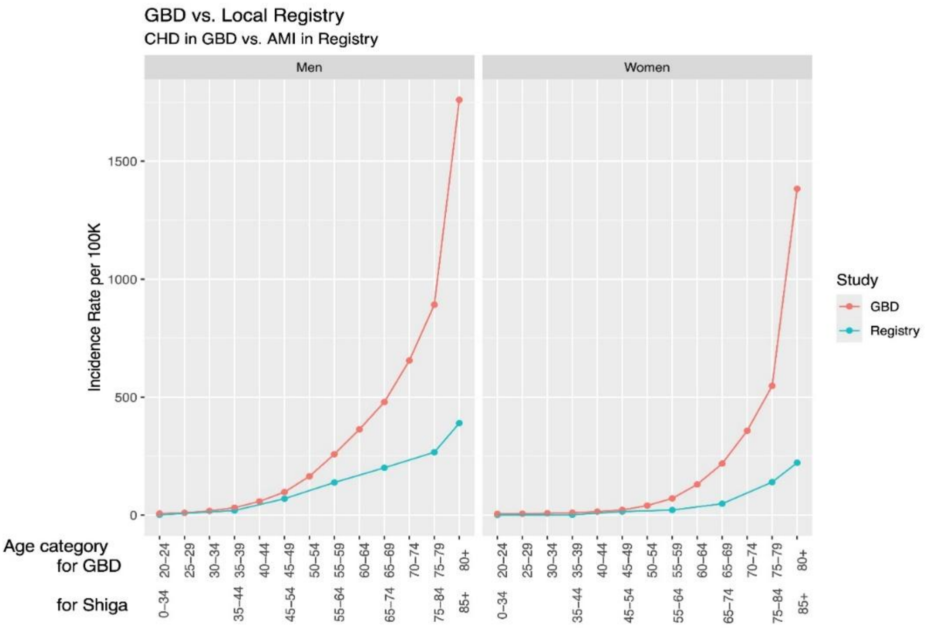

Note: The local registry registered AMI patients only, leading to a much lower incidence rate than the CHD incidence rate in GBD.

D) Supplementary Material D: Consideration of the transition from standard mercury sphygmomanometers to mercury-free devices in the 2019 National Health and Nutrition Survey

In 2019, the National Health and Nutrition Survey (NHNS) transitioned from mercury sphygmomanometers to certified mercury-free auscultatory devices. Specifically, the models used were the A&D UM-102 or UM-102B, which are updated versions of the previously validated UM-101. These updates include differences in external housing materials but retain the core measurement principles and design, such as the auscultatory method and manual inflation. The Ministry of Health, Labour and Welfare selected these devices based on four criteria to ensure continuity with mercury-based readings: (i) use of the auscultatory method, (ii) manual inflation, (iii) bar display mimicking mercury columns, and (iv) certification as medical devices.

While no direct validation study has yet been published for the UM-102/102B in the NHNS setting, the predecessor model (UM-101) has undergone multiple peer-reviewed validation studies. Stergiou et al. (2008) demonstrated that the UM-101 met ESH International Protocol standards when used without the "mark" button, with 87%, 97%, and 99% of SBP readings falling within 5, 10, and 15 mmHg of reference values.[1] Use of the "mark" button, however, resulted in increased bias and failed to meet the protocol criteria.[1] Additionally, Pruijm et al. (2010) confirmed the device's accuracy, finding 86%, 96%, and 99% (SBP) and 94%, 99%, 99% (DBP) of readings, respectively, within 5, 10, and 15 mmHg of mercury standard measurements.[2] Davis et al. (2015) validated the UM-101 in pregnant women, where it outperformed an automated device and achieved an A/A rating under the BHS protocol (94% for SBP and 97% for DBP within 5 mmHg).[3]

The NHNS 2019 protocol did not specify whether the "mark" button was used during data collection, introducing some uncertainty. Nonetheless, several factors suggest that any impact on the study's conclusions is minimal: (1) similar interannual SBP variation was observed prior to 2019, (2) the change occurred only in the final year of a 19-year period, and (3) our regression-based modelling approach by generalized additive models for location, scale, and shape (GAMLSS) smooths across adjacent years, which is robust to such localised variation. The Figure D-1 in the Supplementary Material D presents annual SBP values and year-on-year SBP changes throughout the study period.

In summary, although the introduction of a new measurement device introduces a minor methodological limitation, the available evidence suggests it does not meaningfully affect the conclusions of the study.

1. Stergiou GS, Giovas PP, Gkinos CP, Tzamouranis DG. Validation of the A&D UM-101 professional hybrid device for office blood pressure measurement according to the International Protocol. Blood Press Monit. 2008 Feb;13(1):37-42. doi: 10.1097/MBP.0b013e3282c9acb0. PMID: 18199922.
2. Pruijm MT, Wuerzner G, Glatz N, Alwan H, Ponte B, Ackermann D, Burnier M, Bochud M. A new technique for simultaneous validation of two manual nonmercury auscultatory sphygmomanometers (A&D UM-101 and Accoson Greenlight 300) based on the International protocol. Blood Press Monit. 2010 Dec;15(6):322-5. doi: 10.1097/MBP.0b013e32833f56a8. PMID: 20827175.
3. Davis GK, Roberts LM, Mangos GJ, Brown MA. Comparisons of auscultatory hybrid and automated sphygmomanometers with mercury sphygmomanometry in hypertensive and normotensive pregnant women: parallel validation studies. J Hypertens. 2015 Mar;33(3):499-505; discussion 505-6. doi: 10.1097/HJH.0000000000000420. PMID: 25380148.

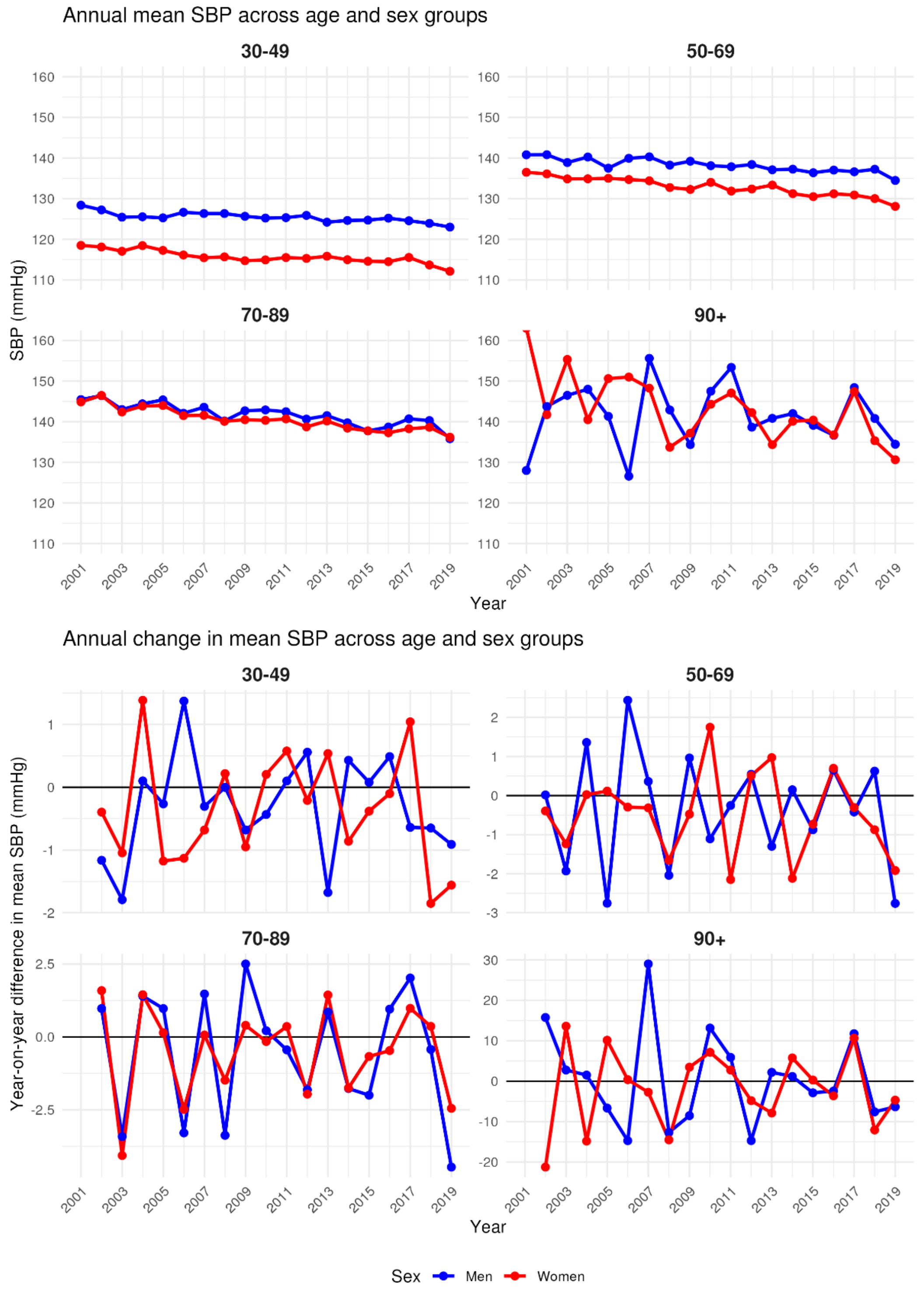

Figure D-1. Annual SBP values and year-on-year SBP changes across age and sex groups observed in NHNS between 2001 and 2019.

Abbreviations: SBP, systolic blood pressure; NHNS, National Health and Nutrition Survey

Supplementary Results

Supplementary Figures

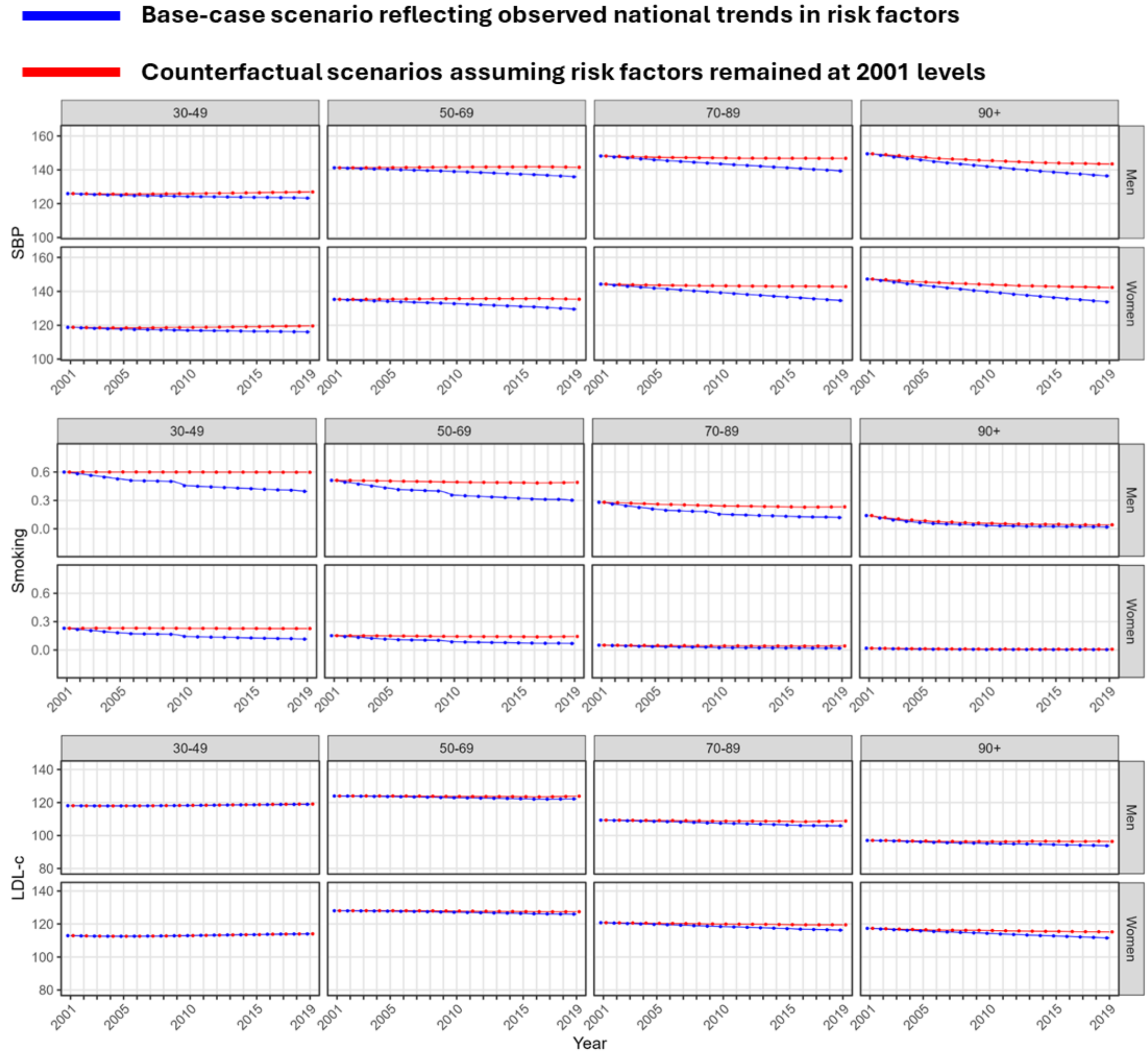

Supplementary Figure S1. Trends in modelled risk factors in Japan between 2001 and 2019 under a base-case scenario reflecting observed national trends and counterfactual scenarios assuming no change from 2001 levels, modelled by IMPACT$_{\text{NCD-JPN}}$ (means or proportions by sex and age group).

Abbreviations: CVD, cardiovascular disease; SBP, systolic blood pressure; LDL-c, low-density lipoprotein cholesterol; BMI, body mass index; PA, physical activity; FV, fruit and vegetable; GAMLSS, Generalized Additive Models for Location, Scale and Shape.

Trends in seven CVD risk factors were modelled by sex and age group under two scenarios: (1) a base-case reflecting observed national trends, and (2) counterfactual scenarios assuming 2001 levels remained unchanged. IMPACT$_{\text{NCD-JPN}}$ simulated synthetic individuals by sex, single-year age, year, and risk factors (SBP [mmHg], smoking [%], LDL-c [mg/dL], HbA1c [%], BMI [kg/m^2], PA [% active 5–7 days/week], FV [g/day]). Each risk factor was modelled by age, sex, year, and, where applicable, selected relevant CVD risk factors. Due to interdependencies and demographic shifts, counterfactual values may slightly differ from 2001. See ‘Demographic module’, ‘Exposure module’, and ‘Clustering of risk factors’ in the Supplementary Methods.

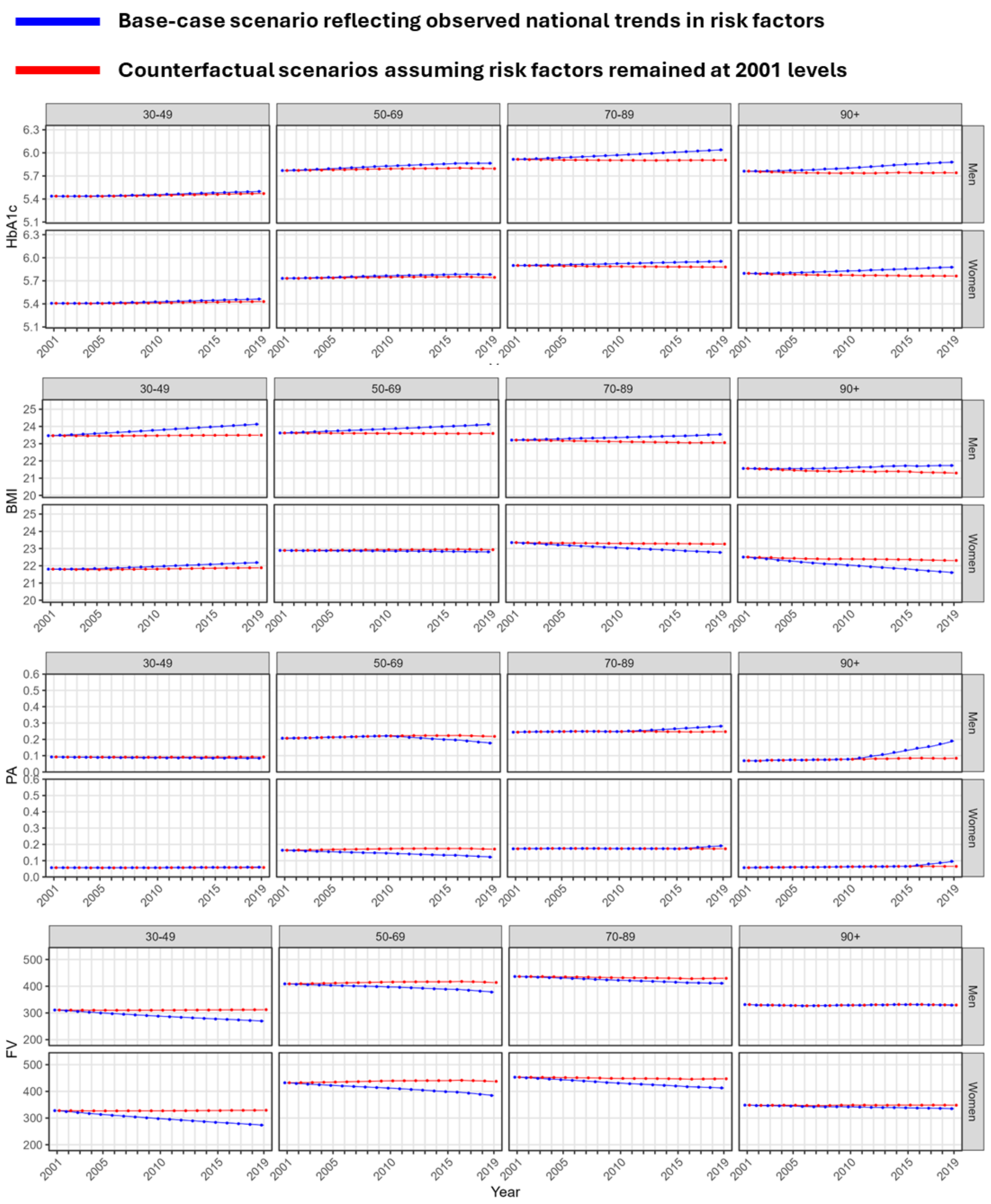

Supplementary Figure S1 (continued).

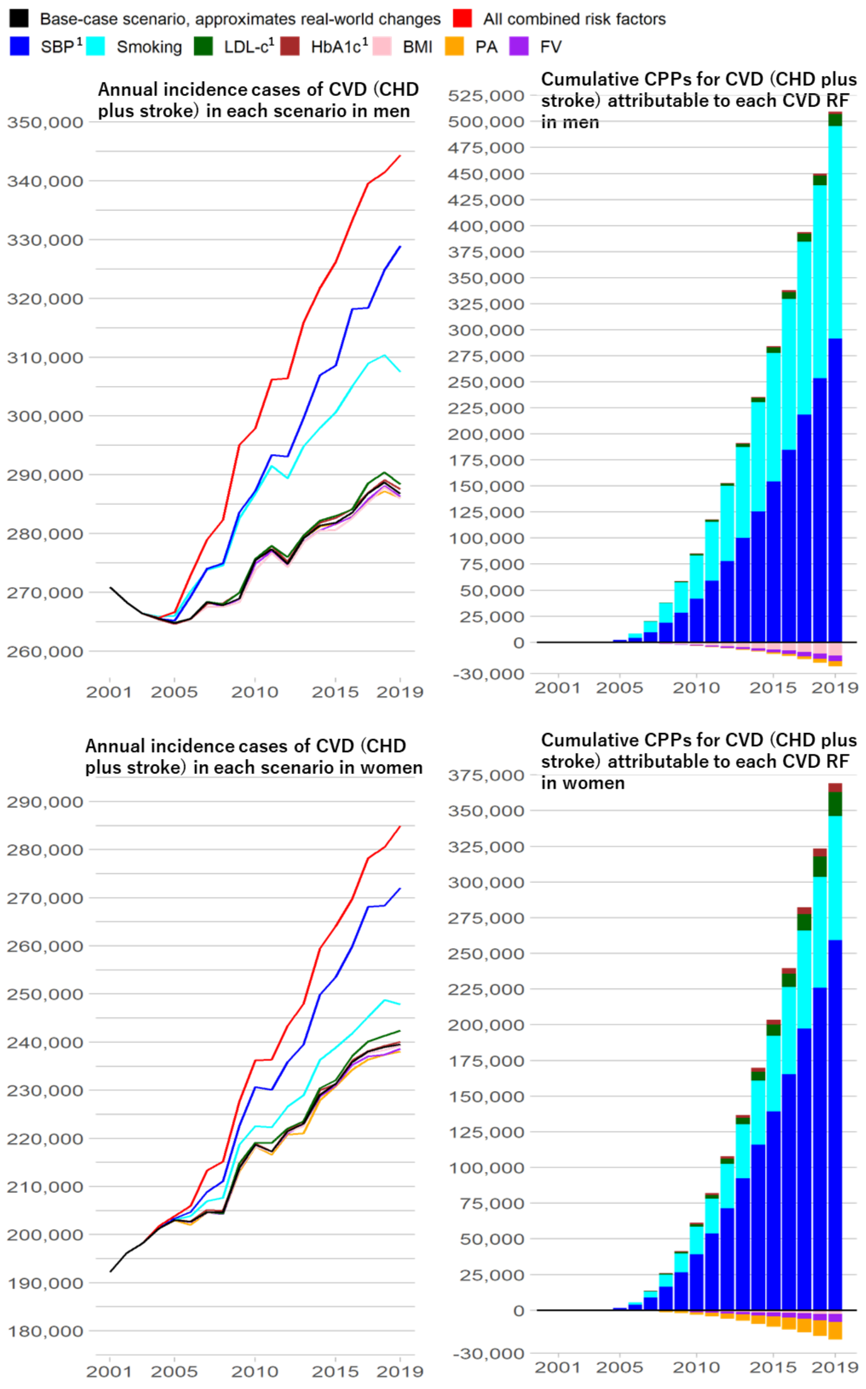

Suppl Supplementary Figure S2. Annual incidence of CVD (CHD plus stroke), and their cumulative CPPs from 2001 to 2019 in Japan, under the base-case scenario and counterfactual scenarios where each modelled CVD risk factor, and all combined, are fixed at 2001 levels.

Abbreviations: CHD, coronary heart disease; CVD, cardiovascular disease; PA, physical activity; FV, fruit and vegetable consumption; BMI, body mass index; LDL-c, low-density lipoprotein cholesterol; SBP, systolic blood pressure; CPPs, cases prevented or postponed.

[1] SBP, LDL-c, and HbA1c were adjusted for medication use of antihypertensive, cholesterol-lowering, and diabetes treatments, respectively.

Supplementary Tables

Supplementary Table S1. Characteristics (with 95% UI) for CVD (CHD plus stroke) in 2001 and 2019 in Japan, estimated in the disease and health economics module of IMPACT$_{NCD-JPN}$ in the base-case scenario, aligned closely with real-world evidence.

	**Men**		**Women**	
**Variables**	**2001**	**2019**	**2001**	**2019**
**CVD**				
Crude incidence (per 100,000)	680 (460 to 1,100)	650 (500 to 960)	440 (330 to 730)	500 (380 to 730)
Crude prevalence (per 100,000)	6,800 (6,100 to 7,400)	9,400 (7,500 to 13,000)	4,000 (3,600 to 4,400)	5,900 (4,900 to 8,200)
Direct costs (in USD)	9.8B (7.1B to 12B)	15B (not applicable)	6.8B (5.3B to 8.1B)	12B (not applicable)
Indirect costs (in USD)	56B (41B to 78B)	46B (37B to 55B)	14B (11B to 19B)	14B (12B to 16B)
Direct costs (in JPY)	920B (670B to 1,100B)	1,400B (not applicable)	650B (500B to 770B)	1,100B (not applicable)
Indirect costs (in JPY)	5,300B (3,900B to 7,400B)	4,400B (3,500B to 5,200B)	1,300B (1,000B to 1,800B)	1,300B (1,200B to 1,500B)

Abbreviations: CVD, cardiovascular disease; CHD, coronary heart disease; UI, uncertainty interval; JPY, Japanese yen; USD, US dollars.

Estimates of direct costs for 2019 were used as a reference and do not have any uncertainty.

Supplementary Table S2. Contribution (95% UI) of cumulative changes in all modelled CVD risk factors[1] on the CPPs, CYPPs, net direct and indirect costs saved of CVD (CHD plus stroke) from 2001 to 2019 in Japan estimated by IMPACT$_{NCD-JPN}$

Cumulative values from 2001 to 2019	Men	Women
CPPs	490,000 (310,000 to 730,000)	350,000 (240,000 to 570,000)
CYPPs	1,400,000 (800,000 to 2,200,000)	1,100,000 (670,000 to 1,800,000)
Net saved direct costs (in USD)	4.9B (3.5B to 6.5B)	3.7B (2.7B to 5.1B)
Net saved indirect costs (in USD)	130B (98B to 180B)	25B (16B to 34B)
Net saved direct costs (in JPY)	470B (330B to 620B)	350B (260B to 480B)
Net saved indirect costs (in JPY)	13,000B (9,300B to 17,000B)	2,300B (1,600B to 3,200B)

Abbreviations: UI, uncertainty interval; CVD, cardiovascular disease; CPPs, cases prevented or postponed; CYPPs, case-years prevented or postponed; CHD, coronary heart disease; JPY, Japanese yen; USD, US dollar.

[1] All modelled CVD risk factors consisted of systolic blood pressure, smoking, physical activity, low-density lipoprotein cholesterol, HbA1c, fruit and vegetable consumption, and body mass index. Additionally, SBP, LDL-c, and HbA1c were adjusted for medication use of antihypertensive, cholesterol-lowering, and diabetes, respectively.

Supplementary Table S3. Contribution (95% UI) of cumulative changes in SBP, smoking, LDL-c, and HbA1c on CPPs, CYPPs, and DPPs related to CVD from 2001 to 2019 in Japan estimated by IMPACT$_{NCD-JPN}$

Cumulative values from 2001 to 2019	SBP scenario [1]	Smoking scenario	LDL-c scenario [1]	HbA1c scenario [1]
**Men**				
**CPPs**				
CHD	160,000 (71,000 to 270,000)	130,000 (64,000 to 250,000)	12,000 (4,700 to 26,000)	1,200 (–1,700 to 6,000)
Stroke	190,000 (100,000 to 300,000)	97,000 (49,000 to 170,000)	1,700 (400 to 4,700)	1,100 (–1,800 to 4,100)
CVD	290,000 (200,000 to 440,000)	210,000 (120,000 to 340,000)	12,000 (4,600 to 24,000)	2,300 (–1,600 to 6,600)
**CYPPs**				
CHD	450,000 (160,000 to 860,000)	380,000 (100,000 to 810,000)	35,000 (12,000 to 81,000)	6,000 (–11,000 to 24,000)
Stroke	560,000 (260,000 to 1,100,000)	270,000 (40,000 to 590,000)	2,000 (–5,700 to 16,000)	3,600 (–11,000 to 22,000)
CVD	910,000 (580,000 to 1,400,000)	600,000 (190,000 to 1,100,000)	32,000 (11,000 to 79,000)	9,900 (–15,000 to 36,000)
**DPPs**	180,000 (130,000 to 250,000)	360,000 (310,000 to 450,000)	3,200 (900 to 7,100)	800 (–2,100 to 3,900)
**Women**				
**CPPs**				
CHD	130,000 (59,000 to 260,000)	38,000 (19,000 to 73,000)	15,000 (6,100 to 29,000)	3,700 (900 to 7,800)
Stroke	140,000 (76,000 to 230,000)	46,000 (25,000 to 82,000)	2,200 (300 to 5,300)	2,400 (100 to 8,900)
CVD	240,000 (150,000 to 410,000)	81,000 (48,000 to 130,000)	16,000 (7,000 to 29,000)	5,900 (2,400 to 10,000)
**CYPPs**				
CHD	400,000 (160,000 to 830,000)	140,000 (58,000 to 270,000)	42,000 (16,000 to 89,000)	11,000 (–2,800 to 30,000)
Stroke	400,000 (180,000 to 720,000)	170,000 (69,000 to 320,000)	4,700 (–5,800 to 14,000)	6,000 (–6,700 to 28,000)
CVD	750,000 (470,000 to 1,200,000)	310,000 (160,000 to 500,000)	42,000 (16,000 to 87,000)	17,000 (–3,300 to 37,000)
**DPPs**	170,000 (130,000 to 250,000)	100,000 (83,000 to 130,000)	3,600 (1,300 to 8,200)	2,400 (600 to 5,100)

Abbreviations: UI, uncertainty interval; SBP, systolic blood pressure; CVD, cardiovascular disease; CHD, coronary heart disease; CPPs, cases prevented or postponed; CYPPs, case-years prevented or postponed; DPPs, deaths prevented or postponed; LDL-c, low-density lipoprotein cholesterol.

[1] SBP, LDL-c, and HbA1c were adjusted for medication use of antihypertensive, cholesterol-lowering, and diabetes, respectively.

Supplementary Table S4. Contribution (95% UI) of cumulative changes in BMI, physical activity, and fruit and vegetable consumption on CPPs, CYPPs, and DPPs related to CVD from 2001 to 2019 in Japan estimated by IMPACT$_{\text{NCD-JPN}}$

Cumulative values from 2001 to 2019	BMI scenario	Physical activity scenario	Fruit and vegetable consumption scenario
**Men**			
**CPPs**			
CHD	–5,500 (–12,000 to –2,000)	–2,300 (–5,300 to –400)	–2,700 (–8,900 to –400)
Stroke	–8,100 (–19,000 to –2,900)	–2,600 (–6,300 to –400)	–3,300 (–8,800 to –1,100)
CVD	–13,000 (–25,000 to –6,600)	–4,900 (–9,100 to –1,900)	–5,700 (–14,000 to –2,000)
**CYPPs**			
CHD	–21,000 (–50,000 to –5,000)	–6,800 (–17,000 to –1,400)	–9,900 (–38,000 to –1,100)
Stroke	–36,000 (–82,000 to –12,000)	–9,600 (–26,000 to –1,000)	–14,000 (–39,000 to –2,000)
CVD	–54,000 (–100,000 to –27,000)	–16,000 (–35,000 to –4,900)	–22,000 (–62,000 to –6,600)
**DPPs**	–3,600 (–8,800 to –1,300)	–200 (–3,100 to 5,500)	–1,700 (–4,400 to –300)
**Women**			
**CPPs**			
CHD	–700 (–2,600 to 200)	–4,500 (–9,900 to –1,900)	–2,000 (–6,700 to 0)
Stroke	–2,100 (–4,600 to –300)	–7,300 (–15,000 to –3,600)	–2,900 (–8,700 to –700)
CVD	–2,600 (–6,000 to –500)	–12,000 (–22,000 to –7,000)	–5,000 (–12,000 to –1,300)
**CYPPs**			
CHD	–2,100 (–8,200 to 2,100)	–20,000 (–47,000 to –5,400)	–7,400 (–23,000 to 500)
Stroke	–8,200 (–19,000 to 100)	–30,000 (–68,000 to –9,600)	–10,000 (–39,000 to –300)
CVD	–10,000 (–24,000 to –800)	–49,000 (–93,000 to –21,000)	–19,000 (–50,000 to –4,000)
**DPPs**	–800 (–2,300 to 0)	–12,000 (–18,000 to –7,000)	–1,200 (–3,400 to 0)

Abbreviations: UI, uncertainty interval; BMI, body mass index; CVD, cardiovascular disease; CHD, coronary heart disease; CPPs, cases prevented or postponed; CYPPs,

case-years prevented or postponed; DPPs, deaths prevented or postponed.

Supplementary Table S5. Contribution (95% UI) of cumulative changes in SBP, smoking, LDL-c, and HbA1c on net gained QALYs, and net direct and indirect costs saved related to CVD from 2001 to 2019 in Japan estimated by IMPACT$_{NCD\text{-}JPN}$

Cumulative values from 2001 to 2019	SBP scenario [1]	Smoking scenario	LDL-c scenario [1]	HbA1c scenario [1]
**Men**				
**Net gained QALYs**	860,000 (570,000 to 1,300,000)	1,600,000 (1,300,000 to 2,300,000)	16,000 (6,000 to 34,000)	–370,000 (–390,000 to –360,000)
**Net saved direct costs**				
CHD (in USD)	1,200M (600M to 2,000M)	1,200M (510M to 2,000M)	88M (32M to 160M)	19M (–28M to 77M)
Stroke (in USD)	2,200M (1,100M to 3,500M)	580M (–220M to 1,300M)	9M (–25M to 65M)	12M (–81M to 90M)
CVD (in USD)	3,300M (2,300M to 4,600M)	1,700M (620M to 2,800M)	97M (32M to 210M)	26M (–56M to 140M)
CHD (in JPY)	110B (57B to 190B)	110B (48B to 190B)	8·3B (3B to 15B)	1·8B (–2·7B to 7·3B)
Stroke (in JPY)	210B (110B to 330B)	55B (–21B to 120B)	0·8B (–2·4B to 6·1B)	1·1B (–7·6B to 8·5B)
CVD (in JPY)	310B (220B to 440B)	170B (59B to 270B)	9.2B (3B to 20B)	2.5B (–5.3B to 13B)
**Net saved indirect costs**				
CHD (in USD)	36,000M (20,000M to 66,000M)	51,000M (29,000M to 81,000M)	330M (–1,500M to 2,500M)	950M (–590M to 3,300M)
Stroke (in USD)	25,000M (16,000M to 40,000M)	24,000M (15,000M to 37,000M)	41M (–260M to 530M)	290M (–360M to 1,400M)
CVD (in USE)	63,000M (42,000M to 91,000M)	73,000M (51,000M to 110,000M)	510M (–1,500M to 2,600M)	1,400M (–440M to 4,000M)
CHD (in JPY)	3,400B (1,900B to 6,300B)	4,800B (2,800B to 7,700B)	31B (–140B to 240B)	90B (–56B to 310B)
Stroke (in JPY)	2,400B (1,600B to 3,800B)	2,200B (1,400B to 3,500B)	3·9B (–25B to 50B)	28B (–34B to 140B)
CVD (in JPY)	5,900B (4,000B to 8,600B)	6,900B (4,900B to 10,000B)	48B (–140B to 250B)	130B (–42B to 380B)
**Women**				
**Net gained QALYs**	820,000 (570,000 to 1,200,000)	500,000 (370,000 to 690,000)	18,000 (7,200 to 36,000)	–13,000 (–24,000 to 1,900)
**Net saved direct costs**				
CHD (in USD)	720M (390M to 1,200M)	290M (170M to 470M)	69M (31M to 120M)	20M (–5M to 43M)

Stroke (in USD)	1,900M (980M to 3,300M)	560M (190M to 970M)	29M (–35M to 82M)	31M (–40M to 150M)
CVD (in USE)	2,700M (1,800M to 3,900M)	880M (460M to 1,200M)	94M (26M to 170M)	54M (–24M to 160M)
CHD (in JPY)	68B (37B to 110B)	27B (16B to 45B)	6·5B (2·9B to 12B)	1·9B (–0·5B to 4·1B)
Stroke (in JPY)	180B (93B to 310B)	53B (18B to 92B)	2·7B (–3·3B to 7·7B)	3B (–3·8B to 15B)
CVD (in JPY)	260B (170B to 370B)	83B (44B to 120B)	8.9B (2.5B to 16B)	5.1B (–2.2B to 15B)
**Net saved indirect costs**				
CHD (in USD)	4,100M (1,300M to 8,900M)	6,800M (3,500M to 11,000M)	62M (–460M to 450M)	82M (–25M to 480M)
Stroke (in USD)	6,300M (3,500M to 11,000M)	6,900M (3,700M to 11,000M)	31M (–34M to 200M)	67M (–110M to 340M)
CVD (in USD)	11,000M (6,400M to 17,000M)	13,000M (9,500M to 19,000M)	91M (–450M to 510M)	190M (–64M to 640M)
CHD (in JPY)	390B (130B to 840B)	640B (330B to 1,000B)	5·8B (–44B to 42B)	7·7B (–2·3B to 45B)
Stroke (in JPY)	600B (330B to 1,100B)	660B (350B to 1,000B)	2·9B (–3·3B to 19B)	6·3B (–10B to 32B)
CVD (in JPY)	1,000B (610B to 1,600B)	1,300B (900B to 1,800B)	8.6B (–42B to 49B)	18B (–6.1B to 61B)

Abbreviations: UI, uncertainty interval; SBP, systolic blood pressure; CVD, cardiovascular disease; CHD, coronary heart disease; LDL-c, low-density lipoprotein cholesterol; QALYs; quality-adjusted life years; JPY, Japanese yen; USD, US dollars.

[1] SBP, LDL-c, and HbA1c were adjusted for medication use of antihypertensive, cholesterol-lowering, and diabetes, respectively.

Supplementary Table S6. Contribution (95% UI) of cumulative changes in BMI, physical activity, and fruit and vegetable consumption on net gained QALYs, and net direct and indirect costs saved related to CVD from 2001 to 2019 in Japan estimated by IMPACT$_{NCD\text{-}JPN}$

Cumulative values from 2001 to 2019	BMI scenario	Physical activity scenario	Fruit and vegetable consumption scenario
**Men**			
**Net gained QALYs**	–600,000 (–770,000 to –450,000)	–7,300 (–18,000 to 0)	–11,000 (–29,000 to –3,000)
**Net saved direct costs**			
CHD (in USD)	–62M (–140M to –14M)	–20M (–46M to –2·6M)	–27M (–92M to –3·8M)
Stroke (in USD)	–110M (–200M to –37M)	–27M (–67M to –1·1M)	–49M (–120M to –4·1M)
CVD (in USD)	–180M (–300M to –86M)	–48M (–99M to –13M)	–73M (–190M to –16M)
CHD (in JPY)	–5·9B (–13B to –1·3B)	–1·9B (–4·4B to –0·2B)	–2·6B (–8·7B to –0·4B)
Stroke (in JPY)	–11B (–19B to –3·5B)	–2·6B (–6·4B to –0·1B)	–4·6B (–12B to –0·4B)
CVD (in JPY)	–17B (–28B to –8.2B)	–4.6B (–9.4B to –1.2B)	–6.9B (–18B to –1.5B)
**Net saved indirect costs**			
CHD (in USD)	–4,000M (–9,300M to –640M)	–800M (–2,400M to –12M)	–740M (–3,900M to –4·4M)
Stroke (in USD)	–2,800M (–6,400M to –760M)	–640M (–2,000M to –43M)	–710M (–2,100M to –68M)
CVD (in USD)	–7,100M (–13,000M to –2,700M)	–1,500M (–3,600M to –290M)	–1,700M (–4,700M to –85M)
CHD (in JPY)	–370B (–880B to –61B)	–75B (–230B to –1·1B)	–70B (–370B to –0·4B)
Stroke (in JPY)	–260B (–610B to –72B)	–60B (–190B to –4·1B)	–67B (–200B to –6·5B)
CVD (in JPY)	–670B (–1,200B to –250B)	–140B (–340B to –28B)	–160B (–450B to –8B)
**Women**			
**Net gained QALYs**	–33,000 (–46,000 to –7,000)	–56,000 (–93,000 to –34,000)	–8,500 (–23,000 to –1,700)
**Net saved direct costs**			
CHD (in USD)	–4·4M (–16M to 4·7M)	–44M (–88M to –19M)	–14M (–49M to 0·5M)
Stroke (in USD)	–24M (–55M to 6·1M)	–130M (–240M to –40M)	–48M (–160M to 1M)

CVD (in USD)	–29M (–63M to 3.2M)	–170M (–290M to –74M)	–63M (–190M to –8.6M)
CHD (in JPY)	–0·4B (–1·5B to 0·4B)	–4·2B (–8·3B to –1·8B)	–1·3B (–4·7B to 0)
Stroke (in JPY)	–2·3B (–5·2B to 0·6B)	–12B (–23B to –3·8B)	–4·5B (–15B to 0·1B)
CVD (in JPY)	–2.8B (–5.9B to 0·3B)	–16B (–27B to –7B)	–6B (–18B to –0·8B)
**Net saved indirect costs**			
CHD (in USD)	–270M (–1,200M to 94M)	–190M (–730M to –29M)	–40M (–710M to 0)
Stroke (in USD)	–480M (–1,600M to 4·9M)	–450M (–1,100M to –130M)	–170M (–710M to –20M)
CVD (in USD)	–850M (–1,800M to –66M)	–700M (–1,400M to –260M)	–320M (–1,100M to –32M)
CHD (in JPY)	–26B (–120B to 8·9B)	–18B (–69B to –2·8B)	–3·8B (–67B to 0)
Stroke (in JPY)	–45B (–150B to 0·5B)	–43B (–110B to –12B)	–16B (–67B to –1·9B)
CVD (in JPY)	–81B (–170B to –6.3B)	–67B (–140B to –25B)	–31B (–100B to –3.1B)

Abbreviations: UI, uncertainty interval; BMI, body mass index; CVD, cardiovascular disease; CHD, coronary heart disease; QALYs; quality-adjusted life years; JPY, Japanese yen; USD, US dollars.

Supplementary Table S7. Contribution (95% UI) of cumulative changes in all modelled CVD risk factors[1] on the net direct and indirect costs saved of CVD (CHD plus stroke), CHD, and stroke from 2001 to 2019 in Japan estimated by IMPACT$_{NCD-JPN}$

Cost types	Cumulative net saved direct costs from 2001 to 2019		Cumulative net saved indirect costs from 2001 to 2019	
Age groups	30-64 years	≥65 years	30-64 years	≥65 years
**Men**				
CHD (in USD)	1.2B (0·8B to 1.7B)	1.1B (0·6B to 1.8B)	71B (44B to 110B)	14B (8.2B to 21B)
Stroke (in USD)	1.4B (0·9B to 2.2B)	1B (0·1B to 2B)	39B (28B to 65B)	7.1B (4.5B to 12B)
CVD (in USD)	2.6B (1.9B to 3.4B)	2.2B (1.2B to 3.1B)	110B (81B to 150B)	22B (15B to 29B)
CHD (in JPY)	110B (75B to 170B)	100B (55B to 170B)	6,700B (4,200B to 11,000B)	1,300B (780B to 2,000B)
Stroke (in JPY)	140B (86B to 210B)	98B (11B to 190B)	3,700B (2,700B to 6,200B)	670B (430B to 1,100B)
CVD (in JPY)	250B (180B to 330B)	210B (110B to 290B)	11,000B (7,700B to 15,000B)	2,100B (1,400B to 2,700B)
**Women**				
CHD (in USD)	0·3B (0·1B to 0·4B)	0·9B (0·6B to 1.3B)	8.8B (4.9B to 17B)	2.5B (1.3B to 3.9B)
Stroke (in USD)	0·8B (0·5B to 1.2B)	1.6B (0·9B to 2.8B)	11B (6.2B to 17B)	2.6B (1.5B to 4.2B)
CVD (in USD)	1.1B (0.80·8B to 1.5B)	2.6B (1.8B to 3.6B)	20B (12B to 28B)	5.2B (3.4B to 7B)
CHD (in JPY)	25B (14B to 41B)	84B (56B to 120B)	830B (460B to 1,600B)	230B (120B to 370B)
Stroke (in JPY)	76B (46B to 120B)	160B (87B to 260B)	1,000B (590B to 1,600B)	240B (140B to 400B)
CVD (in JPY)	100B (71B to 150B)	250B (170B to 340B)	1,900B (1,200B to 2,700B)	490B (320B to 660B)

Abbreviations: UI, uncertainty interval; CVD, cardiovascular disease; CHD, coronary heart disease; CPPs, cases prevented or postponed; CYPPs, case–years prevented or postponed; JPY, Japanese yen; USD, US dollar.

[1] All modelled CVD risk factors consisted of systolic blood pressure, smoking, physical activity, low–density lipoprotein cholesterol, HbA1c, fruit and vegetable consumption, and body mass index. Additionally, SBP, LDL-c, and HbA1c were adjusted for medication use of antihypertensive, cholesterol-lowering, and diabetes, respectively.

Supplementary Table S8. Direct and indirect costs of CVD (CHD plus stroke), CHD, and stroke (95% UI) in the base-case scenario estimated by IMPACT$_{NCD-JPN}$

**Cost types**	**Direct cost**		**Indirect cost**	
**Age groups**	**30-64 years**	**≥65 years**	**30-64 years**	**≥65 years**
**Men in 2001**				
CHD (in USD)	1.3B (0·8B to 1.9B)	2B (1.3B to 3.3B)	23B (13B to 37B)	5.2B (3B to 7.9B)
Stroke (in USD)	2B (1.5B to 2.6B)	4.1B (3.2B to 5.3B)	21B (15B to 30B)	7B (5.1B to 9.3B)
CVD (in USD)	3.3B (2.4B to 4.1B)	6.3B (4.7B to 7.8B)	44B (31B to 63B)	12B (9.1B to 16B)
CHD (in JPY)	120B (77B to 180B)	190B (120B to 310B)	2,200B (1,200B to 3,500B)	490B (280B to 750B)
Stroke (in JPY)	190B (140B to 250B)	390B (300B to 500B)	2,000B (1,400B to 2,800B)	660B (480B to 880B)
CVD (in JPY)	320B (230B to 390B)	600B (450B to 740B)	4,100B (3,000B to 6,000B)	1,200B (860B to 1,500B)
**Women in 2001**				
CHD (in USD)	0·3B (0·2B to 0·4B)	0·8B (0·5B to 1.3B)	2.5B (0·9B to 5.1B)	1.2B (0·8B to 2.1B)
Stroke (in USD)	1.3B (1B to 1.6B)	4.4B (3.5B to 5.5B)	5.8B (4B to 9B)	4.4B (3.5B to 5.5B)
CVD (in USD)	1.6B (1.2B to 1.9B)	5.2B (4.1B to 6.2B)	8.2B (6.1B to 13B)	5.6B (4.4B to 7B)
CHD (in JPY)	26B (15B to 38B)	78B (46B to 130B)	240B (85B to 480B)	110B (74B to 200B)
Stroke (in JPY)	120B (95B to 150B)	420B (340B to 520B)	550B (380B to 850B)	410B (330B to 520B)
CVD (in JPY)	150B (110B to 180B)	500B (390B to 590B)	770B (570B to 1,200B)	530B (410B to 660B)
**Men in 2019**				
CHD (in USD)	1.4B (1.4B to 1.4B)	3.8B (3.8B to 3.8B)	16B (10B to 23B)	5.7B (4.4B to 7.1B)
Stroke (in USD)	2.2B (2.2B to 2.2B)	7.6B (7.6B to 7.6B)	15B (11B to 19B)	9.3B (8.4B to 10B)
CVD (in USD)	3.6B (3.6B to 3.6B)	11B (11B to 11B)	31B (23B to 40B)	15B (13B to 17B)
CHD (in JPY)	130B (130B to 130B)	360B (360B to 360B)	1,500B (990B to 2,200B)	540B (420B to 670B)
Stroke (in JPY)	210B (210B to 210B)	720B (720B to 720B)	1,400B (1,100B to 1,800B)	880B (800B to 950B)
CVD (in JPY)	340B (340B to 340B)	1100B (1100B to 1100B)	3,000B (2,200B to 3,700B)	1,400B (1,200B to 1,600B)
**Women in 2019**				
CHD (in USD)	0·3B (0·3B to 0·3B)	1.9B (1.9B to 1.9B)	1.3B (0·2B to 2.7B)	1.3B (1B to 1.7B)

Stroke (in USD)	1.3B (1.3B to 1.3B)	8.1B (8.1B to 8.1B)	4B (2.6B to 5.1B)	7.3B (6.8B to 7.8B)
CVD (in USD)	1.6B (1.6B to 1.6B)	10B (10B to 10B)	5.3B (3.8B to 7.1B)	8.6B (8.2B to 9.2B)
CHD (in JPY)	25B (25B to 25B)	180B (180B to 180B)	130B (17B to 250B)	120B (99B to 160B)
Stroke (in JPY)	120B (120B to 120B)	760B (760B to 760B)	380B (250B to 480B)	690B (640B to 740B)
CVD (in JPY)	150B (150B to 150B)	940B (940B to 940B)	500B (360B to 670B)	820B (770B to 870B)

Abbreviations: CVD, cardiovascular disease; CHD, coronary heart disease; UI, uncertainty interval; JPY, Japanese yen; USD, US dollar.

Supplementary Table S9. Estimated incident cases and incidence rates of CHD and stroke subtypes in people aged 35 and over, derived from population-based incidence rates and subtype proportions, combined with CHD and stroke estimates generated by the base-case scenario of IMPACT$_{NCD-JPN}$ model.

	Men		Women	
	2001	2019	2001	2019
**Incident cases in people aged 35 years old and older**				
CHD				
Total [a]	137000	183000	82600	145000
AMI [b]	41500	56400	16500	25800
CHD other than AMI [a, b]	95500	126600	66100	119200
AMI plus SCD [c]	45500	61400	20500	31500
CHD other than AMI + SCD [a, c]	91500	121600	62100	113500
Stroke				
Total [a]	153140	144070	120350	119650
Cerebral infarction [a, d]	103000	100000	66200	70900
Intracerebral haemorrhage [a, d]	39100	35100	32600	30700
Subarachnoid haemorrhage [a, d]	9500	7270	20500	16700
Undetermined type [a, d]	1540	1700	1050	1350
**Incidence rates per 100,000 in people aged 35 years old and older**				
CHD				
Total [a]	390	449	211	323
AMI [b]	118	139	42.2	57.5
CHD other than AMI [a, b]	272	310	168.8	265.5
AMI plus SCD [c]	129	151	52.4	70.1
CHD other than AMI + SCD [a, c]	261	298	158.6	252.9
Stroke				
Total [a]	435.35	354.27	307.48	266.6
Cerebral infarction [a, d]	293	246	169	158
Intracerebral haemorrhage [a, d]	111	86.2	83.3	68.4
Subarachnoid haemorrhage [a, d]	27	17.9	52.5	37.2
Undetermined type [a, d]	4.35	4.17	2.68	3

Abbreviations: CVD, cardiovascular disease; CHD, coronary heart disease; AMI, acute myocardial infarction; SCD, sudden cardiac death.

a This was based on estimates generated by the base-case scenario of IMPACT$_{NCD-JPN.}$

b This was based on the reported population-based incidence rates.[1]

c This was based on the reported population-based incidence rates.[2]

d This was based on the reported population-based incidence rates.[3]

To enhance the clarity and interpretability of the modelled CVD burden, we present estimates of incident case numbers and incidence rates for selected CHD and stroke subtypes. While the IMPACT$_{NCD-JPN}$ model simulates total CHD and total stroke only—due to the lack of subtype-specific risk factor coefficients based on nationally representative data—these additional incidence cases and rates were calculated using established, population-based incidence rates from previous studies in Japanese local areas. Specifically, AMI incidence was estimated using age- and sex-specific rates reported in two cohort studies[1,2], and cases of other CHD were derived by subtracting AMI estimates from the total CHD burden obtained from the base-case scenario of IMPACT$_{NCD-JPN}$. For stroke subtypes (cerebral infarction, intracerebral haemorrhage, and subarachnoid haemorrhage), proportions reported in a population-based study[3] were applied to the modelled total stroke incidence. These estimates are limited to individuals aged 35 years old and older due to age range restrictions in the source data.[1,3] The results should be interpreted with caution given the indirect nature of these calculations and the underlying assumptions involved.

1. Sawayama Y, Takashima N, Harada A, et al. Incidence and In-Hospital Mortality of Acute Myocardial Infarction: A Report from a Population-Based Registry in Japan. J Atheroscler Thromb 2023; 30: 1407–19.
2. Ogata S, Marume K, Nakai M, et al. Incidence Rate of Acute Coronary Syndrome Including Acute Myocardial Infarction, Unstable Angina, and Sudden Cardiac Death in Nobeoka City for the Super-Aged Society of Japan. Circ J 2021; 85: 1722–30.
3. Takashima N, Arima H, Kita Y, et al. Incidence, Management and Short-Term Outcome of Stroke in a General Population of 1.4 Million Japanese - Shiga Stroke Registry. Circ J 2017; 81: 1636–46.
