# Supplementary material for: Quantifying the contributions of cardiovascular risk factors to cardiovascular disease trends in 21st century Japan: a microsimulation study
Source: Lancet Reg Health West Pac. 2025 Jul 8;60:101623. doi: 10.1016/j.lanwpc.2025.101623 (PMC12274934; doi:10.1016/j.lanwpc.2025.101623)
Supplement: Translated Abstract–Japanese [file mmc2.docx]

**Editor disclaimer:**

This translation in Japanese was submitted by the authors and we reproduce it as supplied. It has not been peer reviewed. Our editorial processes have only been applied to the original abstract in English, which should serve as reference for this manuscript.

**背景：** 21世紀に入って以降、日本においてLDLコレステロール（LDL-c）や肥満など、脳卒中を含む循環器病（CVD）のリスク要因に関する改善傾向が停滞または悪化しており、CVD負荷の減少速度が鈍化する可能性が懸念されている。本研究では、2001年から2019年にかけて、日本国内におけるCVDリスク要因の分布変化がCVD負荷に与えた影響を定量的に評価することを目的とした。

**方法：**検証済みモデル「IMPACT_NCD-JPN_」を用いて、マイクロシミュレーションと反事実シナリオ（counterfactual）分析を実施した。なお、IMPACT_NCD-JPN_は実際に観測されたデータやメタアナリシスを含む既存エビデンスをデータリソースとして構築されている。本モデルでは30〜99歳の合成日本人口を対象に、7つのCVDリスク要因に関するライフコースデータをシミュレートし、CVD発症率、死亡率、医療経済への影響を推計した。ベースケースシナリオでは実際に観察されたリスク要因の推移を反映し、反事実シナリオでは2001年時点のリスク水準が維持されたと仮定した。主要評価項目は、脳卒中および冠動脈性心疾患（CHD）を含む全国レベルでのCVD発症数とした。

**結果：** 2001年から2019年の間に、収縮期血圧（SBP）および喫煙率は大幅に低下（男性／女性それぞれ6.8／7.2 mmHg、18.4／6.8%の減少）した。一方で、LDL-c、HbA1c、体格指数（BMI）、身体活動、果物・野菜摂取量の改善は限定的、または悪化傾向であった。ベースケースおよび反事実シナリオにおいて、IMPACT_NCD-JPN_モデルによりCVD発症数を推計し、両者の差分からCVDリスク要因の分布変化の影響を定量化した。その結果、2001年から2019年の累積で、全国で840,000　(95% uncertainty interval: 540,000–1,300,000)　件のCVD症例が予防または発症遅延されたと推定された。要因別の寄与では、SBPが540,000、喫煙状況が280,000、LDL-cが27,000、HbA1cが7,900、BMIが-15,000、PAが-16,000、FV摂取量が-11,000の寄与であった（負の値は発症増加数を示す）。

**解釈：** 2001年〜2019年における日本のCVD負荷減少の主因は、ＳＢＰと喫煙率の低下であった。LDL-cおよびHbA1cの改善の効果は限定的であった。しかし、BMIの上昇および身体活動と果物・野菜摂取量の低さは、これらの改善効果を一部相殺していた。
